# Ionomic Variation Among Tissues in Fallow Deer (*Dama dama*) by Sex and Age

**DOI:** 10.1007/s12011-023-03724-x

**Published:** 2023-06-08

**Authors:** Elke Wenting, Henk Siepel, Melanie Christerus, Patrick A. Jansen

**Affiliations:** 1 Department of Environmental Sciences, Wageningen University and Research, Box 47, Wageningen, 6700 AA The Netherlands; 2 Radboud Institute for Biological and Environmental Sciences, Department of Animal Ecology and Physiology, Radboud University, Box 9010, Nijmegen, 6500 GL the Netherlands; 3 Smithsonian Tropical Research Institute, Balboa, Ancon, Panama

**Keywords:** ionomics, minerals, trace elements, Fallow deer, wildlife

## Abstract

(1) In mammals, the mineral nutrient and trace elemental composition of the body - the ionome - differs among individuals. It has been hypothesized that these differences may be related to age and sex, both for ecotoxic and essential elements. (2) We investigated whether and how intraspecific ionomic variation is related to age and sex in Fallow deer (*Dama dama*). We tested the predictions that concentrations of ecotoxic elements increase with age, that ionomic variation is lower among young individuals than among older individuals, and that reproductive females (does) have the lowest concentrations of essential elements. (3) Culled animals of different sex and age were obtained from a single protected area. The animals were dissected to collect 13 tissues, and concentrations of 22 different elements were measured in a sample of each tissue. (4) We described substantial ionomic variation between individuals. Some of this variation was related to age and sex, as predicted. Based on the limited existing knowledge on chemical element allocation and metabolism in the body, sex-related differences were more difficult to interpret than age-related differences. Since reference values are absent, we could not judge about the consequences of the elemental values that we found. (5) More extensive ionomic surveys, based on a wide range of elements and tissues, are needed to enlarge the understanding of within-species ionomic variation and potential biological, ecological, and metabolic consequences.

**Supplementary Information:**

The online version contains supplementary material available at 10.1007/s12011-023-03724-x.

## Introduction

The mineral nutrient and trace elemental composition of organisms - the ionome [1, 2] - is an important expression of their physiological state [1], relating to a variety of biological and ecological processes, including life history plasticity [3, 4], population growth [5, 6], foraging ecology [7], and carrion decomposition [8]. The ionome of animals - unlike plants - has long been assumed to be more or less constant, i.e. ‘homeostatic’ [9]. This assumption was based on observations of nearly constant ratios of carbon (C) to nitrogen (N) to phosphorous (P) [4], three elements that are most often considered in studies considering ecological stoichiometry, i.e. the study of the balance of energy and multiple chemical elements in ecological interactions [10–12].

However, recent studies have shown that the assumption of ionomic homeostasis does not hold for chemical elements in general, and that many elements are in fact much more scattered throughout the whole body. Wenting et al. [8] examined the elemental composition of Fallow deer (*Dama dama*) and Eurasian otter (*Lutra lutra*) by measuring twelve elemental concentrations in twelve different organs and tissues and found differences in elemental concentration within and between the species. Ma et al. [13] found, based on four tissues of 26 species and 18 elements, lineage-specific patterns and correlations between elements, tissues, and body mass. The causes of this variation within and among species remain unknown. Exploring drivers of ionomic variability requires extensive studies dealing with multiple chemical elements, tissues, and organs.

It has been suggested that age and sex are important drivers of intraspecific ionomic variation. Bioaccumulation of toxic elements - e.g. aluminium (Al), cadmium (Cd) and lead (Pb) - increases with exposure time and thus age [14]. Young animals obtain essential elements from their mother via the placenta and milk [15], and may thus show less ionomic variation than adults, which must acquire these typically scarce elements through feeding [16]. Particularly reproductive females, which transfer elements to their young, may show more ionomic variation than younger animals. However, to our knowledge, these relationships have never been examined for a wide range of elements across multiple tissues. Thus, the role of sex and age as driver of ionomic variation in mammals remains vastly unexplored.

Some variation has been described for specific tissue-element combinations [e.g. 17–25]. For instance, Demesko et al. [26] found that concentrations of manganese (Mn) and zinc (Zn) in the teeth of Roe deer (*Capreolus capreolus*) increased with age. Lazarus et al. [27] found sex-related differences in Cd, iron (Fe), and Zn concentration in the kidney cortex, and for Pb in the jawbone, but did not report the magnitude of these differences. Cygan-Szczegielniak & Stasiak [28] measured higher concentrations of heavy metals in the liver of Roe deer females compared to younger individuals. However, these studies all considered only the few tissues and elements that are commonly used as bioindicators, such as Cd and other ecotoxic heavy metals [e.g. 29–32]. Thus, the overall magnitude of age- and sex-related variation, incorporating a wide range of elements and tissues, remains unknown.

The aim of this study was to describe whether and how intraspecific ionomic variation could be related to age and sex. Our approach was to measure the ionome, including a wide range of elements and tissues, of multiple individuals of Fallow deer, belonging to different sex and age groups, that were collected from a single protected area. We examined five predictions: (1) the total concentrations of essential elements are lower for reproductive females (does) than for younger females and males in general, while concentrations of toxic elements increase with age and are therefore highest for adults; (2) age-related differences are largest among females, with bioaccumulation of toxic elements increasing with age in tissues that excrete these elements (e.g. liver and kidney) and essential elements decreasing with age due to pregnancy; (3) age-related differences among males are more related to bioaccumulation of toxic elements than differences in essential elements; and (4) the least sex-related differences are found among calves, both for essential and toxic elements; resulting in (5) most sex-related differences being found among yearlings due to increasing age, predominantly as lower concentrations of essential elements for yearling females compared to males due to pregnancy. In addition, we considered other sex and age-related differences and speculated on cause of differences in the context of their biological and physiological role.

## Materials and Methods

### Study Site and Species

The Fallow deer is a terrestrial ungulate herbivore with an adult body weight of 40–80 kg and a non-nomadic lifestyle [33]. After a gestation period of 31 to 32 weeks, a doe gives birth to a single calf. Calves are born in May or June and are weaned after seven to nine months. Yearling females can be pregnant as most females give birth to their first calf in their second year of life [34, 35]. Being an intermediate feeder [36], Fallow deer is an ideal model species for this study as its browsing behaviour might compensate for the low amount of trace elements in the average vegetation.

The freshly culled individuals that we used were obtained from Deelerwoud (52°08’N, 5°89’E). Deelerwoud is a protected area at the Veluwe, the Netherlands, characterized by a gently rolling forest and heathland landscape [37]. It is situated on partly glacier deposits and on cover sands over these deposits (“mineral-poor cover sands”), causing the mineral availability to be limited to very scarce [38–40]. Kuiters [41] found increasing levels of Cd and Pb with age in Red deer (*Cervus elaphus*) and Wild boar (*Sus scrofa*) from the Veluwe area, where concentrations varied over different food types (browse, grasses, dwarf shrubs, acorns, etc.) and over the season. Wolkers et al. [42] also reported that levels of Cd and Pb at the Veluwe increased with age, even to such an extent that liver and kidney of Red deer and Wild boar were seen as unsuitable for human consumption.

### Carcass Collection and Dissection

We obtained twenty fresh Fallow deer carcasses from regular culling in the hunting season 2021–2022 (between October and March). Twelve of these carcasses were females: four calves, four yearlings, and four does. The yearlings and does were pregnant, with different embryotic stages depending on the moment of culling. We were not able to age the does more precisely. The other eight carcasses were males: four calves and four yearlings. We did not include adult males (bucks) because none were culled during our study period. No animals were killed for the purpose of our study. According the Animal Welfare Officer of Wageningen University & Research, our study is not considered as experimentation on animals and thus permitted under Dutch law (Appendix A).

In total, we dissected the carcasses to collect thirteen organs and tissues (henceforth ‘tissues’), belonging to different organ systems: skin and hair; muscle; brain; eyes; lungs; heart; spleen; kidney; liver; pancreas; stomach, including rumen; and intestines. In a shed at Deelerwoud, we dissected seven tissues: skin and hair, muscle, lungs, heart, spleen, kidney, and liver. We also dissected the entire guts - pancreas, stomach, and intestines -, the head - brain and eyes -, and the hind leg - bone -, that we further dissected in the dissection room of Wageningen Environmental Research. We dissected the hind leg into a bone sample by sawing a piece of bone from the lower leg and putting it in boiling water for a few minutes to loosen the remaining tissues, to retain a clean bone sample afterwards that we used in the next step of freeze-drying. We also further dissected the guts and head. All the collected tissues - frozen at minus 18 °C after dissection - were homogenized using a blender, except the bone sample, that was used in its entirety. Three tablespoons − 15–25 g each - of the homogeneous tissue samples were stored in plastic bags in the freezer before we prepared them for chemical analyses. After dissection all carcass remains were returned to the study area.

### Measurements

Before the chemical analyses, we freeze-dried the tissue samples. The dry samples were transported to Radboud University in ice blocks to prevent defrosting. At Radboud University, we used a microwave destruction - aka digestion - method with 5 mL 65% nitric acid (HNO_3_) and 2 mL 30% hydrogen peroxide (H_2_O_2_), after which the tissue samples were ready for measuring the elemental concentrations with Inductively Coupled Plasma Optical Emission Spectroscopy (ICP-OES) and Inductively Coupled Plasma Mass Spectroscopy (ICP-MS).

In total, we measured 22 elemental concentrations: Al, arsenic (As), boron (B), calcium (Ca), Cd, cobalt (Co), chromium (Cr), copper (Cu), Fe, potassium (K), magnesium (Mg), Mn, molybdenum (Mo), sodium (Na), nickel (Ni), P, Pb, sulfur (S), selenium (Se), silicon (Si), strontium (Sr), and Zn. Seven elements were measured using ICP-OES: Ca, K, Mg, Na, P, S, and Si. The other 15 elements were measured using ICP-MS. We used the same devices as Wenting et al. [8]. Correspondingly, the accuracy of these devices was guaranteed by using the following quality controls (QC): Multi element standard IV, Merck 1.11355; Phosphate standard, Merck 1.19898; Sulphate standard, Merck 1.19813; and Silicium standard, Merck 1.70236. The QC matrices were considered to correspond to the sample matrices since for both, any contamination of HNO_3_ and H_2_O_2_ was eliminated by using blanks (see for more details Wenting et al. [8]).

### Statistical Analyses

All statistical analyses were done in R version 4.0.2 [43]. The statistical analyses should be considered as indicative rather than steadfastly; due to the low sample sizes, they have limited meaning. Yet, we believe that the indicative nature is helpful for determining the most notable differences, although we acknowledge that it should be considered as descriptive. For the first prediction - focusing on the total elemental concentrations -, we calculated and visualized the total elemental concentration per element per individual. We used Kruskal-Wallis tests to test for differences between the groups per element with a Bonferroni-corrected alpha of 0.00227. The second prediction - focusing on age-related differences among females - was analyzed with Kruskal-Wallis tests per tissue-element combination. We used Mann-Whitney U tests to analyze each tissue-element combination for the third, fourth and fifth prediction - respectively focusing on age-related differences among males, sex-related differences among calves, and sex-related differences among yearlings. We used the step-up Benjamini and Hochberg procedure [44] to correct for multiple testing using the p.discrete.adjust function of the discreteMTP package [45] in the analyses of the second to fifth prediction. We only visualized the element-tissue combinations that turned out significant for these predictions. See Appendix B for all test statistics.

## Results

**Fig. 1:**
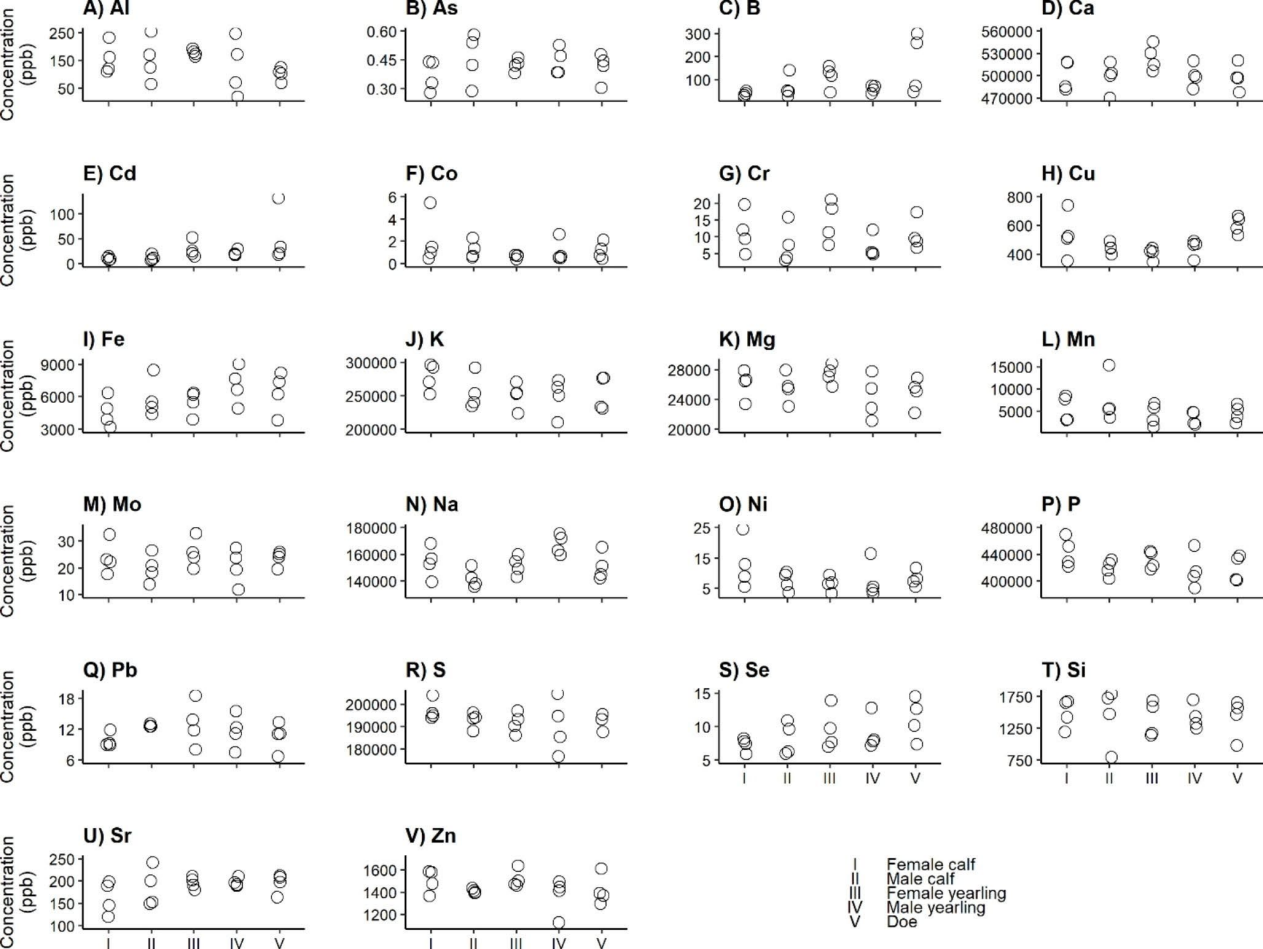
Total elemental concentrations per age-sex group of Fallow deer

**Fig. 2:**
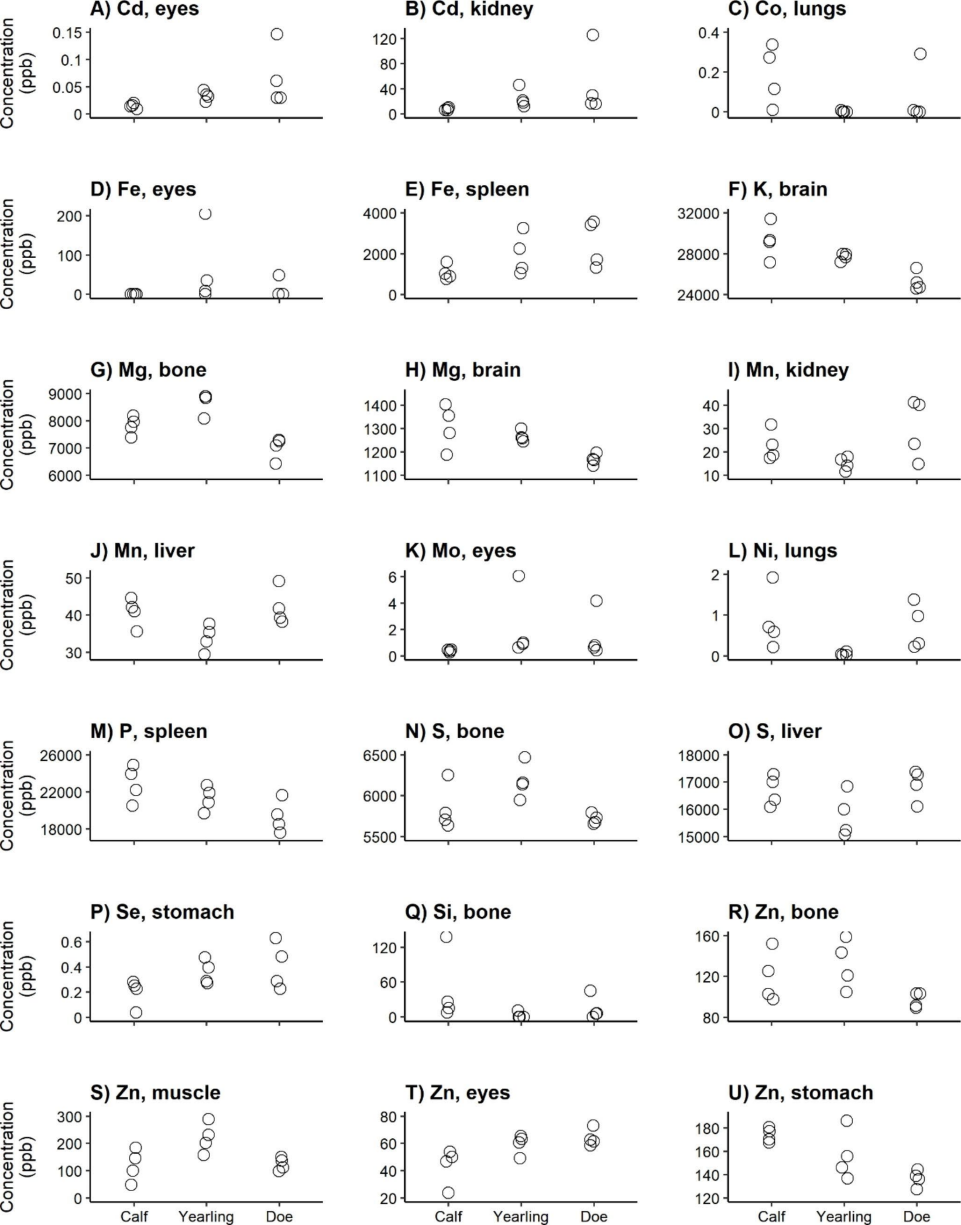
Significantly different tissue-element combinations (according to adjusted p-values using the step-up Benjamini and Hochberg procedure [44]) between age groups in Fallow deer females

**Fig. 3:**
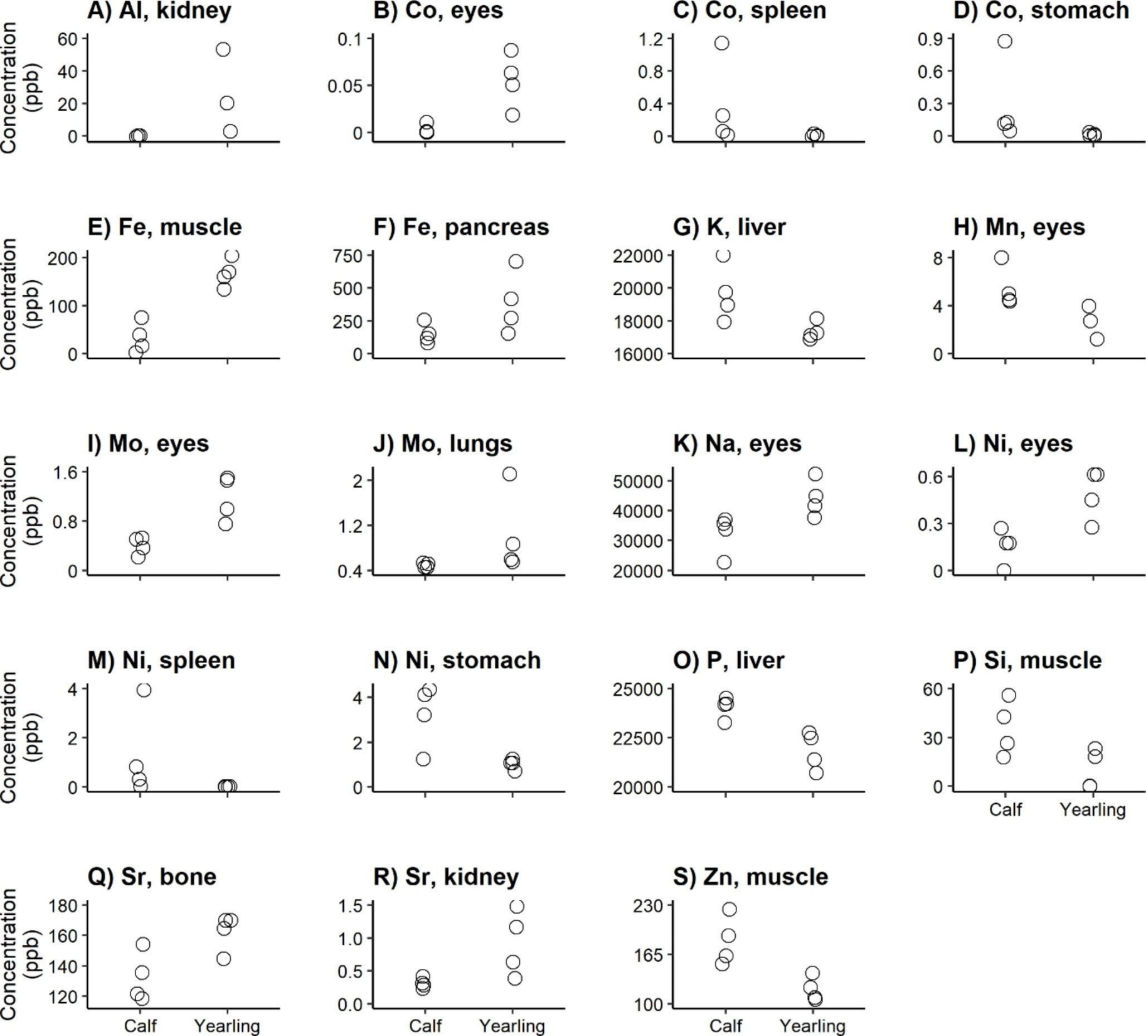
Significantly different tissue-element combinations (according to adjusted p-values using the step-up Benjamini and Hochberg procedure [44]) between age groups in Fallow deer males

**Fig. 4:**
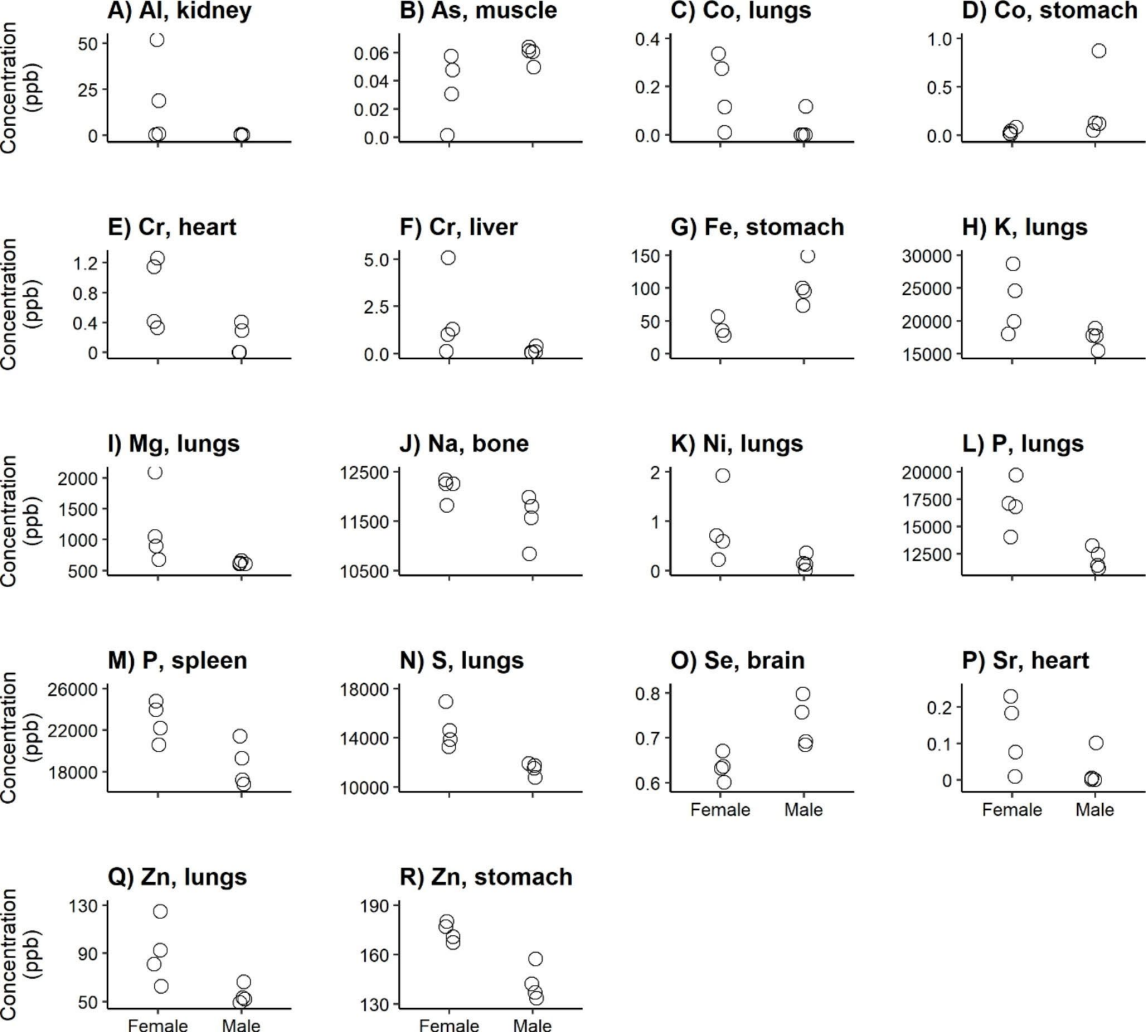
Significantly different tissue-element combinations (according to adjusted p-values using the step-up Benjamini and Hochberg procedure [44]) between sex groups in Fallow deer calves

**Fig. 5:**
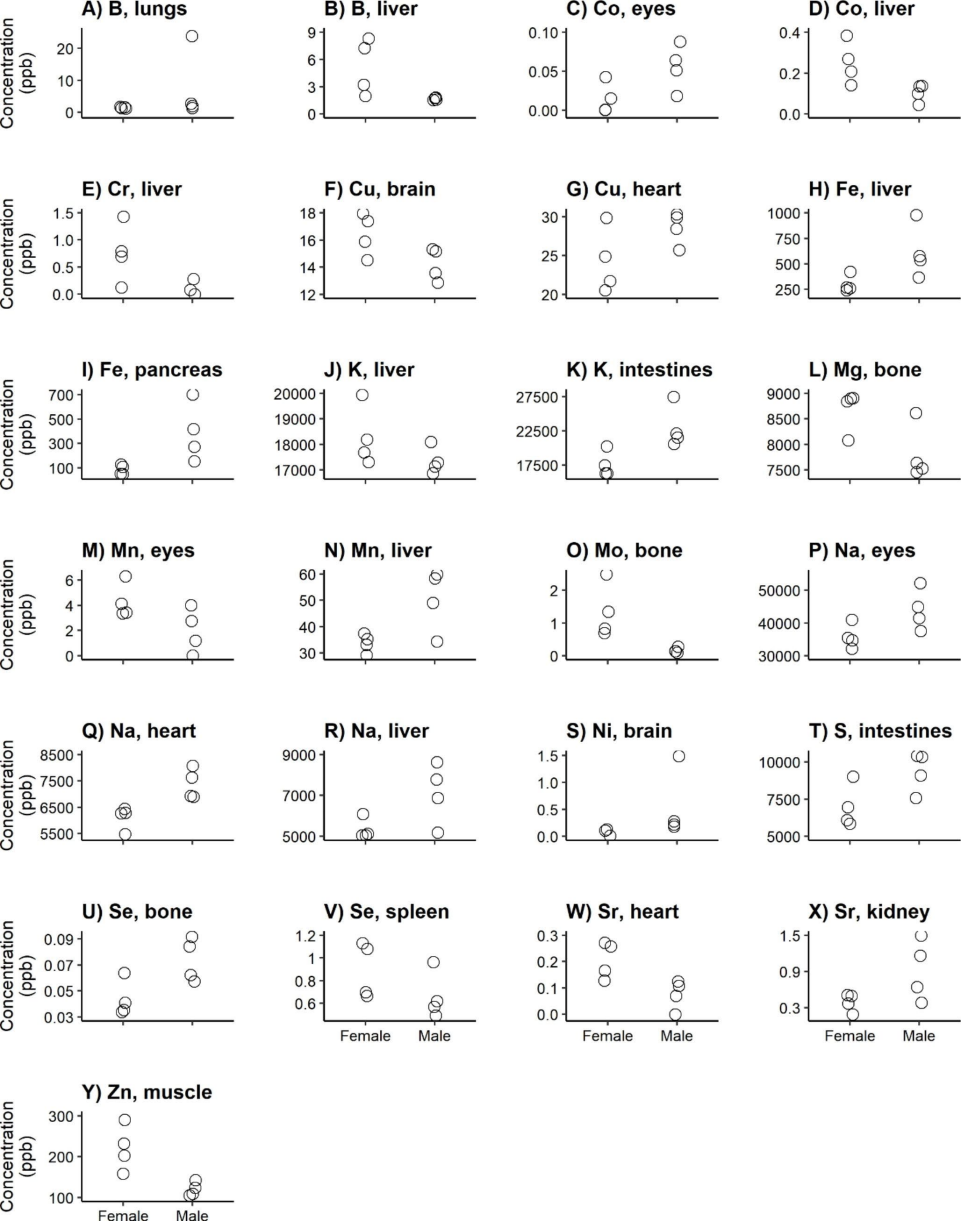
Significantly different tissue-element combinations (according to adjusted p-values using the step-up Benjamini and Hochberg procedure [44]) between sex groups in Fallow deer yearlings

The distribution of the elements over the body differed among the sex-age groups (Appendix C). Although we found no differences in total elemental concentrations per sex-age group (prediction 1; Fig. 1a-v), this is in line with the differences in tissue-element combinations that we found among the sex and age classes (Figs. 2, 3, 4 and 5).

For 21 element-tissue combinations, concentrations differed significantly among age classes of females (prediction 2; Fig. 2a-u). For example, the Cd concentration in eyes and kidney (Fig. 2a-b) was higher for adults than for calves and yearlings. The majority of the other differences were found in essential elements, of which some had higher concentrations in adults (does) - including Fe in spleen (Fig. 2e), Se in stomach (Fig. 2p), and Zn in eyes (Fig. 2t) -, and some had higher concentrations in calves - including K in brain (Fig. 2f), Mg in brain (Fig. 2h), and P in spleen (Fig. 2m). Differences in Zn concentration were found four times, the most often of all elements: in bones (Fig. 2r), muscle (Fig. 2s), eyes (Fig. 2t), and stomach (Fig. 2u).

For 19 tissue-element combinations, concentrations differed significantly between calves and yearling males (prediction 3; Fig. 3a-s). This included just one ecotoxic element: the Al concentration in kidneys was higher in yearlings than in calves (Fig. 3a). For 9 of these tissue-element combinations, calves showed higher concentrations than the yearlings, e.g. Mn in eyes (Fig. 3h), Si in muscle (Fig. 3p), and Zn in muscle (Fig. 3s). Yearlings had higher concentrations than calves in the other tissue-element combinations, e.g. Co in eyes (Fig. 3b), Fe in muscle (Fig. 3e), and Sr in kidney (Fig. 3r).

For 18 element-tissue combinations, concentrations differed among sex classes for calves (prediction 4; Fig. 4a-r). Concentrations in female calves were higher for 14 combinations - including Al in kidney (Fig. 4a), Co in lungs (Fig. 4c), P in lungs and spleen (Fig. 4l + m), and Sr in heart (Fig. 4p) -, compared to four combinations that were higher for male calves - As in muscle (Fig. 4b), Co in stomach (Fig. 4d), Fe in stomach (Fig. 4g), and Se in brain (Fig. 4o). Seven of the 18 differences were found in lungs: Co (Fig. 4c), K (Fig. 4h), Mg (Fig. 4i), Ni (Fig. 4k), P (Fig. 4l), S (Fig. 4n), and Zn (Fig. 4q).

For 25 element-tissue combinations, concentrations differed among sex classes for yearlings (prediction 5; Fig. 5a-y). Female yearlings had the highest concentrations for 11 of these element-tissue combinations - including B in liver (Fig. 5b), Cr in liver (Fig. 5e), Mn in eyes (Fig. 5m), and Zn in muscle (Fig. 5y). Yearling males had higher concentrations for the other 14 significant element-tissue combinations - including Cu in heart (Fig. 5g), Fe in pancreas (Fig. 5i), K in intestines (Fig. 5k), and Sr in kidney (Fig. 5x). All these differences were for essential elements.

## Discussion

This study aimed to determine whether and how ionomic variation, based on a wide range of elements and tissues, is influenced by age and sex. Fallow deer, collected from a single protected area, was used as a model species and we analyzed multiple individuals belonging to different age and sex classes. We predicted the total concentrations of essential elements to be lowest and the accumulation of toxic elements to be highest for does (prediction 1). The distribution over the tissues seemed to differ among the groups (Appendix B), but we found no differences in total concentrations per element between the age and sex classes (Fig. 1a-v). As predicted, we indeed found differences in concentrations between sex and age classes for a substantial number of tissue-element combinations (Figs. 2, 3, 4 and 5). We speculated about the biological and physiological role of the differences that we described.

### Age-related Differences in Females

We predicted that most age-related differences occur within females, with bioaccumulation of toxic elements increasing with age, mostly in tissues that excrete these elements, and essential elements decreasing with age due to pregnancy (prediction 2). Some of the element-tissue combinations that turned out significant were in line with our prediction. For example, Cd increased with age in kidney (Fig. 2b), which is in line with previous studies [41, 42].

We found higher Cd concentrations for older females (Fig. 2a). An increase of Cd in eyes with age has been found in human retina as result of smoking, increasing risk of macular degeneration [46]. Jamall & Roque [47] found that a daily ingestion of 50 ppm Cd results in detectable accumulation after seven weeks in the eye of rats, implying that Cd in the diet can result in Cd accumulation in the eyes.

We found that K and Mg decreased with age in brain (Fig. 2f + h). A decrease of K in brain with age has previously been found in humans [48]. It implies reduced brain function and lower responsiveness since both K and Mg are important for pulse transmission and oxygen levels in the brain [e.g. 49]. However, it remains unclear why female calves might be more variable in their brain K and Mg concentrations compared to yearling females and does. This larger variation might be caused by the limited amount does can transfer to their young in these mineral-poor environments, although we do not have information on family relation of the culled animals.

Se functions as an important antioxidant, protecting against As and Cd toxicity, cancer, and heart disease [50, 51]. Dietary Se has been shown to affect the gut microbial colonization [52, 53], which might be in alignment with our finding that Se in stomach increased with age (Fig. 2p). This increase might improve the uptake of other minerals when animals get older [51].

We found four tissues that differed in Zn concentration among the female age groups (Fig. 2r-u). First, Zn is essential for normal skeletal growth and bone homeostasis [54], and decreased with age (Fig. 2r). This might be because bone mineral density tends to decline with age [55], causing the bone Zn concentration in younger animals more variable and lower in does. Second, Zn plays a role in muscle regeneration due to its effects on muscle cell activation [56]. This might be most needed in young animals, although it remains unclear why we found higher Zn concentrations in muscle for yearling females compared to calves and does (Fig. 2s). Third, Zn plays an integral role in maintaining a normal ocular function [57]. This might be more needed for older animals to slow down age-related macular degeneration [58], resulting in increasing Zn concentrations in eyes (Fig. 2t). Last, Zn plays an important role in the production of digestive enzymes [59]. However, it remains unclear why we found decreasing Zn concentrations with age (Fig. 2u).

Some of the other significant differences in element-tissue combinations were found in tissues where these elements play an important role. For example, the spleen has been mentioned to store the major Fe pool (Fig. 2e) [60]. Ni in the lungs is associated with an increased risk of lung cancer (Fig. 2l) [61], and S is a constituent of bones and collagen, associated with an increased risk of osteoporosis (Fig. 2n) [62]. However, we were unable to interpret their potential relationship with age.

### Age-related Differences in Males

We predicted age-related differences among males to be more related to bioaccumulation of toxic elements than differences in essential elements (prediction 3). However, the only toxic element that turned out significant was Al in the kidney (Fig. 3a). Kidney’s Al concentration was more variable, and for some much higher, for yearling males compared to calves, which was in alignment with previous studies [e.g. 63]. Free Al concentrations in the environment increase with decreasing pH due to anthropogenic acidification [64], which might be related to this finding.

We found higher Co concentrations and lower Mn concentrations in the eyes of yearling males compared to calves (Fig. 3b + h). The Co concentration in eyes is associated with age-related macular degeneration in humans [65], while senile cataractous has been associated with lower Mn levels in humans [66]. However, it remains unknown whether this also applies to animals, specifically deer.

Fe in muscles is important for many metabolic functions and electron transfer during energy production [e.g. 67–68]. DeRuisseau et al. [69] reported that, in humans, the total concentration of Fe in muscles increases during growth but stabilizes in senescence, which might be in line with our finding that yearling males have higher Fe concentrations in muscle compared to calves (Fig. 3e).

We found higher Fe concentrations in pancreas for yearling males than for calves (Fig. 3f). Fe in pancreas is associated with correct insulin synthesis and processing [70, 71]. Increased Fe levels in pancreas have been associated with an increased risk of pancreatic cancer [72], although this might not be relevant for wildlife. However, it seems unlikely that the animals in our study area, which is mineral poor, experienced an Fe overload that could lead to this difference in Fe concentration, especially when the acidification of this area is taken into account which set more Fe (and Al) free [73].

Male calves had higher Si concentrations compared to yearling males (Fig. 3p). Si is needed for muscle building, and is found to decrease with age in rats [74]. However, it remains unclear whether this also applies to deer or is relevant for wildlife.

We found higher Sr concentrations in bone and kidney for yearling males compared to calves (Fig. 3q + r). Sr is considered as the chemical analog of Ca and has a major role in the formation and breakdown of bones and preventing against osteoporosis [75–78]. Sr overload has been associated with renal dysfunction [79, 80]. However, Sr toxicity seems very unlikely in our nutrient-poor study area.

Contrary to the higher Zn concentration in muscle of yearling females compared to female calves (Fig. 2s), we found lower Zn concentrations in muscle for yearling males compared to male calves (Fig. 3s). This might indicate that this difference is found by chance rather than driven by age or sex.

### Sex-related Differences in Calves

As predicted, we indeed found the least significant tissue-element combinations when comparing sex-related differences among calves (prediction 4). However, we were unable to put most of our findings into the context of their biological and physiological role based on sex-related differences.

Female calves were more variable in their Al concentration in kidney and As concentration in muscle (Fig. 4a-b). We expected accumulation of these elements to increase with age since Al is not transferred via milk [81] and thus should be taken up through the environment.

Higher Na concentrations in bones, as we found for female calves (Fig. 4j), are associated with increased chance of osteoporosis at later age [82]. Although females tend to have higher risks of osteoporosis in general [83], it is unclear whether this might be associated with our finding.

We found higher Sr concentrations in the heart for females (Fig. 4p + Fig. 5w). Sr can protect the heart against heart infarct [84], although any sex-related differences remain unexplained, and it remains unknown whether this is applicable to deer as well.

Zn concentrations in the lungs and stomach were higher for females than for males (Fig. 4q-r). Zn has anti-inflammatory, antioxidant and antiviral effects in lungs [85]. It is also important for the production of digestive enzymes [86]. However, it is unknown how this might be associated with sex.

### Sex-related Differences in Yearlings

We found most sex-related differences in element-tissue combinations when comparing the female and male yearlings (prediction 5). As expected, we found that for most of these element-tissue combinations, males had higher concentrations than females (14 and 11, respectively; Fig. 5a-y). We were, however, unable to judge whether pregnancy was a major cause of these differences.

We found higher B concentrations in the liver of female yearlings compared to males (Fig. 5b). Liver is the first tissue that will be affected by overexposure to B [87, 88]. However, this seems unlikely due to our nutrient-poor study area. Any sex-specific causes remain unknown.

For Co, we found higher concentrations in the eyes for males (Fig. 5c) and in the liver of females (Fig. 5d). Regarding the liver, it has been shown that supplemental Co did not increase the Co storage in the liver [89]. This suggests that liver might not be the main target tissue for Co. Any sex-related differences in Co target tissues remain unknown.

We found higher Cr concentrations in the liver for females than for males (Fig. 5e). This contradicts previous findings in ducks [90] and horses [91], where males had higher Cr liver concentrations than females.

We found higher Cu concentrations in the brain of females (Fig. 5f) and in the heart of males (Fig. 5g). Quinn et al. [92] also found higher Cu concentrations in the brain of female rats and humans. This may be due to Cu-mediated pathological events in the brain. Besides, Cu deficiency might be a cause of ischaemic heart disease [93] - of which symptoms are sex-specific [94, 95] -, although it is unclear whether this is directly related to Cu levels in heart tissue.

For Na, we found higher concentrations in the eyes, heart and liver of males (Fig. 5p-r). Regarding the heart, Na in heart is associated with blood pressure [96], which is generally higher for males compared to females [97].

We found higher S concentrations in the intestines of males than for females (Fig. 5t). S amino acid metabolism is important for gut health [98]. It has been suggested that sex-related differences could be due to sex-related differences of the gut microbiota [99, 100], although we could only speculate whether this would result in higher S concentrations in males.

We found a higher Se concentration in the bones of males (Fig. 5u) and in the spleen of females (Fig. 5v). Se is crucial for bone development and bone mineral density maintenance [101, 102]. Also, both low and high Se concentrations can have negative effects on the immune function of the spleen (Zhang et al. 2022). However, although there are sex-related differences in Se metabolism [103], it is unclear how the differences that we found could be explained.

### Limitations

A strength of this study was the use of individuals of a single population that roamed the same protected area and that were culled in the same hunting season. However, we also see three major limitations. First, we did not include bucks in our analysis since no bucks were culled in the area. Second, we used a small sample size, with four individuals in each group. Therefore, we cannot rule out that some differences that we found were due to outliers. Third, in mineral-poor areas such as our study area, certain elements are scarce and not distributed uniformly across the landscape. Thus, we cannot judge but only speculate whether some variation in elemental concentrations arose from differences between individuals in where they foraged, as differences in habitat and diet selection across age and sex classes of Fallow deer may occur [104]. These limitations, however, do not invalidate our comparisons.

## Conclusion and Recommendations

We speculated on the biological and physiological role of chemical elements, focusing on age- and sex-related differences. In general, sex-related differences were more difficult to explain. This suggests that the current knowledge on chemical element allocation and metabolism in the body seems to be biased towards age-related patterns.

We found some ionomic differences between age and sex classes, as hypothesized, but the drivers of this variation remain unknown. Previous studies have suggested that diet might drive both sex-related [90] and age-related [105] variation within species. Life-history traits and ontogeny are also mentioned as causes of ionomic variation [106]. A number of decreasing elemental concentrations with age might refer to a decreased health condition of Fallow deer in this nutrient-poor environment (e.g. lower K in brains, lower Zn in muscles), although this is highly speculative. However, extensive studies in various species - including a wide range of elements and tissues - are missing to further explore these potential causes of ionomic variation. Therefore, we highly encourage the execution of such extensive ionomic surveys.

More extensive ionomic surveys are also needed to expand our understanding of physiological pathways underlying elemental allocation and metabolism. The limited available literature about the physiological role of elements in the body did not allow us to focus on Fallow deer only, accordingly we were not able to infer what the higher or lower values that we found would mean when observed in other species or humans. Moreover, reference values, that are needed to judge about any potential toxicities and deficiencies, are absent. Signs of toxicities and deficiencies are element-, or even species-, specific [107–109]. Thus, we cannot judge about the consequences of the elemental values that we found without such reference values.

The lack of reference values, and the severe elemental variation within and among age and sex groups as we report in this paper, limit the use of wild-living animals for biomonitoring purposes, which is usually based on a limited number of elements in only a few tissues [e.g. 17, 32, 110]. We believe that it is important to increase our understanding of this variation in more detail before this can be applied to biomonitoring purposes. It would be worthwhile to repeat our study in different areas, that differ in their mineral availability. Additionally, addressing ionomic variation in age and sex for other species would be valuable in order to investigate the general applicability of our study. Our study should thus been considered as a first step towards a more comprehensive understanding of the mammalian ionome.

In conclusion, we found that ionomic variation in a wide range of chemical elements and tissues within a single population was indeed partially related to age and sex. The limited existing knowledge about element-specific metabolic pathways and causes of ionomic variation did not allow us to put all these differences in a biological or physiological context. We encourage other scientists to conduct more extensive surveys of the ionome of different species, based on a wide range of elements and tissues, to enlarge our understanding of the mammalian ionome and potential biological, ecological, and metabolic consequences.

### Electronic Supplementary Material

Below is the link to the electronic supplementary material.

Supplementary Material 1

Supplementary Material 2

Supplementary Material 3

## Data Availability

The data generated and analyzed during this study is available via Figshare: 10.6084/m9.figshare.22140368.
