# Supplementary material for: Ionomic Variation Among Tissues in Fallow Deer (Dama dama) by Sex and Age
Source: Biol Trace Elem Res. 2023 Jun 8;202(3):965–79. doi: 10.1007/s12011-023-03724-x (PMC10803548; doi:10.1007/s12011-023-03724-x)
Supplement: Supplementary file 1 — Supplementary Material 1 [file 12011_2023_3724_MOESM1_ESM.pdf]

Memo

Corporate Education and Research

DATE
02/03/2023

POSTAL ADDRESS
Bode 120
The Netherlands

VISITORS' ADDRESS
Bornse Weilanden 5
6708 WG Wageningen

INTERNET
www.wageningenuniversity.nl

HANDLED BY

TELEPHONE

EMAIL

TO
Elke Wenting

FROM

Janneke Arts, Animal Welfare Officer

Dear Elke,

The Animal Welfare Body assessed the work protocol**: "Ionomic variation among tissues in Fallow deer (Dama dama) by sex and age"**, received on **02/03/2023**.

It is the opinion of the AWO that this is not an animal experiment as referred to in the Dutch Act on Animal Experiments, since the experimental procedures described in present protocol will not make use of live animals or animals that were killed for the purpose of tissue sampling.

Your application has been registered by IvD-WU with number **NAE_2023.W-010**

In any unforeseen circumstances, or intended deviations from the aforementioned protocol, please contact the animal welfare officer.

Yours sincerely,
Dr. Ir. Ing. J.W.M. Arts

Animal Welfare Officer

Wageningen UR (Wageningen University and various research institutes) is specialised in the domain of healthy food and living environment.
