# Supplementary material for: Ionomic Variation Among Tissues in Fallow Deer (Dama dama) by Sex and Age
Source: Biol Trace Elem Res. 2023 Jun 8;202(3):965–79. doi: 10.1007/s12011-023-03724-x (PMC10803548; doi:10.1007/s12011-023-03724-x)
Supplement: Supplementary file 2 — Supplementary Material 2 [file 12011_2023_3724_MOESM2_ESM.docx]

**Test statistics of Kruskal-Wallis tests (alpha of 0.00227 considered as significant after Bonferroni correction) for the total elemental concentrations.**

| Element | Test-statistics |
| --- | --- |
| Al | X^2^ = 5.10, df = 4, p = 0.277 |
| As | X^2^ = 1.81, df = 4, p = 0.770 |
| B | X^2^ = 8.17, df = 4, p = 0.086 |
| Ca | X^2^ = 4.46, df = 4, p = 0.348 |
| Cd | X^2^ = 11.64, df = 4, p = 0.020 |
| Co | X^2^ = 2.04, df = 4, p = 0.728 |
| Cr | X^2^ = 5.04, df = 4, p = 0.283 |
| Cu | X^2^ = 10.53, df = 4, p = 0.032 |
| Fe | X^2^ = 4.17, df = 4, p = 0.383 |
| K | X^2^ = 3.37, df = 4, p = 0.498 |
| Mg | X^2^ = 5.39, df = 4, p = 0.250 |
| Mn | X^2^ = 3.41, df = 4, p = 0.491 |
| Mo | X^2^ = 2.60, df = 4, p = 0.627 |
| Na | X^2^ = 9.57, df = 4, p = 0.048 |
| Ni | X^2^ = 2.64, df = 4, p = 0.619 |
| P | X^2^ = 4.31, df = 4, p = 0.365 |
| Pb | X^2^ = 4.81, df = 4, p = 0.307 |
| S | X^2^ = 2.386, df = 4, p = 0.665 |
| Se | X^2^ = 3.39, df = 4, p = 0.500 |
| Si | X^2^ = 0.93, df = 4, p = 0.920 |
| Sr | X^2^ = 3.33, df = 4, p = 0.504 |
| Zn | X^2^ = 5.44, df = 4, p = 0.245 |

**Test statistics of Kruskal Wallis tests for element-tissue combinations for age classes in female Fallow deer (adjusted p-values are reported after correction using the** **Benjamini and Hochberg procedure). * indicates significance.**

| Al  Tissue | Test-statistics |
| --- | --- |
| Bone | X^2^ = 4.77, df = 2, p = 0.062 |
| Skin & hair | X^2^ = 2.61, df = 2, p = 0.139 |
| Muscle | X^2^ = 0.07, df = 2, p = 0.436 |
| Brain | X^2^ = NaN, df = 2, p = NaN |
| Eyes | X^2^ = 2.85, df = 2, p = 0.130 |
| Lungs | X^2^ = 1.00, df = 2, p = 0.305 |
| Heart | X^2^ = 1.34, df = 2, p = 0.246 |
| Spleen | X^2^ = 1.11, df = 2, p = 0.277 |
| Kidney | X^2^ = 1.98, df = 2, p = 0.181 |
| Liver | X^2^ = 2.83, df = 2, p = 0.131 |
| Pancreas | X^2^ = 1.11, df = 2, p = 0.277 |
| Stomach | X^2^ = 1.65, df = 2, p = 0.217 |
| Intestines | X^2^ = 0.81, df = 2, p = 0.317 |
| As  Tissue | Test-statistics |
| Bone | X^2^ = 0.15, df = 2, p = 0.421 |
| Skin & hair | X^2^ = 1.42, df = 2, p = 0.238 |
| Muscle | X^2^ = 0.35, df = 2, p = 0.387 |
| Brain | X^2^ = 2.20, df = 2, p = 0.167 |
| Eyes | X^2^ = 4.31, df = 2, p = 0.072 |
| Lungs | X^2^ = 0.81, df = 2, p = 0.328 |
| Heart | X^2^ = 0.15, df = 2, p = 0.421 |
| Spleen | X^2^ = 0.35, df = 2, p = 0.387 |
| Kidney | X^2^ = 2.00, df = 2, p = 0.180 |
| Liver | X^2^ = 3.50, df = 2, p = 0.105 |
| Pancreas | X^2^ = 0.27, df = 2, p = 0.405 |
| Stomach | X^2^ = 1.19, df = 2, p = 0.271 |
| Intestines | X^2^ = 0.27, df = 2, p = 0.405 |

| B  Tissue | Test-statistics |
| --- | --- |
| Bone | X^2^ = 1.08, df = 2, p = 0.296 |
| Skin & hair | X^2^ = 1.85, df = 2, p = 0.210 |
| Muscle | X^2^ = 1.85, df = 2, p = 0.210 |
| Brain | X^2^ = 0.81, df = 2, p = 0.328 |
| Eyes | X^2^ = 1.42, df = 2, p = 0.238 |
| Lungs | X^2^ = 0.73, df = 2, p = 0.344 |
| Heart | X^2^ = 1.88, df = 2, p = 0.204 |
| Spleen | X^2^ = 3.11, df = 2, p = 0.114 |
| Kidney | X^2^ = 1.19, df = 2, p = 0.271 |
| Liver | X^2^ = 3.50, df = 2, p = 0.105 |
| Pancreas | X^2^ = 3.50, df = 2, p = 0.105 |
| Stomach | X^2^ = 1.88, df = 2, p = 0.204 |
| Intestines | X^2^ = 4.27, df = 2, p = 0.074 |

| Ca  Tissue | Test-statistics |
| --- | --- |
| Bone | X^2^ = 2.00, df = 2, p = 0.180 |
| Skin & hair | X^2^ = 0.15, df = 2, p = 0.421 |
| Muscle | X^2^ = 0.27, df = 2, p = 0.405 |
| Brain | X^2^ = 2.92, df = 2, p = 0.126 |
| Eyes | X^2^ = 1.38, df = 2, p = 0.244 |
| Lungs | X^2^ = 0.50, df = 2, p = 0.367 |
| Heart | X^2^ = 0.27, df = 2, p = 0.405 |
| Spleen | X^2^ = 0.81, df = 2, p = 0.328 |
| Kidney | X^2^ = 3.04, df = 2, p = 0.120 |
| Liver | X^2^ = 0.00, df = 2, p = 0.449 |
| Pancreas | X^2^ = 3.85, df = 2, p = 0.088 |
| Stomach | X^2^ = 0.15, df = 2, p = 0.421 |
| Intestines | X^2^ = 0.46, df = 2, p = 0.377 |

| Cd  Tissue | Test-statistics |
| --- | --- |
| Bone | X^2^ = 1.08, df = 2, p = 0.296 |
| Skin & hair | X^2^ = 1.09, df = 2, p = 0.281 |
| Muscle | X^2^ = 0.74, df = 2, p = 0.333 |
| Brain | X^2^ = 4.77, df = 2, p = 0.062 |
| Eyes | X^2^ = 7.54, df = 2, p = 0.017 * |
| Lungs | X^2^ = 2.88, df = 2, p = 0.128 |
| Heart | X^2^ = 0.51, df = 2, p = 0.351 |
| Spleen | X^2^ = 0.62, df = 2, p = 0.349 |
| Kidney | X^2^ = 7.42, df = 2, p = 0.019 * |
| Liver | X^2^ = 3.50, df = 2, p = 0.105 |
| Pancreas | X^2^ = 3.58, df = 2, p = 0.096 |
| Stomach | X^2^ = 1.88, df = 2, p = 0.204 |
| Intestines | X^2^ = 1.19, df = 2, p = 0.271 |

| Co  Tissue | Test-statistics |
| --- | --- |
| Bone | X^2^ = 0.07, df = 2, p = 0.436 |
| Skin & hair | X^2^ = 1.21, df = 2, p = 0.256 |
| Muscle | X^2^ = 1.87, df = 2, p = 0.205 |
| Brain | X^2^ = 2.11, df = 2, p = 0.175 |
| Eyes | X^2^ = 0.91, df = 2, p = 0.314 |
| Lungs | X^2^ = 5.75, df = 2, p = 0.033 * |
| Heart | X^2^ = 1.28, df = 2, p = 0.249 |
| Spleen | X^2^ = 0.27, df = 2, p = 0.388 |
| Kidney | X^2^ = 2.19, df = 2, p = 0.174 |
| Liver | X^2^ = 0.27, df = 2, p = 0.405 |
| Pancreas | X^2^ = 0.16, df = 2, p = 0.407 |
| Stomach | X^2^ = 1.88, df = 2, p = 0.204 |
| Intestines | X^2^ = 0.94, df = 2, p = 0.312 |

| Cr  Tissue | Test-statistics |
| --- | --- |
| Bone | X^2^ = 0.87, df = 2, p = 0.315 |
| Skin & hair | X^2^ = 1.19, df = 2, p = 0.271 |
| Muscle | X^2^ = 3.85, df = 2, p = 0.086 |
| Brain | X^2^ = 1.53, df = 2, p = 0.218 |
| Eyes | X^2^ = 0.04, df = 2, p = 0.442 |
| Lungs | X^2^ = 4.19, df = 2, p = 0.079 |
| Heart | X^2^ = 0.27, df = 2, p = 0.405 |
| Spleen | X^2^ = 1.85, df = 2, p = 0.210 |
| Kidney | X^2^ = 3.73, df = 2, p = 0.089 |
| Liver | X^2^ = 3.23, df = 2, p = 0.110 |
| Pancreas | X^2^ = 1.09, df = 2, p = 0.281 |
| Stomach | X^2^ = 0.15, df = 2, p = 0.421 |
| Intestines | X^2^ = 0.50, df = 2, p = 0.367 |

| Cu  Tissue | Test-statistics |
| --- | --- |
| Bone | X^2^ = 2.39, df = 2, p = 0.161 |
| Skin & hair | X^2^ = 3.50, df = 2, p = 0.105 |
| Muscle | X^2^ = 2.42, df = 2, p = 0.158 |
| Brain | X^2^ = 0.50, df = 2, p = 0.367 |
| Eyes | X^2^ = 2.00, df = 2, p = 0.180 |
| Lungs | X^2^ = 2.58, df = 2, p = 0.143 |
| Heart | X^2^ = 0.96, df = 2, p = 0.311 |
| Spleen | X^2^ = 2.88, df = 2, p = 0.128 |
| Kidney | X^2^ = 0.35, df = 2, p = 0.387 |
| Liver | X^2^ = 4.19, df = 2, p = 0.079 |
| Pancreas | X^2^ = 3.96, df = 2, p = 0.085 |
| Stomach | X^2^ = 4.88, df = 2, p = 0.053 |
| Intestines | X^2^ = 0.27, df = 2, p = 0.405 |

| Fe  Tissue | Test-statistics |
| --- | --- |
| Bone | X^2^ = 2.39, df = 2, p = 0.277 |
| Skin & hair | X^2^ = 3.50, df = 2, p = 0.161 |
| Muscle | X^2^ = 2.42, df = 2, p = 0.296 |
| Brain | X^2^ = 0.50, df = 2, p = 0.105 |
| Eyes | X^2^ = 2.00, df = 2, p = 0.024 * |
| Lungs | X^2^ = 2.58, df = 2, p = 0.204 |
| Heart | X^2^ = 0.96, df = 2, p = 0.421 |
| Spleen | X^2^ = 2.88, df = 2, p = 0.037 * |
| Kidney | X^2^ = 0.35, df = 2, p = 0.367 |
| Liver | X^2^ = 4.19, df = 2, p = 0.238 |
| Pancreas | X^2^ = 3.96, df = 2, p = 0.126 |
| Stomach | X^2^ = 4.88, df = 2, p = 0.062 |
| Intestines | X^2^ = 0.27, df = 2, p = 0.256 |

| K  Tissue | Test-statistics |
| --- | --- |
| Bone | X^2^ = 1.50, df = 2, p = 0.227 |
| Skin & hair | X^2^ = 0.50, df = 2, p = 0.367 |
| Muscle | X^2^ = 1.19, df = 2, p = 0.271 |
| Brain | X^2^ = 8.00, df = 2, p = 0.014 * |
| Eyes | X^2^ = 2.35, df = 2, p = 0.164 |
| Lungs | X^2^ = 2.42, df = 2, p = 0.158 |
| Heart | X^2^ = 1.19, df = 2, p = 0.271 |
| Spleen | X^2^ = 3.96, df = 2, p = 0.085 |
| Kidney | X^2^ = 1.38, df = 2, p = 0.244 |
| Liver | X^2^ = 3.12, df = 2, p = 0.114 |
| Pancreas | X^2^ = 2.42, df = 2, p = 0.158 |
| Stomach | X^2^ = 5.58, df = 2, p = 0.096 |
| Intestines | X^2^ = 1.38, df = 2, p = 0.244 |

| Mg  Tissue | Test-statistics |
| --- | --- |
| Bone | X^2^ = 9.27, df = 2, p = 0.010 * |
| Skin & hair | X^2^ = 0.00, df = 2, p = 0.449 |
| Muscle | X^2^ = 2.19, df = 2, p = 0.174 |
| Brain | X^2^ = 6.73, df = 2, p = 0.022 * |
| Eyes | X^2^ = 1.50, df = 2, p = 0.227 |
| Lungs | X^2^ = 1.88, df = 2, p = 0.204 |
| Heart | X^2^ = 0.50, df = 2, p = 0.367 |
| Spleen | X^2^ = 4.19, df = 2, p = 0.079 |
| Kidney | X^2^ = 2.42, df = 2, p = 0.158 |
| Liver | X^2^ = 0.50, df = 2, p = 0.367 |
| Pancreas | X^2^ = 3.96, df = 2, p = 0.085 |
| Stomach | X^2^ = 0.73, df = 2, p = 0.344 |
| Intestines | X^2^ = 1.50, df = 2, p = 0.227 |

| Mn  Tissue | Test-statistics |
| --- | --- |
| Bone | X^2^ = 1.08, df = 2, p = 0.296 |
| Skin & hair | X^2^ = 1.04, df = 2, p = 0.303 |
| Muscle | X^2^ = 0.73, df = 2, p = 0.344 |
| Brain | X^2^ = 3.58, df = 2, p = 0.096 |
| Eyes | X^2^ = 2.92, df = 2, p = 0.126 |
| Lungs | X^2^ = 2.42, df = 2, p = 0.158 |
| Heart | X^2^ = 1.04, df = 2, p = 0.303 |
| Spleen | X^2^ = 0.12, df = 2, p = 0.432 |
| Kidney | X^2^ = 5.12, df = 2, p = 0.045 * |
| Liver | X^2^ = 6.50, df = 2, p = 0.029 * |
| Pancreas | X^2^ = 3.50, df = 2, p = 0.105 |
| Stomach | X^2^ = 1.88, df = 2, p = 0.204 |
| Intestines | X^2^ = 0.46, df = 2, p = 0.377 |

| Mo  Tissue | Test-statistics |
| --- | --- |
| Bone | X^2^ = 4.77, df = 2, p = 0.062 |
| Skin & hair | X^2^ = 2.19, df = 2, p = 0.174 |
| Muscle | X^2^ = 1.42, df = 2, p = 0.238 |
| Brain | X^2^ = 1.88, df = 2, p = 0.204 |
| Eyes | X^2^ = 6.62, df = 2, p = 0.027 * |
| Lungs | X^2^ = 1.42, df = 2, p = 0.238 |
| Heart | X^2^ = 0.46, df = 2, p = 0.377 |
| Spleen | X^2^ = 3.04, df = 2, p = 0.120 |
| Kidney | X^2^ = 0.12, df = 2, p = 0.432 |
| Liver | X^2^ = 0.81, df = 2, p = 0.328 |
| Pancreas | X^2^ = 2.46, df = 2, p = 0.147 |
| Stomach | X^2^ = 2.81, df = 2, p = 0.136 |
| Intestines | X^2^ = 3.23, df = 2, p = 0.110 |

| Na  Tissue | Test-statistics |
| --- | --- |
| Bone | X^2^ = 0.81, df = 2, p = 0.328 |
| Skin & hair | X^2^ = 0.00, df = 2, p = 0.449 |
| Muscle | X^2^ = 4.50, df = 2, p = 0.067 |
| Brain | X^2^ = 3.58, df = 2, p = 0.096 |
| Eyes | X^2^ = 2.35, df = 2, p = 0.164 |
| Lungs | X^2^ = 0.46, df = 2, p = 0.377 |
| Heart | X^2^ = 2.46, df = 2, p = 0.147 |
| Spleen | X^2^ = 0.50, df = 2, p = 0.367 |
| Kidney | X^2^ = 1.42, df = 2, p = 0.238 |
| Liver | X^2^ = 0.27, df = 2, p = 0.405 |
| Pancreas | X^2^ = 4.50, df = 2, p = 0.067 |
| Stomach | X^2^ = 3.50, df = 2, p = 0.105 |
| Intestines | X^2^ = 1.08, df = 2, p = 0.296 |

| Ni  Tissue | Test-statistics |
| --- | --- |
| Bone | X^2^ = 0.12, df = 2, p = 0.423 |
| Skin & hair | X^2^ = 1.07, df = 2, p = 0.297 |
| Muscle | X^2^ = 3.47, df = 2, p = 0.106 |
| Brain | X^2^ = 2.76, df = 2, p = 0.137 |
| Eyes | X^2^ = 0.50, df = 2, p = 0.367 |
| Lungs | X^2^ = 7.38, df = 2, p = 0.020 * |
| Heart | X^2^ = 1.28, df = 2, p = 0.249 |
| Spleen | X^2^ = 2.29, df = 2, p = 0.165 |
| Kidney | X^2^ = 3.85, df = 2, p = 0.088 |
| Liver | X^2^ = 0.62, df = 2, p = 0.346 |
| Pancreas | X^2^ = 0.73, df = 2, p = 0.344 |
| Stomach | X^2^ = 2.46, df = 2, p = 0.147 |
| Intestines | X^2^ = 1.88, df = 2, p = 0.204 |

| P  Tissue | Test-statistics |
| --- | --- |
| Bone | X^2^ = 4.15, df = 2, p = 0.080 |
| Skin & hair | X^2^ = 1.65, df = 2, p = 0.217 |
| Muscle | X^2^ = 1.65, df = 2, p = 0.217 |
| Brain | X^2^ = 0.12, df = 2, p = 0.432 |
| Eyes | X^2^ = 1.85, df = 2, p = 0.210 |
| Lungs | X^2^ = 2.19, df = 2, p = 0.174 |
| Heart | X^2^ = 0.73, df = 2, p = 0.344 |
| Spleen | X^2^ = 5.65, df = 2, p = 0.037 * |
| Kidney | X^2^ = 4.65, df = 2, p = 0.064 |
| Liver | X^2^ = 2.81, df = 2, p = 0.136 |
| Pancreas | X^2^ = 4.77, df = 2, p = 0.062 |
| Stomach | X^2^ = 2.81, df = 2, p = 0.136 |
| Intestines | X^2^ = 2.42, df = 2, p = 0.158 |

| Pb  Tissue | Test-statistics |
| --- | --- |
| Bone | X^2^ = 4.27, df = 2, p = 0.074 |
| Skin & hair | X^2^ = 0.81, df = 2, p = 0.328 |
| Muscle | X^2^ = 1.16, df = 2, p = 0.272 |
| Brain | X^2^ = 4.26, df = 2, p = 0.075 |
| Eyes | X^2^ = 0.79, df = 2, p = 0.330 |
| Lungs | X^2^ = 1.04, df = 2, p = 0.303 |
| Heart | X^2^ = 0.43, df = 2, p = 0.379 |
| Spleen | X^2^ = 0.12, df = 2, p = 0.432 |
| Kidney | X^2^ = 1.65, df = 2, p = 0.217 |
| Liver | X^2^ = 3.04, df = 2, p = 0.120 |
| Pancreas | X^2^ = 1.24, df = 2, p = 0.251 |
| Stomach | X^2^ = 3.23, df = 2, p = 0.110 |
| Intestines | X^2^ = 1.50, df = 2, p = 0.227 |

| S  Tissue | Test-statistics |
| --- | --- |
| Bone | X^2^ = 5.12, df = 2, p = 0.045 * |
| Skin & hair | X^2^ = 0.04, df = 2, p = 0.442 |
| Muscle | X^2^ = 2.58, df = 2, p = 0.143 |
| Brain | X^2^ = 1.88, df = 2, p = 0.204 |
| Eyes | X^2^ = 3.96, df = 2, p = 0.085 |
| Lungs | X^2^ = 2.42, df = 2, p = 0.158 |
| Heart | X^2^ = 1.38, df = 2, p = 0.244 |
| Spleen | X^2^ = 0.35, df = 2, p = 0.387 |
| Kidney | X^2^ = 3.04, df = 2, p = 0.120 |
| Liver | X^2^ = 5.12, df = 2, p = 0.045 * |
| Pancreas | X^2^ = 1.88, df = 2, p = 0.204 |
| Stomach | X^2^ = 1.08, df = 2, p = 0.296 |
| Intestines | X^2^ = 2.19, df = 2, p = 0.174 |

| Se  Tissue | Test-statistics |
| --- | --- |
| Bone | X^2^ = 1.88, df = 2, p = 0.204 |
| Skin & hair | X^2^ = 0.46, df = 2, p = 0.377 |
| Muscle | X^2^ = 1.19, df = 2, p = 0.271 |
| Brain | X^2^ = 4.31, df = 2, p = 0.072 |
| Eyes | X^2^ = 2.92, df = 2, p = 0.126 |
| Lungs | X^2^ = 4.31, df = 2, p = 0.072 |
| Heart | X^2^ = 4.19, df = 2, p = 0.079 |
| Spleen | X^2^ = 3.04, df = 2, p = 0.120 |
| Kidney | X^2^ = 2.81, df = 2, p = 0.136 |
| Liver | X^2^ = 1.88, df = 2, p = 0.204 |
| Pancreas | X^2^ = 4.31, df = 2, p = 0.072 |
| Stomach | X^2^ = 4.96, df = 2, p = 0.049 * |
| Intestines | X^2^ = 1.88, df = 2, p = 0.204 |

| Si  Tissue | Test-statistics |
| --- | --- |
| Bone | X^2^ = 5.11, df = 2, p = 0.047 * |
| Skin & hair | X^2^ = 1.19, df = 2, p = 0.271 |
| Muscle | X^2^ = 0.04, df = 2, p = 0.442 |
| Brain | X^2^ = 3.13, df = 2, p = 0.111 |
| Eyes | X^2^ = 1.51, df = 2, p = 0.220 |
| Lungs | X^2^ = 2.58, df = 2, p = 0.143 |
| Heart | X^2^ = 1.19, df = 2, p = 0.271 |
| Spleen | X^2^ = 1.08, df = 2, p = 0.296 |
| Kidney | X^2^ = 4.65, df = 2, p = 0.064 |
| Liver | X^2^ = 0.15, df = 2, p = 0.421 |
| Pancreas | X^2^ = 0.46, df = 2, p = 0.377 |
| Stomach | X^2^ = 2.42, df = 2, p = 0.158 |
| Intestines | X^2^ = 0.27, df = 2, p = 0.405 |

| Sr  Tissue | Test-statistics |
| --- | --- |
| Bone | X^2^ = 4.77, df = 2, p = 0.062 |
| Skin & hair | X^2^ = 0.73, df = 2, p = 0.344 |
| Muscle | X^2^ = 0.79, df = 2, p = 0.332 |
| Brain | X^2^ = 1.08, df = 2, p = 0.296 |
| Eyes | X^2^ = 1.08, df = 2, p = 0.296 |
| Lungs | X^2^ = 0.96, df = 2, p = 0.311 |
| Heart | X^2^ = 4.84, df = 2, p = 0.065 |
| Spleen | X^2^ = 0.96, df = 2, p = 0.311 |
| Kidney | X^2^ = 0.62, df = 2, p = 0.349 |
| Liver | X^2^ = 1.21, df = 2, p = 0.256 |
| Pancreas | X^2^ = 4.88, df = 2, p = 0.053 |
| Stomach | X^2^ = 0.96, df = 2, p = 0.311 |
| Intestines | X^2^ = 1.88, df = 2, p = 0.204 |

| Zn  Tissue | Test-statistics |
| --- | --- |
| Bone | X^2^ = 5.12, df = 2, p = 0.045 * |
| Skin & hair | X^2^ = 0.12, df = 2, p = 0.432 |
| Muscle | X^2^ = 6.50, df = 2, p = 0.029 * |
| Brain | X^2^ = 1.08, df = 2, p = 0.296 |
| Eyes | X^2^ = 5.81, df = 2, p = 0.031 * |
| Lungs | X^2^ = 3.58, df = 2, p = 0.096 |
| Heart | X^2^ = 0.81, df = 2, p = 0.328 |
| Spleen | X^2^ = 1.04, df = 2, p = 0.303 |
| Kidney | X^2^ = 0.73, df = 2, p = 0.344 |
| Liver | X^2^ = 1.42, df = 2, p = 0.238 |
| Pancreas | X^2^ = 4.88, df = 2, p = 0.053 |
| Stomach | X^2^ = 6.62, df = 2, p = 0.027 * |
| Intestines | X^2^ = 0.00, df = 2, p = 0.449 |

**Test statistics of Mann-Whitney U tests for element-tissue combinations for age classes in male Fallow deer (adjusted p-values are reported after correction using the** **Benjamini and Hochberg procedure). * indicates significance.**

| Al  Tissue | Test-statistics |
| --- | --- |
| Bone | W = 10, p = 0.405 |
| Skin & hair | W = 7.5, p = 0.584 |
| Muscle | W = 4, p = 0.077 |
| Brain | W = NaN, p = NaN |
| Eyes | W = NaN, p = NaN |
| Lungs | W = 4, p = 0.077 |
| Heart | W = 7, p = 0.422 |
| Spleen | W = 6, p = 0.222 |
| Kidney | W = 2, p = 0.040 * |
| Liver | W = 7.5, p = 0.584 |
| Pancreas | W = 10, p = 0.299 |
| Stomach | W = 12, p = 0.141 |
| Intestines | W = 12, p = 0.215 |

| As  Tissue | Test-statistics |
| --- | --- |
| Bone | W = 4, p = 0.215 |
| Skin & hair | W = 9, p = 0.527 |
| Muscle | W = 14, p = 0.070 |
| Brain | W = 7.5, p = 0.584 |
| Eyes | W = 5.5, p = 0.294 |
| Lungs | W = 9, p = 0.527 |
| Heart | W = 10, p = 0.405 |
| Spleen | W = 8, p = 0.584 |
| Kidney | W = 10.5, p = 0.294 |
| Liver | W = 12.5, p = 0.132 |
| Pancreas | W = 6, p = 0.405 |
| Stomach | W = 12, p = 0.215 |
| Intestines | W = 5, p = 0.288 |

| B  Tissue | Test-statistics |
| --- | --- |
| Bone | W = 5, p = 0.288 |
| Skin & hair | W = 7, p = 0.527 |
| Muscle | W = 8, p = 0.584 |
| Brain | W = 8, p = 0.584 |
| Eyes | W = 6, p = 0.405 |
| Lungs | W = 4, p = 0.215 |
| Heart | W = 11, p = 0.288 |
| Spleen | W = 11, p = 0.288 |
| Kidney | W = 3, p = 0.126 |
| Liver | W = 6, p = 0.405 |
| Pancreas | W = 6, p = 0.405 |
| Stomach | W = 12, p = 0.215 |
| Intestines | W = 2, p = 0.070 |
| Ca  Tissue | Test-statistics |
| Bone | W = 6, p = 0.405 |
| Skin & hair | W = 4, p = 0.215 |
| Muscle | W = 12, p = 0.215 |
| Brain | W = 9, p = 0.527 |
| Eyes | W = 7, p = 0.527 |
| Lungs | W = 7, p = 0.527 |
| Heart | W = 4, p = 0.215 |
| Spleen | W = 6, p = 0.405 |
| Kidney | W = 7, p = 0.527 |
| Liver | W = 2, p = 0.070 |
| Pancreas | W = 8, p = 0.584 |
| Stomach | W = 13, p = 0.126 |
| Intestines | W = 8, p = 0.584 |

| Cd  Tissue | Test-statistics |
| --- | --- |
| Bone | W = 6, p = 0.405 |
| Skin & hair | W = 11, p = 0.288 |
| Muscle | W = 9, p = 0.527 |
| Brain | W = 6, p = 0.405 |
| Eyes | W = 3, p = 0.126 |
| Lungs | W = 3, p = 0.126 |
| Heart | W = 8, p = 0.584 |
| Spleen | W = 10, p = 0.405 |
| Kidney | W = 2, p = 0.070 |
| Liver | W = 7, p = 0.527 |
| Pancreas | W = 3, p = 0.126 |
| Stomach | W = 11, p = 0.288 |
| Intestines | W = 4, p = 0.215 |

| Co  Tissue | Test-statistics |
| --- | --- |
| Bone | W = NaN, p = NaN |
| Skin & hair | W = 7, p = 0.415 |
| Muscle | W = 10, p = 0.299 |
| Brain | W = 5, p = 0.288 |
| Eyes | W = 0, p = 0.029 * |
| Lungs | W = 9, p = 0.415 |
| Heart | W = 8, p = 0.584 |
| Spleen | W = 15, p = 0.031 * |
| Kidney | W = 7, p = 0.527 |
| Liver | W = 11, p = 0.288 |
| Pancreas | W = 8, p = 0.584 |
| Stomach | W = 16, p = 0.029 * |
| Intestines | W = 12, p = 0.215 |

| Cr  Tissue | Test-statistics |
| --- | --- |
| Bone | W = 7.5, p = 0.584 |
| Skin & hair | W = 7, p = 0.527 |
| Muscle | W = 11, p = 0.229 |
| Brain | W = 10, p = 0.405 |
| Eyes | W = 3, p = 0.126 |
| Lungs | W = 10, p = 0.307 |
| Heart | W = 5, p = 0.229 |
| Spleen | W = 13, p = 0.126 |
| Kidney | W = 10, p = 0.405 |
| Liver | W = 11, p = 0.233 |
| Pancreas | W = 11, p = 0.229 |
| Stomach | W = 11, p = 0.288 |
| Intestines | W = 11, p = 0.288 |

| Cu  Tissue | Test-statistics |
| --- | --- |
| Bone | W = 12.5, p = 0.127 |
| Skin & hair | W = 11, p = 0.288 |
| Muscle | W = 6, p = 0.405 |
| Brain | W = 9, p = 0.527 |
| Eyes | W = 9, p = 0.527 |
| Lungs | W = 7, p = 0.527 |
| Heart | W = 3, p = 0.126 |
| Spleen | W = 4, p = 0.215 |
| Kidney | W = 6, p = 0.405 |
| Liver | W = 10, p = 0.405 |
| Pancreas | W = 7, p = 0.527 |
| Stomach | W = 9, p = 0.527 |
| Intestines | W = 5, p = 0.288 |

| Fe  Tissue | Test-statistics |
| --- | --- |
| Bone | W = NaN, p = NaN |
| Skin & hair | W = 6, p = 0.222 |
| Muscle | W = 0, p = 0.029 * |
| Brain | W = 4, p = 0.215 |
| Eyes | W = 8, p = 0.584 |
| Lungs | W = 9, p = 0.527 |
| Heart | W = 9, p = 0.527 |
| Spleen | W = 7, p = 0.527 |
| Kidney | W = 9, p = 0.527 |
| Liver | W = 5, p = 0.288 |
| Pancreas | W = 1, p = 0.039 * |
| Stomach | W = 11, p = 0.288 |
| Intestines | W = 3.5, p = 0.132 |

| K  Tissue | Test-statistics |
| --- | --- |
| Bone | W = 9, p = 0.527 |
| Skin & hair | W = 9, p = 0.527 |
| Muscle | W = 8, p = 0.584 |
| Brain | W = 11, p = 0.288 |
| Eyes | W = 7, p = 0.527 |
| Lungs | W = 4, p = 0.215 |
| Heart | W = 4, p = 0.215 |
| Spleen | W = 10, p = 0.405 |
| Kidney | W = 6, p = 0.405 |
| Liver | W = 15, p = 0.039 * |
| Pancreas | W = 6, p = 0.405 |
| Stomach | W = 11, p = 0.288 |
| Intestines | W = 3, p = 0.126 |

| Mg  Tissue | Test-statistics |
| --- | --- |
| Bone | W = 11, p = 0.288 |
| Skin & hair | W = 13, p = 0.126 |
| Muscle | W = 6, p = 0.405 |
| Brain | W = 7, p = 0.527 |
| Eyes | W = 8, p = 0.584 |
| Lungs | W = 4, p = 0.215 |
| Heart | W = 4, p = 0.215 |
| Spleen | W = 9, p = 0.527 |
| Kidney | W = 8, p = 0.584 |
| Liver | W = 12, p = 0.215 |
| Pancreas | W = 6, p = 0.405 |
| Stomach | W = 13, p = 0.126 |
| Intestines | W = 7, p = 0.527 |

| Mn  Tissue | Test-statistics |
| --- | --- |
| Bone | W = 8, p = 0.584 |
| Skin & hair | W = 3, p = 0.126 |
| Muscle | W = 5, p = 0.288 |
| Brain | W = 14, p = 0.070 |
| Eyes | W = 16, p = 0.029 * |
| Lungs | W = 7, p = 0.527 |
| Heart | W = 4, p = 0.215 |
| Spleen | W = 9, p = 0.527 |
| Kidney | W = 4, p = 0.215 |
| Liver | W = 4, p = 0.215 |
| Pancreas | W = 10, p = 0.405 |
| Stomach | W = 14, p = 0.070 |
| Intestines | W = 8, p = 0.584 |

| Mo  Tissue | Test-statistics |
| --- | --- |
| Bone | W = 4, p = 0.141 |
| Skin & hair | W = 10, p = 0.405 |
| Muscle | W = 7, p = 0.527 |
| Brain | W = 4, p = 0.215 |
| Eyes | W = 0, p = 0.029 * |
| Lungs | W = 0, p = 0.029 * |
| Heart | W = 3, p = 0.126 |
| Spleen | W = 7, p = 0.527 |
| Kidney | W = 6, p = 0.405 |
| Liver | W = 9, p = 0.527 |
| Pancreas | W = 5, p = 0.288 |
| Stomach | W = 10, p = 0.405 |
| Intestines | W = 4, p = 0.215 |

| Na  Tissue | Test-statistics |
| --- | --- |
| Bone | W = 7, p = 0.527 |
| Skin & hair | W = 7, p = 0.527 |
| Muscle | W = 7, p = 0.527 |
| Brain | W = 5, p = 0.288 |
| Eyes | W = 0, p = 0.029 * |
| Lungs | W = 3, p = 0.126 |
| Heart | W = 4, p = 0.215 |
| Spleen | W = 3, p = 0.126 |
| Kidney | W = 6, p = 0.405 |
| Liver | W = 4, p = 0.215 |
| Pancreas | W = 4, p = 0.215 |
| Stomach | W = 8, p = 0.584 |
| Intestines | W = 3, p = 0.126 |

| Ni  Tissue | Test-statistics |
| --- | --- |
| Bone | W = 8, p = 0.584 |
| Skin & hair | W = 8, p = 0.584 |
| Muscle | W = 9, p = 0.422 |
| Brain | W = 3, p = 0.126 |
| Eyes | W = 0, p = 0.029 * |
| Lungs | W = 7.5, p = 0.584 |
| Heart | W = 6, p = 0.302 |
| Spleen | W = 16, p = 0.029 * |
| Kidney | W = 7, p = 0.527 |
| Liver | W = 8, p = 0.584 |
| Pancreas | W = 8, p = 0.584 |
| Stomach | W = 15, p = 0.039 * |
| Intestines | W = 7, p = 0.527 |

| P  Tissue | Test-statistics |
| --- | --- |
| Bone | W = 7, p = 0.527 |
| Skin & hair | W = 6, p = 0.405 |
| Muscle | W = 7, p = 0.527 |
| Brain | W = 6, p = 0.405 |
| Eyes | W = 7, p = 0.527 |
| Lungs | W = 6, p = 0.405 |
| Heart | W = 6, p = 0.405 |
| Spleen | W = 7, p = 0.527 |
| Kidney | W = 9, p = 0.527 |
| Liver | W = 16, p = 0.029 * |
| Pancreas | W = 6, p = 0.405 |
| Stomach | W = 12, p = 0.215 |
| Intestines | W = 7, p = 0.527 |

| Pb  Tissue | Test-statistics |
| --- | --- |
| Bone | W = 7, p = 0.527 |
| Skin & hair | W = 13, p = 0.126 |
| Muscle | W = 10, p = 0.307 |
| Brain | W = 9, p = 0.422 |
| Eyes | W = 9, p = 0.415 |
| Lungs | W = 6, p = 0.405 |
| Heart | W = 11, p = 0.217 |
| Spleen | W = 12, p = 0.215 |
| Kidney | W = 9, p = 0.527 |
| Liver | W = 11, p = 0.288 |
| Pancreas | W = 9, p = 0.527 |
| Stomach | W = 14, p = 0.070 |
| Intestines | W = 6, p = 0.405 |

| S  Tissue | Test-statistics |
| --- | --- |
| Bone | W = 8, p = 0.584 |
| Skin & hair | W = 10, p = 0.405 |
| Muscle | W = 10, p = 0.405 |
| Brain | W = 6, p = 0.405 |
| Eyes | W = 7, p = 0.518 |
| Lungs | W = 2, p = 0.070 |
| Heart | W = 4, p = 0.215 |
| Spleen | W = 8, p = 0.584 |
| Kidney | W = 10, p = 0.405 |
| Liver | W = 12, p = 0.215 |
| Pancreas | W = 6, p = 0.405 |
| Stomach | W = 4, p = 0.215 |
| Intestines | W = 2, p = 0.070 |

| Se  Tissue | Test-statistics |
| --- | --- |
| Bone | W = 4, p = 0.215 |
| Skin & hair | W = 10, p = 0.405 |
| Muscle | W = 7, p = 0.527 |
| Brain | W = 12, p = 0.215 |
| Eyes | W = 6, p = 0.405 |
| Lungs | W = 6, p = 0.405 |
| Heart | W = 8, p = 0.584 |
| Spleen | W = 6, p = 0.405 |
| Kidney | W = 5, p = 0.288 |
| Liver | W = 8, p = 0.584 |
| Pancreas | W = 9, p = 0.527 |
| Stomach | W = 8, p = 0.584 |
| Intestines | W = 5, p = 0.288 |

| Si  Tissue | Test-statistics |
| --- | --- |
| Bone | W = 13, p = 0.126 |
| Skin & hair | W = 9, p = 0.527 |
| Muscle | W = 14, p = 0.044 * |
| Brain | W = 7, p = 0.527 |
| Eyes | W = 8.5, p = 0.584 |
| Lungs | W = 3, p = 0.126 |
| Heart | W = 5.5, p = 0.294 |
| Spleen | W = 8, p = 0.584 |
| Kidney | W = 6, p = 0.405 |
| Liver | W = 5, p = 0.233 |
| Pancreas | W = 6, p = 0.405 |
| Stomach | W = 12, p = 0.215 |
| Intestines | W = 3, p = 0.126 |

| Sr  Tissue | Test-statistics |
| --- | --- |
| Bone | W = 1, p = 0.039 * |
| Skin & hair | W = 5, p = 0.288 |
| Muscle | W = 8.5, p = 0.584 |
| Brain | W = 9, p = 0.527 |
| Eyes | W = 5, p = 0.288 |
| Lungs | W = 7, p = 0.527 |
| Heart | W = 4, p = 0.135 |
| Spleen | W = 11, p = 0.288 |
| Kidney | W = 1, p = 0.039 * |
| Liver | W = 6.5, p = 0.407 |
| Pancreas | W = 7, p = 0.527 |
| Stomach | W = 12, p = 0.215 |
| Intestines | W = 2, p = 0.070 |

| Zn  Tissue | Test-statistics |
| --- | --- |
| Bone | W = 5, p = 0.288 |
| Skin & hair | W = 11, p = 0.288 |
| Muscle | W = 16, p = 0.029 * |
| Brain | W = 9, p = 0.527 |
| Eyes | W = 6, p = 0.405 |
| Lungs | W = 6, p = 0.405 |
| Heart | W = 6, p = 0.405 |
| Spleen | W = 8, p = 0.584 |
| Kidney | W = 7, p = 0.527 |
| Liver | W = 9, p = 0.527 |
| Pancreas | W = 8, p = 0.584 |
| Stomach | W = 6, p = 0.405 |
| Intestines | W = 2, p = 0.070 |

**Test statistics of Mann-Whitney U tests for element-tissue combinations for sex classes in Fallow deer calves (adjusted p-values are reported after correction using the** **Benjamini and Hochberg procedure). * indicates significance.**

| Al  Tissue | Test-statistics |
| --- | --- |
| Bone | W = 11, p = 0.218 |
| Skin & hair | W = 11, p = 0.205 |
| Muscle | W = 10, p = 0.213 |
| Brain | W = 8, p = 0.213 |
| Eyes | W = 10, p = 0.213 |
| Lungs | W = 10, p = 0.213 |
| Heart | W = 6, p = 0.311 |
| Spleen | W = 10, p = 0.213 |
| Kidney | W = 14, p = 0.036 * |
| Liver | W = 11, p = 0.205 |
| Pancreas | W = 4, p = 0.105 |
| Stomach | W = 4, p = 0.156 |
| Intestines | W = 6, p = 0.316 |

| As  Tissue | Test-statistics |
| --- | --- |
| Bone | W = 12, p = 0.148 |
| Skin & hair | W = 9, p = 0.424 |
| Muscle | W = 1, p = 0.034 * |
| Brain | W = 12, p = 0.148 |
| Eyes | W = 8, p = 0.475 |
| Lungs | W = 4, p = 0.148 |
| Heart | W = 4, p = 0.148 |
| Spleen | W = 9, p = 0.424 |
| Kidney | W = 7, p = 0.424 |
| Liver | W = 3.5, p = 0.107 |
| Pancreas | W = 7, p = 0.424 |
| Stomach | W = 5, p = 0.202 |
| Intestines | W = 8, p = 0.475 |

| B  Tissue | Test-statistics |
| --- | --- |
| Bone | W = 11, p = 0.202 |
| Skin & hair | W = 6, p = 0.309 |
| Muscle | W = 5, p = 0.202 |
| Brain | W = 6, p = 0.309 |
| Eyes | W = 6, p = 0.309 |
| Lungs | W = 9, p = 0.424 |
| Heart | W = 9, p = 0.424 |
| Spleen | W = 4, p = 0.148 |
| Kidney | W = 9, p = 0.424 |
| Liver | W = 13, p = 0.104 |
| Pancreas | W = 5, p = 0.202 |
| Stomach | W = 3, p = 0.104 |
| Intestines | W = 8, p = 0.475 |

| Ca  Tissue | Test-statistics |
| --- | --- |
| Bone | W = 9, p = 0.424 |
| Skin & hair | W = 13, p = 0.104 |
| Muscle | W = 6, p = 0.309 |
| Brain | W = 9, p = 0.424 |
| Eyes | W = 7, p = 0.424 |
| Lungs | W = 7, p = 0.424 |
| Heart | W = 10, p = 0.309 |
| Spleen | W = 10, p = 0.309 |
| Kidney | W = 7, p = 0.424 |
| Liver | W = 8, p = 0.475 |
| Pancreas | W = 10, p = 0.309 |
| Stomach | W = 5, p = 0.202 |
| Intestines | W = 7, p = 0.424 |

| Cd  Tissue | Test-statistics |
| --- | --- |
| Bone | W = 8, p = 0.475 |
| Skin & hair | W = 12, p = 0.148 |
| Muscle | W = 3, p = 0.104 |
| Brain | W = 6, p = 0.309 |
| Eyes | W = 6, p = 0.309 |
| Lungs | W = 10, p = 0.309 |
| Heart | W = 6, p = 0.309 |
| Spleen | W = 6, p = 0.309 |
| Kidney | W = 8, p = 0.475 |
| Liver | W = 5, p = 0.202 |
| Pancreas | W = 13, p = 0.104 |
| Stomach | W = 8, p = 0.475 |
| Intestines | W = 11, p = 0.202 |

| Co  Tissue | Test-statistics |
| --- | --- |
| Bone | W = 10, p = 0.152 |
| Skin & hair | W = 11.5, p = 0.149 |
| Muscle | W = 6, p = 0.220 |
| Brain | W = 11.5, p = 0.151 |
| Eyes | W = 7, p = 0.318 |
| Lungs | W = 14, p = 0.041 * |
| Heart | W = 12, p = 0.109 |
| Spleen | W = 3, p = 0.104 |
| Kidney | W = 14, p = 0.062 |
| Liver | W = 10, p = 0.309 |
| Pancreas | W = 8.5, p = 0.429 |
| Stomach | W = 1, p = 0.034 * |
| Intestines | W = 10, p = 0.309 |

| Cr  Tissue | Test-statistics |
| --- | --- |
| Bone | W = 11, p = 0.154 |
| Skin & hair | W = 12, p = 0.148 |
| Muscle | W = 5.5, p = 0.216 |
| Brain | W = 8.5, p = 0.429 |
| Eyes | W = 9, p = 0.424 |
| Lungs | W = 14, p = 0.062 |
| Heart | W = 15, p = 0.024 * |
| Spleen | W = 5, p = 0.202 |
| Kidney | W = 14, p = 0.062 |
| Liver | W = 15, p = 0.034 * |
| Pancreas | W = 6.5, p = 0.314 |
| Stomach | W = 9, p = 0.424 |
| Intestines | W = 8, p = 0.475 |

| Cu  Tissue | Test-statistics |
| --- | --- |
| Bone | W = 7, p = 0.327 |
| Skin & hair | W = 9, p = 0.424 |
| Muscle | W = 6, p = 0.309 |
| Brain | W = 13, p = 0.104 |
| Eyes | W = 14, p = 0.062 |
| Lungs | W = 13, p = 0.104 |
| Heart | W = 9, p = 0.424 |
| Spleen | W = 12, p = 0.148 |
| Kidney | W = 13, p = 0.104 |
| Liver | W = 5, p = 0.202 |
| Pancreas | W = 13, p = 0.148 |
| Stomach | W = 13, p = 0.104 |
| Intestines | W = 11, p = 0.202 |

| Fe  Tissue | Test-statistics |
| --- | --- |
| Bone | W = NaN, p = NaN |
| Skin & hair | W = 12, p = 0.065 |
| Muscle | W = 12.5, p = 0.107 |
| Brain | W = 11, p = 0.202 |
| Eyes | W = 4, p = 0.065 |
| Lungs | W = 3, p = 0.104 |
| Heart | W = 8, p = 0.475 |
| Spleen | W = 4, p = 0.148 |
| Kidney | W = 8, p = 0.475 |
| Liver | W = 3, p = 0.104 |
| Pancreas | W = 4, p = 0.148 |
| Stomach | W = 0, p = 0.021 * |
| Intestines | W = 9.5, p = 0.314 |

| K  Tissue | Test-statistics |
| --- | --- |
| Bone | W = 8, p = 0.475 |
| Skin & hair | W = 10, p = 0.309 |
| Muscle | W = 9, p = 0.424 |
| Brain | W = 11, p = 0.202 |
| Eyes | W = 6, p = 0.309 |
| Lungs | W = 15, p = 0.034 * |
| Heart | W = 9, p = 0.424 |
| Spleen | W = 14, p = 0.062 |
| Kidney | W = 11, p = 0.202 |
| Liver | W = 8, p = 0.475 |
| Pancreas | W = 13, p = 0.104 |
| Stomach | W = 10, p = 0.309 |
| Intestines | W = 13, p = 0.104 |

| Mg  Tissue | Test-statistics |
| --- | --- |
| Bone | W = 8, p = 0.475 |
| Skin & hair | W = 5, p = 0.202 |
| Muscle | W = 9, p = 0.424 |
| Brain | W = 9, p = 0.424 |
| Eyes | W = 7, p = 0.424 |
| Lungs | W = 16, p = 0.021 * |
| Heart | W = 7, p = 0.424 |
| Spleen | W = 14, p = 0.062 |
| Kidney | W = 10, p = 0.309 |
| Liver | W = 9, p = 0.424 |
| Pancreas | W = 12, p = 0.148 |
| Stomach | W = 6, p = 0.309 |
| Intestines | W = 9, p = 0.424 |

| Mn  Tissue | Test-statistics |
| --- | --- |
| Bone | W = 8, p = 0.475 |
| Skin & hair | W = 12, p = 0.148 |
| Muscle | W = 14, p = 0.062 |
| Brain | W = 4, p = 0.148 |
| Eyes | W = 11, p = 0.202 |
| Lungs | W = 7, p = 0.424 |
| Heart | W = 9, p = 0.424 |
| Spleen | W = 8, p = 0.475 |
| Kidney | W = 10, p = 0.309 |
| Liver | W = 6, p = 0.309 |
| Pancreas | W = 5, p = 0.202 |
| Stomach | W = 7, p = 0.424 |
| Intestines | W = 8, p = 0.475 |

| Mo  Tissue | Test-statistics |
| --- | --- |
| Bone | W = 10, p = 0.225 |
| Skin & hair | W = 11, p = 0.202 |
| Muscle | W = 12, p = 0.148 |
| Brain | W = 9, p = 0.424 |
| Eyes | W = 7, p = 0.424 |
| Lungs | W = 12, p = 0.148 |
| Heart | W = 12, p = 0.148 |
| Spleen | W = 10, p = 0.309 |
| Kidney | W = 12, p = 0.148 |
| Liver | W = 9, p = 0.424 |
| Pancreas | W = 14, p = 0.062 |
| Stomach | W = 3, p = 0.104 |
| Intestines | W = 9, p = 0.424 |

| Na  Tissue | Test-statistics |
| --- | --- |
| Bone | W = 15, p = 0.034 * |
| Skin & hair | W = 9, p = 0.424 |
| Muscle | W = 10, p = 0.309 |
| Brain | W = 5, p = 0.202 |
| Eyes | W = 7, p = 0.424 |
| Lungs | W = 11, p = 0.202 |
| Heart | W = 7, p = 0.424 |
| Spleen | W = 14, p = 0.062 |
| Kidney | W = 7, p = 0.424 |
| Liver | W = 4, p = 0.148 |
| Pancreas | W = 13, p = 0.104 |
| Stomach | W = 13, p = 0.104 |
| Intestines | W = 14, p = 0.062 |

| Ni  Tissue | Test-statistics |
| --- | --- |
| Bone | W = 9, p = 0.322 |
| Skin & hair | W = 11, p = 0.158 |
| Muscle | W = 8, p = 0.475 |
| Brain | W = 10.5, p = 0.216 |
| Eyes | W = 13, p = 0.104 |
| Lungs | W = 15, p = 0.034 * |
| Heart | W = 13, p = 0.069 |
| Spleen | W = 4, p = 0.148 |
| Kidney | W = 14, p = 0.062 |
| Liver | W = 13, p = 0.071 |
| Pancreas | W = 3, p = 0.104 |
| Stomach | W = 4, p = 0.148 |
| Intestines | W = 12, p = 0.148 |

| P  Tissue | Test-statistics |
| --- | --- |
| Bone | W = 9, p = 0.424 |
| Skin & hair | W = 13, p = 0.104 |
| Muscle | W = 9, p = 0.424 |
| Brain | W = 9, p = 0.424 |
| Eyes | W = 10, p = 0.309 |
| Lungs | W = 16, p = 0.021 * |
| Heart | W = 7, p = 0.424 |
| Spleen | W = 15, p = 0.034 * |
| Kidney | W = 11, p = 0.202 |
| Liver | W = 10, p = 0.309 |
| Pancreas | W = 10, p = 0.309 |
| Stomach | W = 6, p = 0.309 |
| Intestines | W = 11, p = 0.202 |

| Pb  Tissue | Test-statistics |
| --- | --- |
| Bone | W = 2, p = 0.062 |
| Skin & hair | W = 9, p = 0.424 |
| Muscle | W = 7.5, p = 0.429 |
| Brain | W = 7, p = 0.322 |
| Eyes | W = 10, p = 0.222 |
| Lungs | W = 7, p = 0.424 |
| Heart | W = 13, p = 0.069 |
| Spleen | W = 7, p = 0.424 |
| Kidney | W = 10, p = 0.309 |
| Liver | W = 7, p = 0.424 |
| Pancreas | W = 7, p = 0.424 |
| Stomach | W = 2, p = 0.062 |
| Intestines | W = 6, p = 0.309 |

| S  Tissue | Test-statistics |
| --- | --- |
| Bone | W = 8, p = 0.475 |
| Skin & hair | W = 6, p = 0.309 |
| Muscle | W = 6, p = 0.309 |
| Brain | W = 10, p = 0.309 |
| Eyes | W = 4, p = 0.148 |
| Lungs | W = 16, p = 0.021 * |
| Heart | W = 9, p = 0.424 |
| Spleen | W = 13, p = 0.104 |
| Kidney | W = 5, p = 0.202 |
| Liver | W = 4, p = 0.148 |
| Pancreas | W = 13, p = 0.104 |
| Stomach | W = 12, p = 0.148 |
| Intestines | W = 14, p = 0.062 |

| Se  Tissue | Test-statistics |
| --- | --- |
| Bone | W = 9, p = 0.424 |
| Skin & hair | W = 4, p = 0.148 |
| Muscle | W = 7, p = 0.424 |
| Brain | W = 0, p = 0.021 * |
| Eyes | W = 3, p = 0.104 |
| Lungs | W = 6, p = 0.309 |
| Heart | W = 7, p = 0.424 |
| Spleen | W = 10, p = 0.309 |
| Kidney | W = 8, p = 0.475 |
| Liver | W = 6, p = 0.309 |
| Pancreas | W = 9, p = 0.424 |
| Stomach | W = 3, p = 0.104 |
| Intestines | W = 10, p = 0.309 |

| Si  Tissue | Test-statistics |
| --- | --- |
| Bone | W = 11, p = 0.202 |
| Skin & hair | W = 10, p = 0.309 |
| Muscle | W = 4, p = 0.148 |
| Brain | W = 10, p = 0.309 |
| Eyes | W = 9, p = 0.424 |
| Lungs | W = 10, p = 0.309 |
| Heart | W = 13, p = 0.104 |
| Spleen | W = 7, p = 0.424 |
| Kidney | W = 14, p = 0.062 |
| Liver | W = 12, p = 0.111 |
| Pancreas | W = 10, p = 0.309 |
| Stomach | W = 5, p = 0.202 |
| Intestines | W = 9, p = 0.424 |

| Sr  Tissue | Test-statistics |
| --- | --- |
| Bone | W = 6, p = 0.309 |
| Skin & hair | W = 13, p = 0.104 |
| Muscle | W = 7, p = 0.327 |
| Brain | W = 8, p = 0.475 |
| Eyes | W = 10, p = 0.309 |
| Lungs | W = 8, p = 0.475 |
| Heart | W = 14, p = 0.041 * |
| Spleen | W = 7, p = 0.424 |
| Kidney | W = 14, p = 0.062 |
| Liver | W = 10, p = 0.309 |
| Pancreas | W = 7, p = 0.424 |
| Stomach | W = 5, p = 0.202 |
| Intestines | W = 6, p = 0.309 |

| Zn  Tissue | Test-statistics |
| --- | --- |
| Bone | W = 10, p = 0.309 |
| Skin & hair | W = 6, p = 0.309 |
| Muscle | W = 2, p = 0.062 |
| Brain | W = 9, p = 0.424 |
| Eyes | W = 8, p = 0.475 |
| Lungs | W = 15, p = 0.034 * |
| Heart | W = 8, p = 0.475 |
| Spleen | W = 13, p = 0.104 |
| Kidney | W = 10, p = 0.309 |
| Liver | W = 5, p = 0.202 |
| Pancreas | W = 13, p = 0.104 |
| Stomach | W = 16, p = 0.021 * |
| Intestines | W = 13, p = 0.104 |

**Test statistics of Mann-Whitney U tests for element-tissue combinations for sex classes in Fallow deer calves (adjusted p-values are reported after correction using the** **Benjamini and Hochberg procedure). * indicates significance.**

| Al  Tissue | Test-statistics |
| --- | --- |
| Bone | W = 13, p = 0.101 |
| Skin & hair | W = 11, p = 0.227 |
| Muscle | W = 7, p = 0.440 |
| Brain | W = NaN, p = NaN |
| Eyes | W = NaN, p = NaN |
| Lungs | W = 10, p = 0.332 |
| Heart | W = 6, p = 0.332 |
| Spleen | W = 6, p = 0.229 |
| Kidney | W = 6, p = 0.334 |
| Liver | W = 9, p = 0.440 |
| Pancreas | W = 8.5, p = 0.499 |
| Stomach | W = 12, p = 0.171 |
| Intestines | W = 10, p = 0.337 |

| As  Tissue | Test-statistics |
| --- | --- |
| Bone | W = 9, p = 0.431 |
| Skin & hair | W = 8, p = 0.499 |
| Muscle | W = 7, p = 0.431 |
| Brain | W = 6, p = 0.238 |
| Eyes | W = 9, p = 0.431 |
| Lungs | W = 3, p = 0.095 |
| Heart | W = 4, p = 0.169 |
| Spleen | W = 11, p = 0.226 |
| Kidney | W = 12, p = 0.169 |
| Liver | W = 11, p = 0.226 |
| Pancreas | W = 3, p = 0.095 |
| Stomach | W = 10, p = 0.329 |
| Intestines | W = 6, p = 0.329 |

| B  Tissue | Test-statistics |
| --- | --- |
| Bone | W = 8, p = 0.449 |
| Skin & hair | W = 8, p = 0.449 |
| Muscle | W = 4, p = 0.169 |
| Brain | W = 5, p = 0.226 |
| Eyes | W = 7, p = 0.431 |
| Lungs | W = 2, p = 0.050 * |
| Heart | W = 7, p = 0.431 |
| Spleen | W = 12, p = 0.169 |
| Kidney | W = 3, p = 0.095 |
| Liver | W = 16, p = 0.021 * |
| Pancreas | W = 10, p = 0.329 |
| Stomach | W = 12, p = 0.169 |
| Intestines | W = 6, p = 0.329 |
| Ca  Tissue | Test-statistics |
| Bone | W = 11, p = 0.226 |
| Skin & hair | W = 7, p = 0.431 |
| Muscle | W = 11, p = 0.226 |
| Brain | W = 10, p = 0.329 |
| Eyes | W = 4, p = 0.169 |
| Lungs | W = 7, p = 0.431 |
| Heart | W = 7, p = 0.431 |
| Spleen | W = 12, p = 0.169 |
| Kidney | W = 5, p = 0.226 |
| Liver | W = 6, p = 0.329 |
| Pancreas | W = 6, p = 0.329 |
| Stomach | W = 10, p = 0.329 |
| Intestines | W = 10, p = 0.329 |

| Cd  Tissue | Test-statistics |
| --- | --- |
| Bone | W = 10, p = 0.329 |
| Skin & hair | W = 10, p = 0.329 |
| Muscle | W = 7, p = 0.431 |
| Brain | W = 12, p = 0.169 |
| Eyes | W = 10, p = 0.329 |
| Lungs | W = 5, p = 0.226 |
| Heart | W = 10, p = 0.329 |
| Spleen | W = 9, p = 0.431 |
| Kidney | W = 10, p = 0.329 |
| Liver | W = 11, p = 0.226 |
| Pancreas | W = 6, p = 0.329 |
| Stomach | W = 12, p = 0.169 |
| Intestines | W = 6, p = 0.329 |

| Co  Tissue | Test-statistics |
| --- | --- |
| Bone | W = 10, p = 0.176 |
| Skin & hair | W = 9, p = 0.341 |
| Muscle | W = 8.5, p = 0.436 |
| Brain | W = 3, p = 0.095 |
| Eyes | W = 1, p = 0.031 * |
| Lungs | W = 7.5, p = 0.436 |
| Heart | W = 10, p = 0.238 |
| Spleen | W = 12.5, p = 0.098 |
| Kidney | W = 10, p = 0.329 |
| Liver | W = 16, p = 0.021 * |
| Pancreas | W = 8, p = 0.499 |
| Stomach | W = 13, p = 0.095 |
| Intestines | W = 11, p = 0.226 |

| Cr  Tissue | Test-statistics |
| --- | --- |
| Bone | W = 12.5, p = 0.098 |
| Skin & hair | W = 9, p = 0.431 |
| Muscle | W = 12, p = 0.111 |
| Brain | W = 9, p = 0.431 |
| Eyes | W = 4, p = 0.169 |
| Lungs | W = 11, p = 0.182 |
| Heart | W = 10, p = 0.329 |
| Spleen | W = 12, p = 0.169 |
| Kidney | W = 9, p = 0.431 |
| Liver | W = 15, p = 0.023 * |
| Pancreas | W = 12, p = 0.111 |
| Stomach | W = 10, p = 0.329 |
| Intestines | W = 5, p = 0.226 |

| Cu  Tissue | Test-statistics |
| --- | --- |
| Bone | W = 6, p = 0.176 |
| Skin & hair | W = 8, p = 0.499 |
| Muscle | W = 5, p = 0.226 |
| Brain | W = 14, p = 0.050 * |
| Eyes | W = 13, p = 0.095 |
| Lungs | W = 5, p = 0.226 |
| Heart | W = 2, p = 0.050 * |
| Spleen | W = 10, p = 0.329 |
| Kidney | W = 8, p = 0.499 |
| Liver | W = 3, p = 0.0.95 |
| Pancreas | W = 6, p = 0.329 |
| Stomach | W = 5, p = 0.226 |
| Intestines | W = 8, p = 0.499 |

| Fe  Tissue | Test-statistics |
| --- | --- |
| Bone | W = 10, p = 0.176 |
| Skin & hair | W = 8.5, p = 0.436 |
| Muscle | W = 6, p = 0.329 |
| Brain | W = 11, p = 0.226 |
| Eyes | W = 11, p = 0.182 |
| Lungs | W = 3, p = 0.095 |
| Heart | W = 5, p = 0.226 |
| Spleen | W = 7, p = 0.431 |
| Kidney | W = 9, p = 0.431 |
| Liver | W = 1, p = 0.031 * |
| Pancreas | W = 0, p = 0.021 * |
| Stomach | W = 11, p = 0.226 |
| Intestines | W = 3.5, p = 0.099 |

| K  Tissue | Test-statistics |
| --- | --- |
| Bone | W = 3, p = 0.095 |
| Skin & hair | W = 13, p = 0.095 |
| Muscle | W = 6, p = 0.329 |
| Brain | W = 11, p = 0.226 |
| Eyes | W = 6, p = 0.329 |
| Lungs | W = 6, p = 0.329 |
| Heart | W = 4, p = 0.169 |
| Spleen | W = 10, p = 0.329 |
| Kidney | W = 7, p = 0.431 |
| Liver | W = 14, p = 0.050 * |
| Pancreas | W = 6, p = 0.329 |
| Stomach | W = 6, p = 0.329 |
| Intestines | W = 0, p = 0.021 * |

| Mg  Tissue | Test-statistics |
| --- | --- |
| Bone | W = 15, p = 0.031 * |
| Skin & hair | W = 12, p = 0.169 |
| Muscle | W = 4, p = 0.169 |
| Brain | W = 4, p = 0.169 |
| Eyes | W = 8, p = 0.499 |
| Lungs | W = 10, p = 0.329 |
| Heart | W = 4, p = 0.169 |
| Spleen | W = 13, p = 0.095 |
| Kidney | W = 6, p = 0.329 |
| Liver | W = 12, p = 0.169 |
| Pancreas | W = 4, p = 0.169 |
| Stomach | W = 9, p = 0.431 |
| Intestines | W = 13, p = 0.095 |

| Mn  Tissue | Test-statistics |
| --- | --- |
| Bone | W = 7, p = 0.431 |
| Skin & hair | W = 6, p = 0.329 |
| Muscle | W = 9, p = 0.431 |
| Brain | W = 10, p = 0.329 |
| Eyes | W = 14, p = 0.050 * |
| Lungs | W = 11, p = 0.226 |
| Heart | W = 9, p = 0.431 |
| Spleen | W = 11, p = 0.226 |
| Kidney | W = 3, p = 0.095 |
| Liver | W = 2, p = 0.050 * |
| Pancreas | W = 4, p = 0.169 |
| Stomach | W = 10, p = 0.329 |
| Intestines | W = 12, p = 0.169 |

| Mo  Tissue | Test-statistics |
| --- | --- |
| Bone | W = 16, p = 0.021 * |
| Skin & hair | W = 6, p = 0.329 |
| Muscle | W = 7, p = 0.431 |
| Brain | W = 10, p = 0.329 |
| Eyes | W = 6, p = 0.329 |
| Lungs | W = 5, p = 0.226 |
| Heart | W = 10, p = 0.329 |
| Spleen | W = 13, p = 0.095 |
| Kidney | W = 7, p = 0.431 |
| Liver | W =12 , p = 0.169 |
| Pancreas | W = 6, p = 0.329 |
| Stomach | W = 10, p = 0.329 |
| Intestines | W = 7, p = 0.431 |

| Na  Tissue | Test-statistics |
| --- | --- |
| Bone | W = 13, p = 0.095 |
| Skin & hair | W = 8, p = 0.499 |
| Muscle | W = 12, p = 0.169 |
| Brain | W = 7, p = 0.431 |
| Eyes | W = 1, p = 0.031 * |
| Lungs | W = 6, p = 0.329 |
| Heart | W = 0, p = 0.021 * |
| Spleen | W = 11, p = 0.226 |
| Kidney | W = 9, p = 0.431 |
| Liver | W = 1, p = 0.031 * |
| Pancreas | W = 4, p = 0.169 |
| Stomach | W = 8, p = 0.499 |
| Intestines | W = 4, p = 0.169 |

| Ni  Tissue | Test-statistics |
| --- | --- |
| Bone | W = 10, p = 0.233 |
| Skin & hair | W = 9, p = 0.346 |
| Muscle | W = 7, p = 0.339 |
| Brain | W = 0, p = 0.021 * |
| Eyes | W = 6, p = 0.329 |
| Lungs | W = 5, p = 0.226 |
| Heart | W = 9, p = 0.346 |
| Spleen | W = 13, p = 0.054 |
| Kidney | W = 8, p = 0.499 |
| Liver | W = 11, p = 0.182 |
| Pancreas | W = 8, p = 0.499 |
| Stomach | W = 9, p = 0.431 |
| Intestines | W = 5, p = 0.226 |

| P  Tissue | Test-statistics |
| --- | --- |
| Bone | W = 12, p = 0.169 |
| Skin & hair | W = 8, p = 0.499 |
| Muscle | W = 6, p = 0.329 |
| Brain | W = 9, p = 0.431 |
| Eyes | W = 8, p = 0.499 |
| Lungs | W = 10, p = 0.329 |
| Heart | W = 4, p = 0.169 |
| Spleen | W = 12, p = 0.169 |
| Kidney | W = 6, p = 0.329 |
| Liver | W = 12, p = 0.169 |
| Pancreas | W = 4, p = 0.169 |
| Stomach | W = 8, p = 0.499 |
| Intestines | W = 7, p = 0.431 |

| Pb  Tissue | Test-statistics |
| --- | --- |
| Bone | W = 10, p = 0.329 |
| Skin & hair | W = 8, p = 0.499 |
| Muscle | W = 6, p = 0.231 |
| Brain | W = 12, p = 0.116 |
| Eyes | W = 11.5, p = 0.170 |
| Lungs | W = 8, p = 0.499 |
| Heart | W = 13.5, p = 0.051 |
| Spleen | W = 9, p = 0.431 |
| Kidney | W = 9, p = 0.431 |
| Liver | W = 9, p = 0.431 |
| Pancreas | W = 3, p = 0.095 |
| Stomach | W = 8, p = 0.499 |
| Intestines | W = 8, p = 0.499 |

| S  Tissue | Test-statistics |
| --- | --- |
| Bone | W = 13, p = 0.095 |
| Skin & hair | W = 7, p = 0.431 |
| Muscle | W = 13, p = 0.095 |
| Brain | W = 8, p = 0.499 |
| Eyes | W = 9, p = 0.431 |
| Lungs | W = 7, p = 0.431 |
| Heart | W = 4, p = 0.169 |
| Spleen | W = 11, p = 0.226 |
| Kidney | W = 6, p = 0.329 |
| Liver | W = 6, p = 0.329 |
| Pancreas | W = 8, p = 0.499 |
| Stomach | W = 4, p = 0.169 |
| Intestines | W = 1, p = 0.031 * |

| Se  Tissue | Test-statistics |
| --- | --- |
| Bone | W = 2, p = 0.050 * |
| Skin & hair | W = 9, p = 0.431 |
| Muscle | W = 9, p = 0.431 |
| Brain | W = 13, p = 0.095 |
| Eyes | W = 7, p = 0.431 |
| Lungs | W = 8, p = 0.499 |
| Heart | W = 11, p = 0.226 |
| Spleen | W = 14, p = 0.050 * |
| Kidney | W = 8, p = 0.499 |
| Liver | W = 7, p = 0.431 |
| Pancreas | W = 8, p = 0.499 |
| Stomach | W = 11, p = 0.226 |
| Intestines | W = 9, p = 0.431 |

| Si  Tissue | Test-statistics |
| --- | --- |
| Bone | W = 4, p = 0.111 |
| Skin & hair | W = 8, p = 0.499 |
| Muscle | W = 12, p = 0.116 |
| Brain | W = 7, p = 0.431 |
| Eyes | W = 7, p = 0.431 |
| Lungs | W = 8, p = 0.499 |
| Heart | W = 12, p = 0.169 |
| Spleen | W = 10, p = 0.329 |
| Kidney | W = 6, p = 0.329 |
| Liver | W = 7, p = 0.431 |
| Pancreas | W = 5, p = 0.226 |
| Stomach | W = 10, p = 0.329 |
| Intestines | W = 4, p = 0.169 |

| Sr  Tissue | Test-statistics |
| --- | --- |
| Bone | W = 3, p = 0.095 |
| Skin & hair | W = 7, p = 0.431 |
| Muscle | W = 4, p = 0.111 |
| Brain | W = 9, p = 0.431 |
| Eyes | W = 8, p = 0.499 |
| Lungs | W = 8, p = 0.499 |
| Heart | W = 16, p = 0.021 * |
| Spleen | W = 12, p = 0.169 |
| Kidney | W = 2, p = 0.050 * |
| Liver | W = 6.5, p = 0.335 |
| Pancreas | W = 4, p = 0.169 |
| Stomach | W = 10, p = 0.329 |
| Intestines | W = 4, p = 0.169 |

| Zn  Tissue | Test-statistics |
| --- | --- |
| Bone | W = 12, p = 0.169 |
| Skin & hair | W = 9, p = 0.431 |
| Muscle | W = 16, p = 0.021 * |
| Brain | W = 9, p = 0.431 |
| Eyes | W = 10, p = 0.329 |
| Lungs | W = 9, p = 0.431 |
| Heart | W = 5, p = 0.226 |
| Spleen | W = 12, p = 0.169 |
| Kidney | W = 9, p = 0.431 |
| Liver | W = 7, p = 0.431 |
| Pancreas | W = 6, p = 0.329 |
| Stomach | W = 8, p = 0.499 |
| Intestines | W = 7, p = 0.431 |
