# Supplementary material for: Ionomic Variation Among Tissues in Fallow Deer (Dama dama) by Sex and Age
Source: Biol Trace Elem Res. 2023 Jun 8;202(3):965–79. doi: 10.1007/s12011-023-03724-x (PMC10803548; doi:10.1007/s12011-023-03724-x)
Supplement: Supplementary file 3 — Supplementary Material 3 [file 12011_2023_3724_MOESM3_ESM.docx]

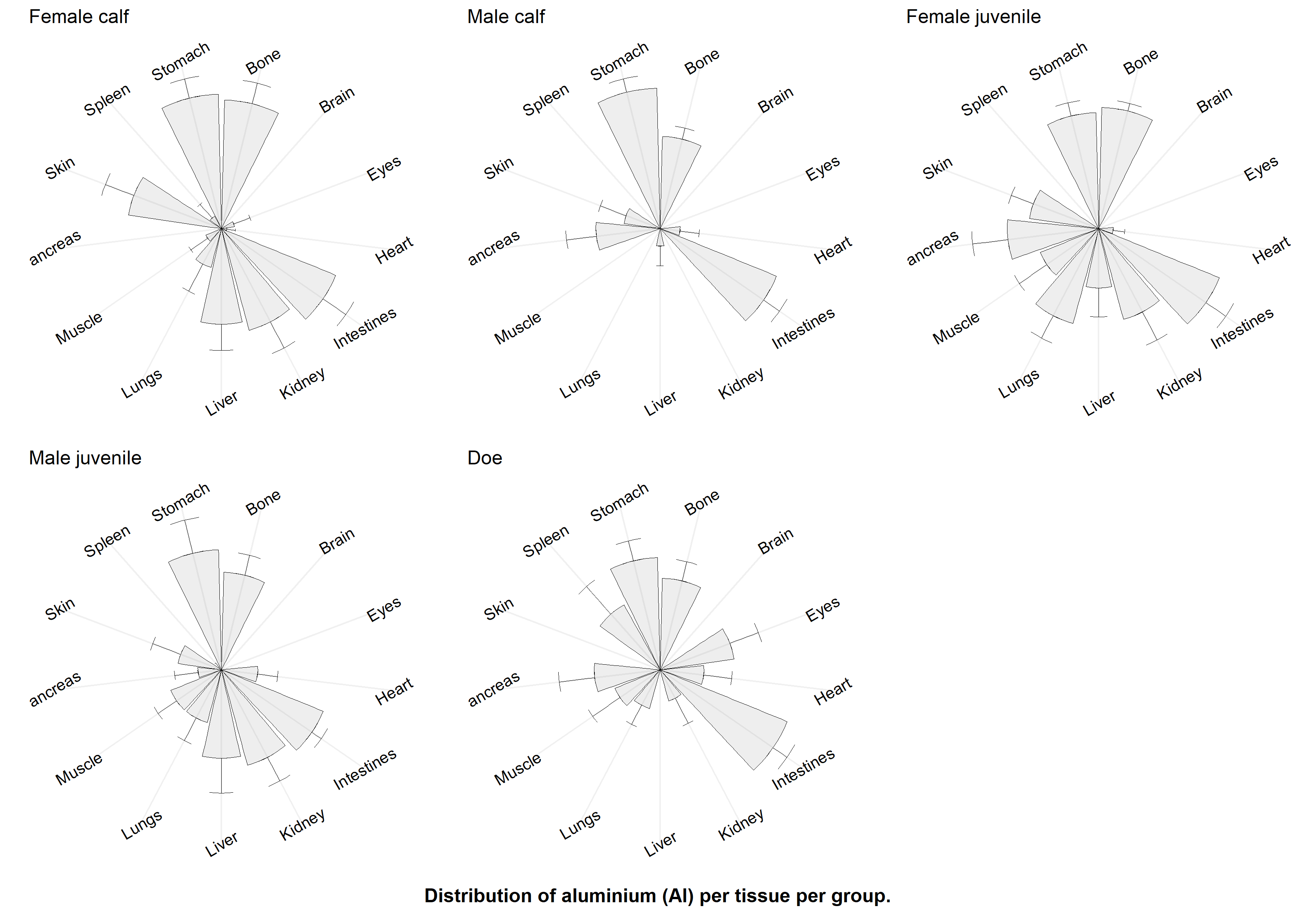


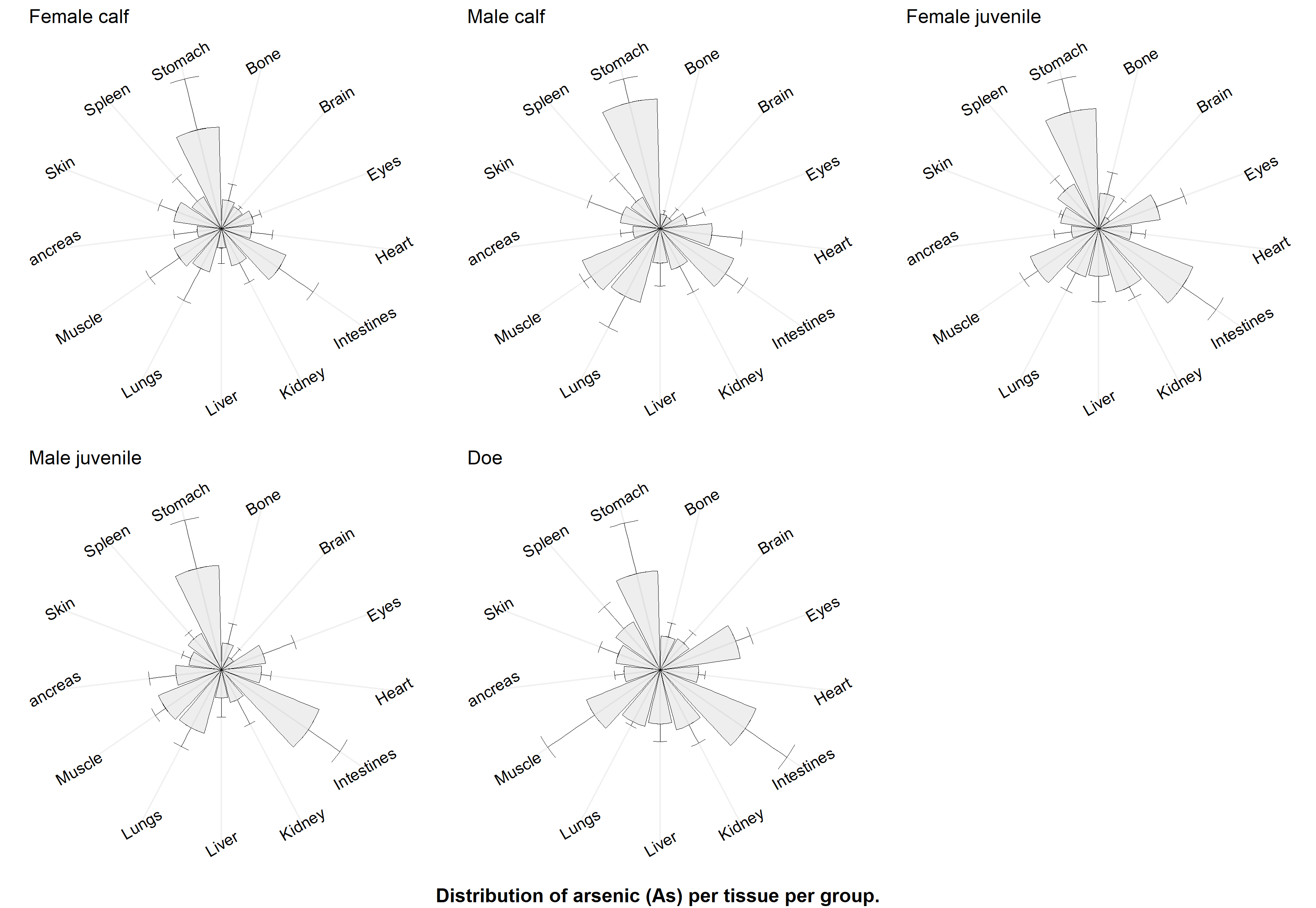


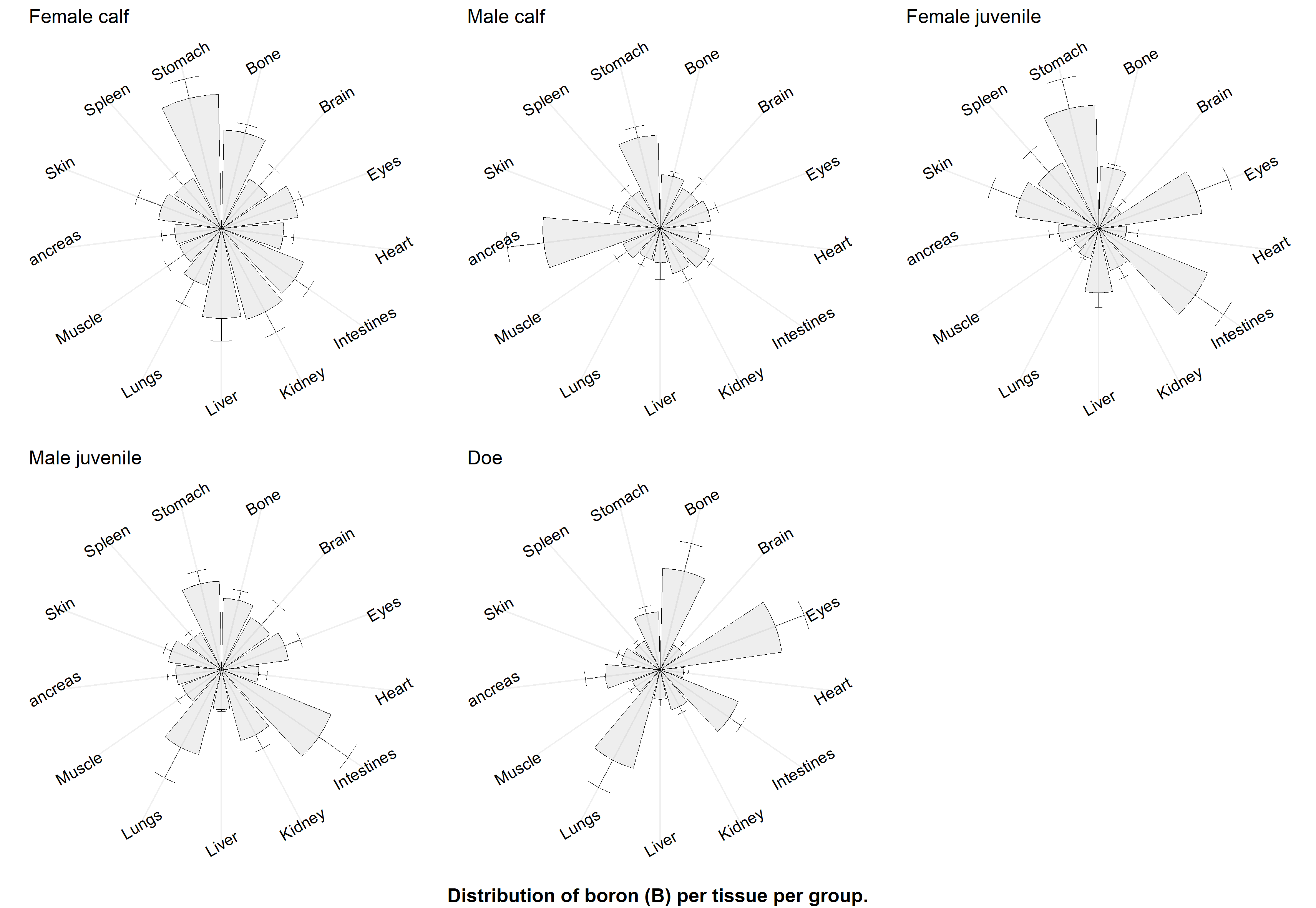


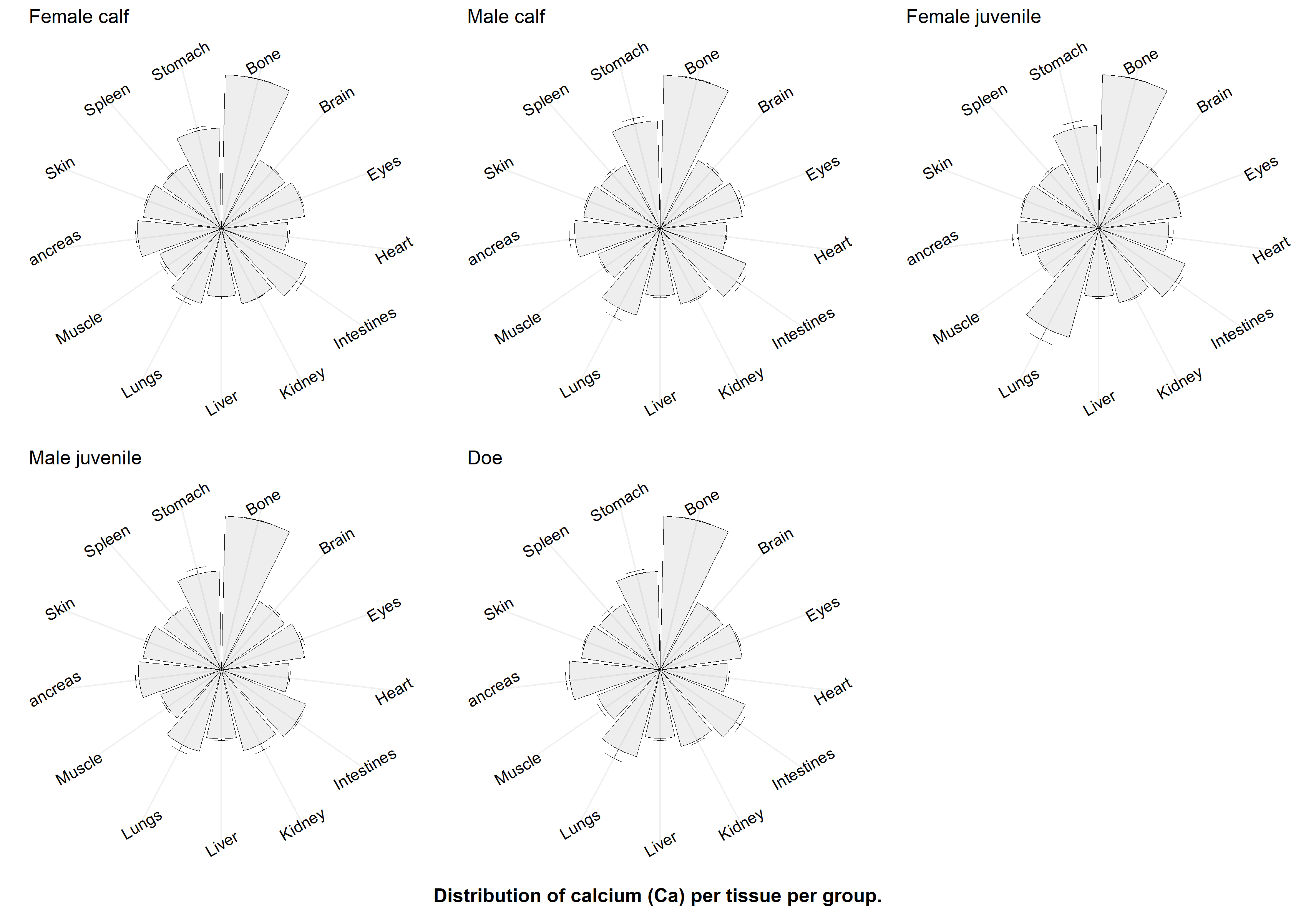


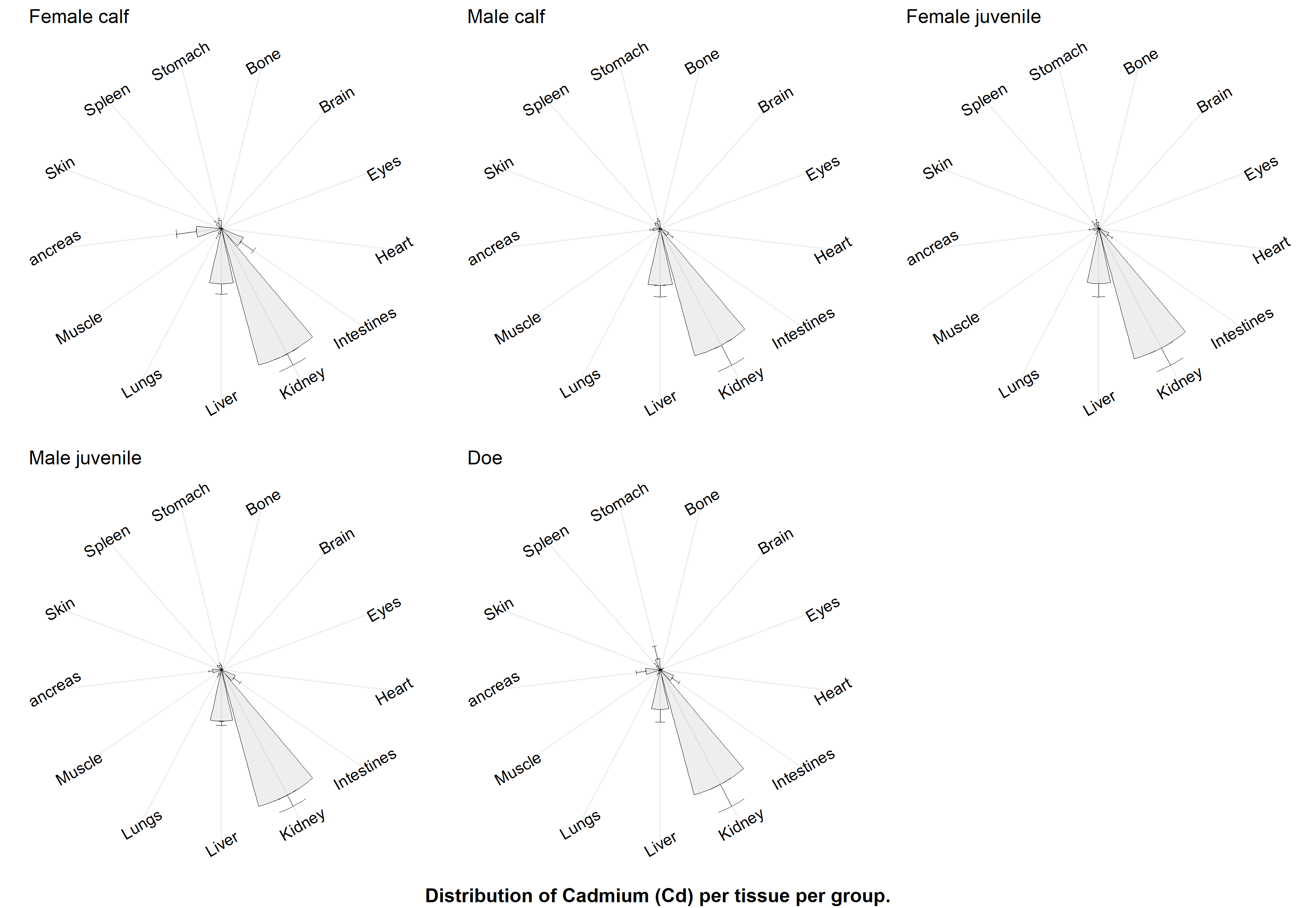


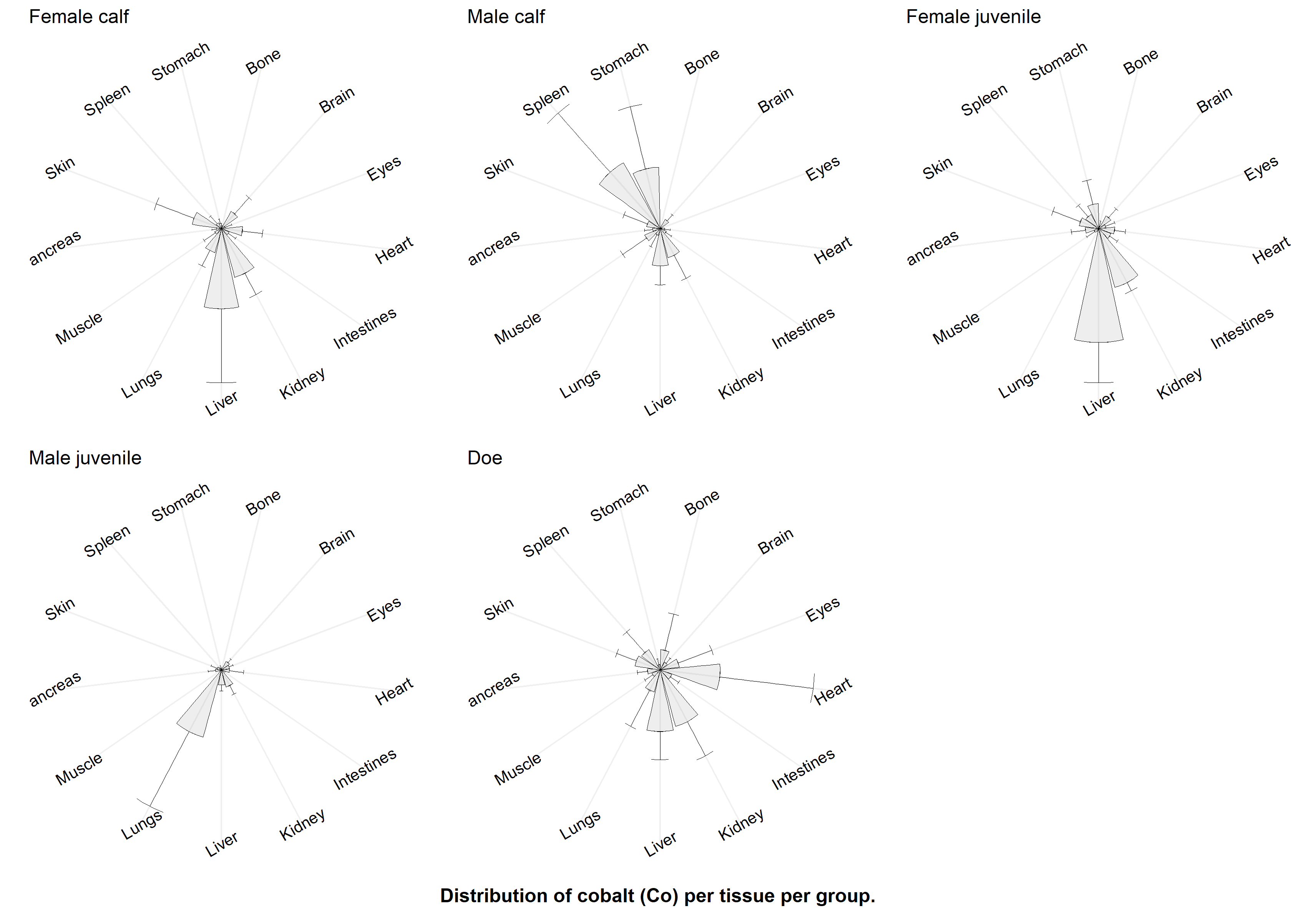


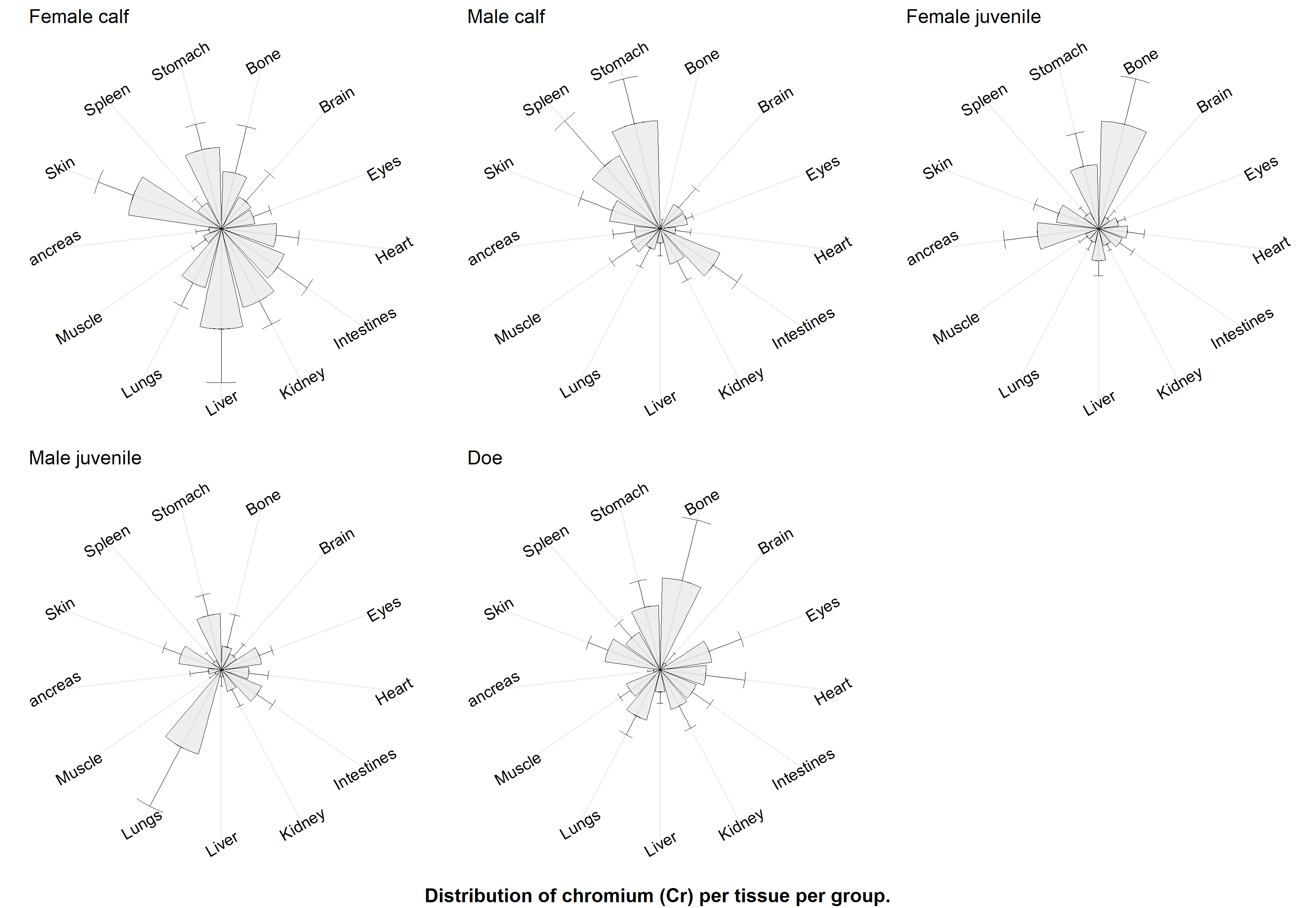


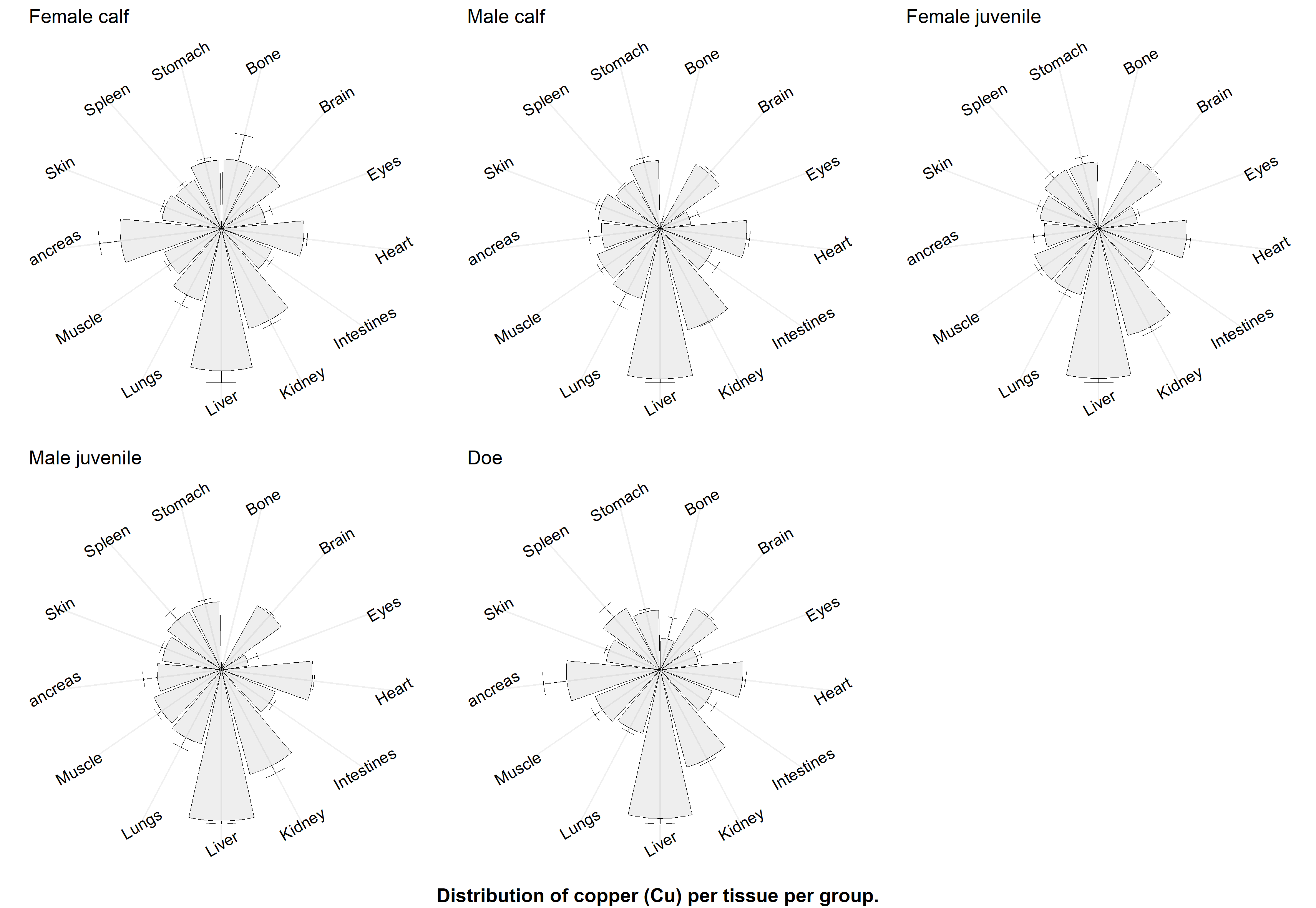


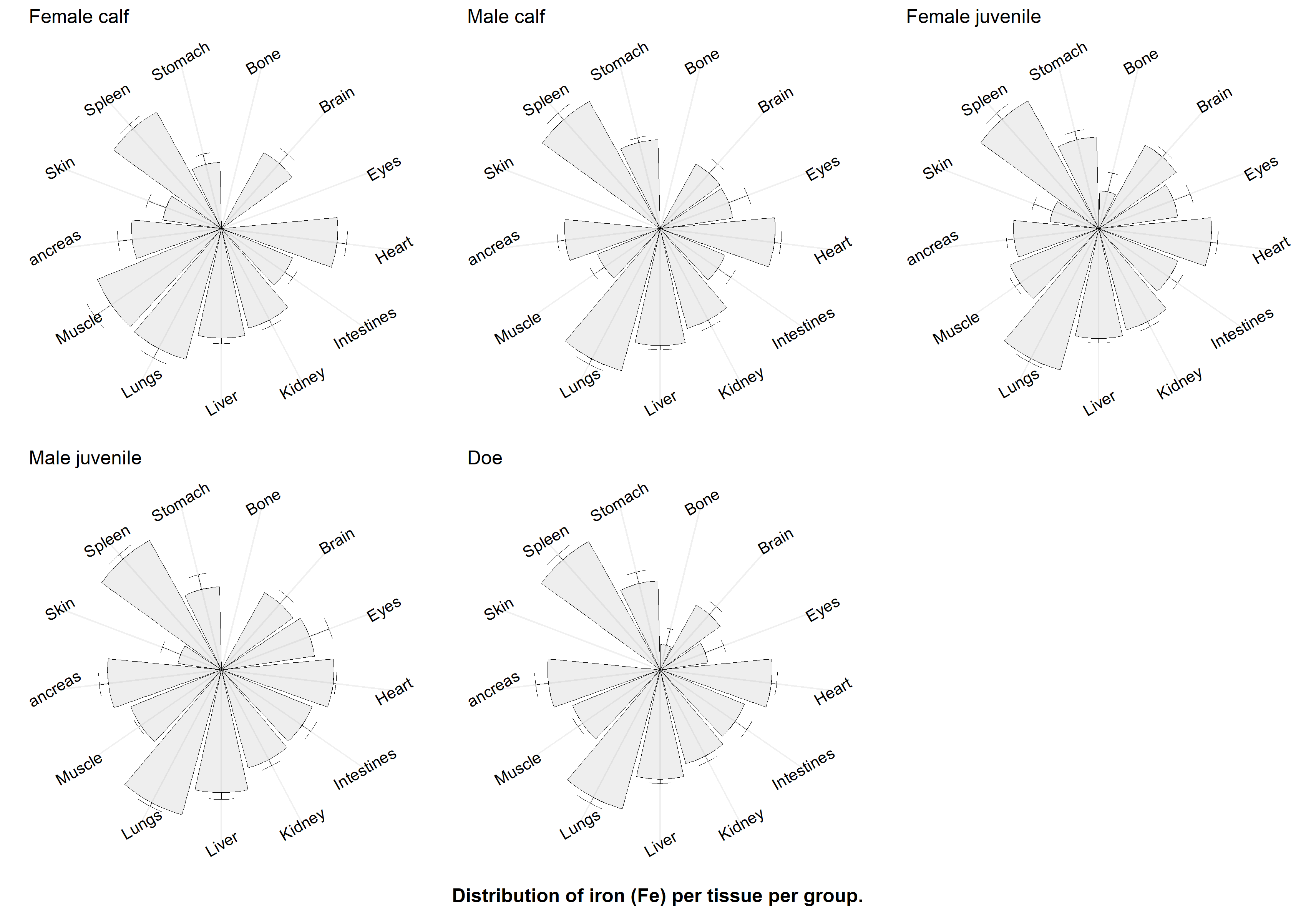


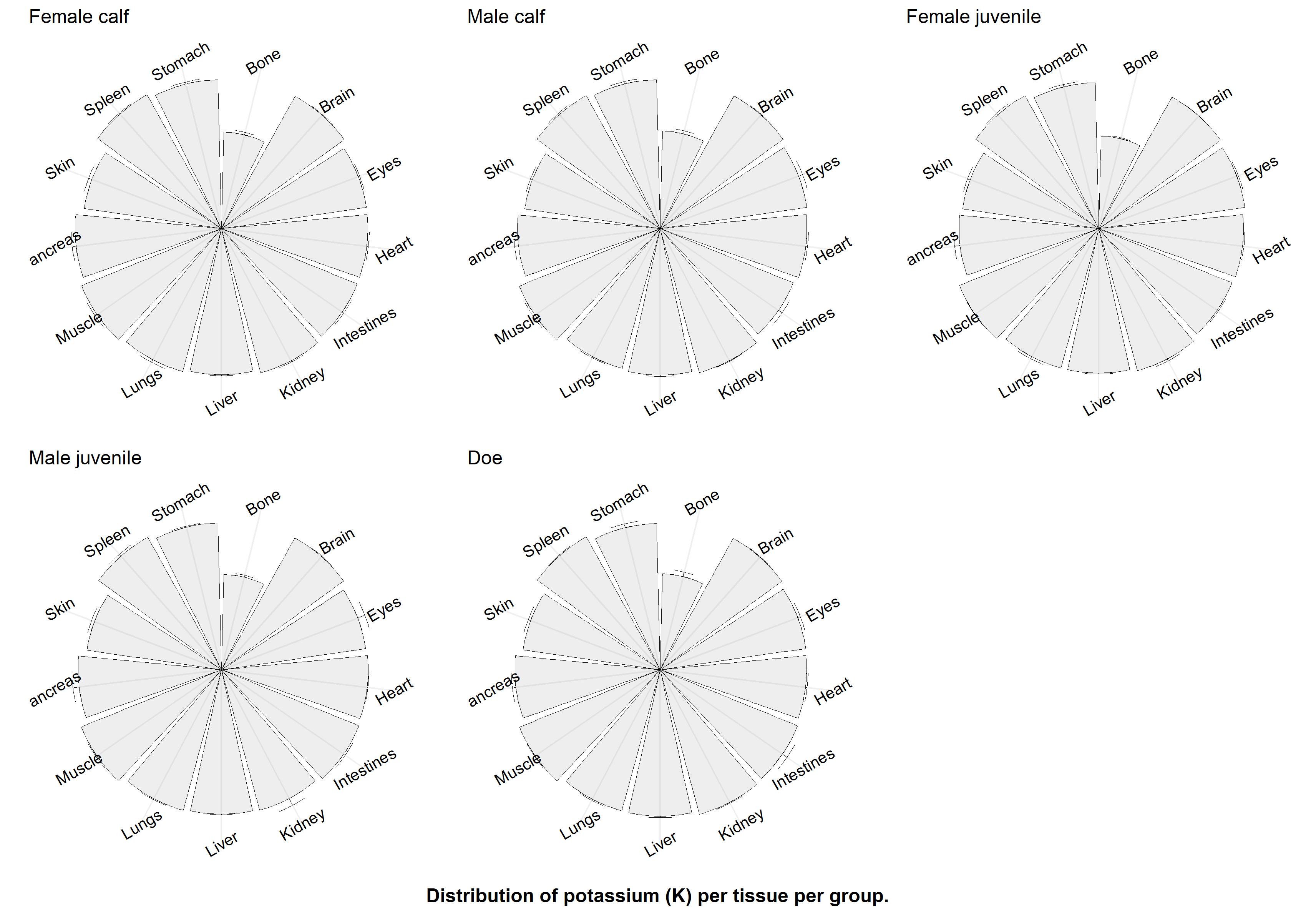


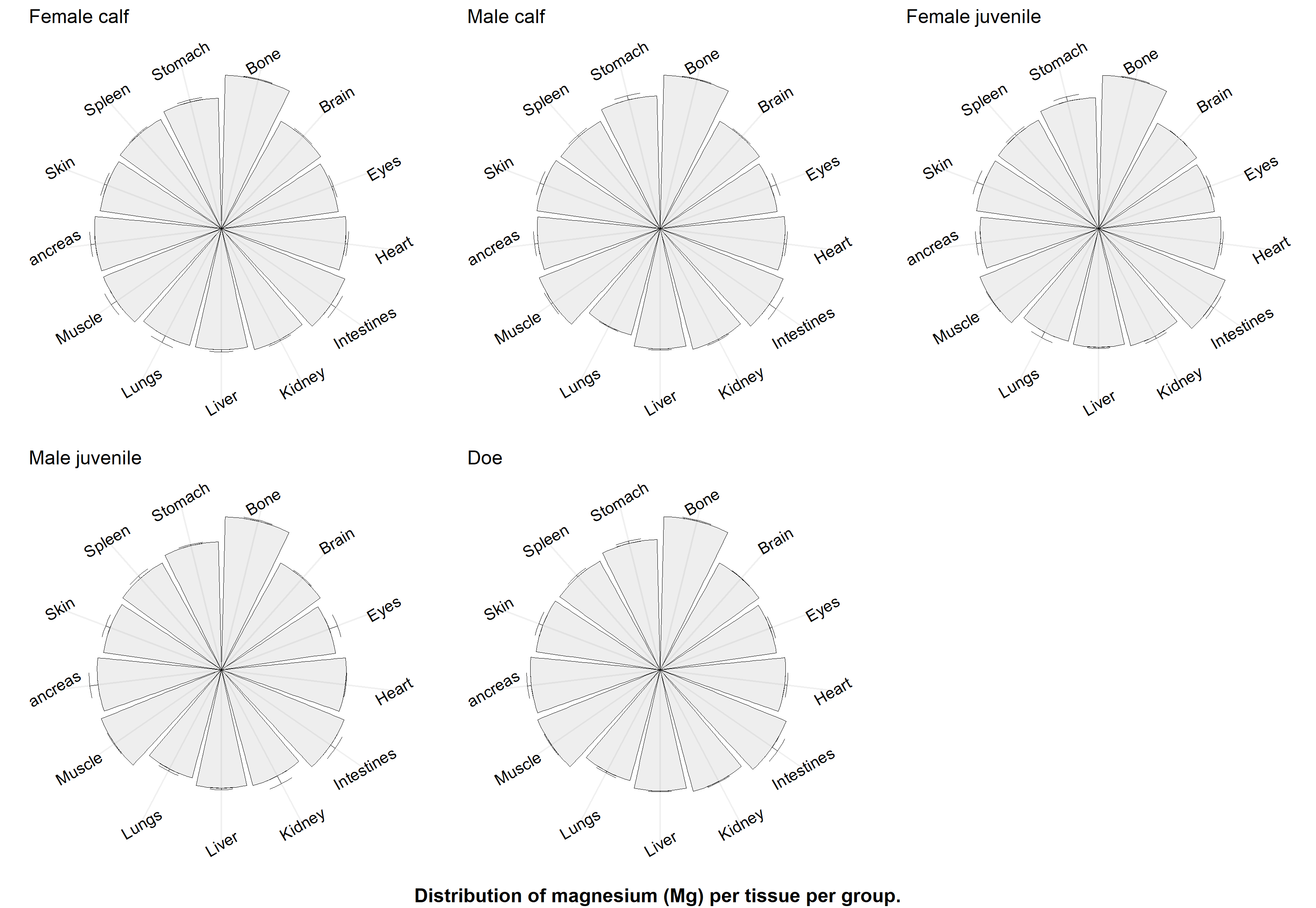


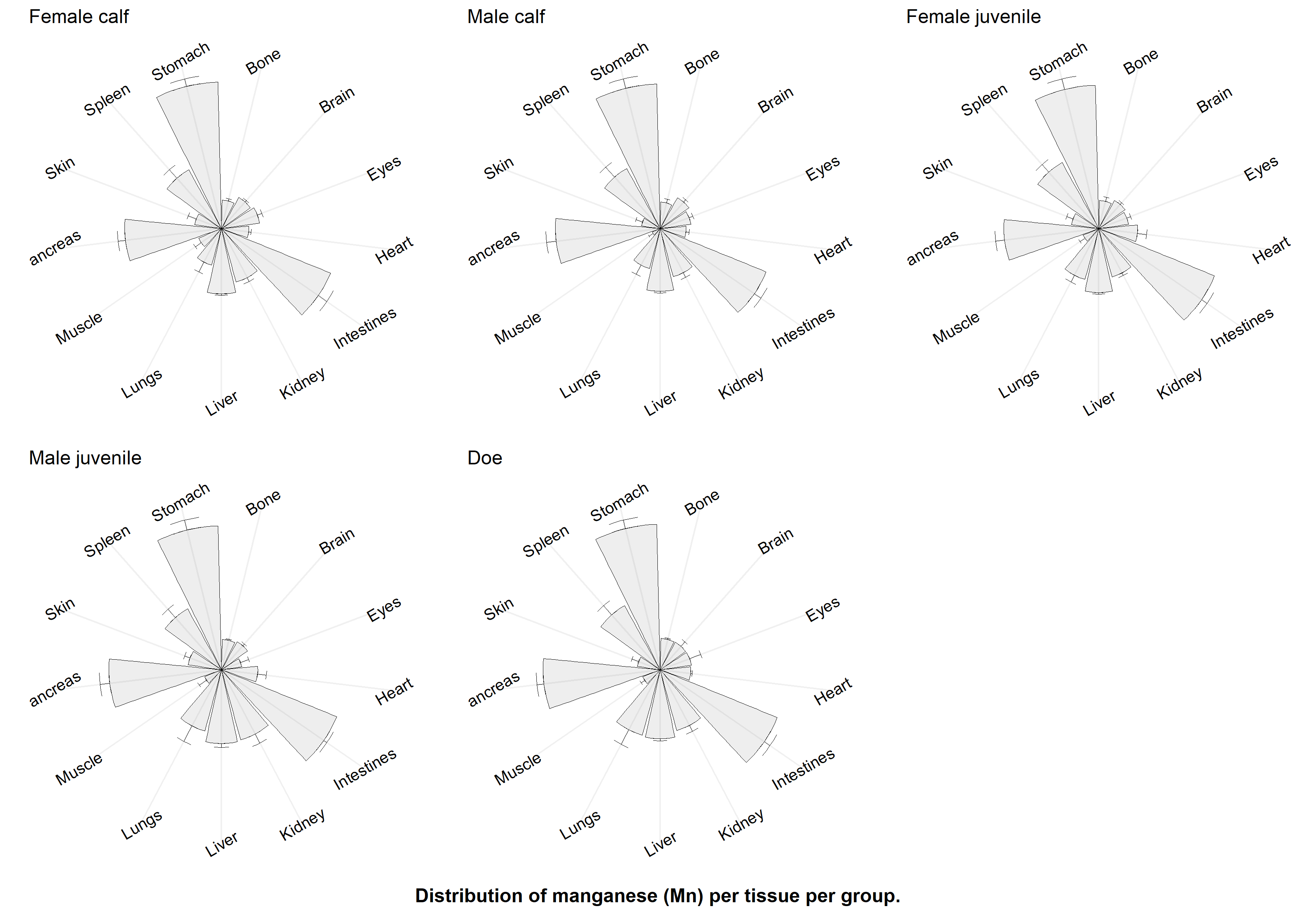


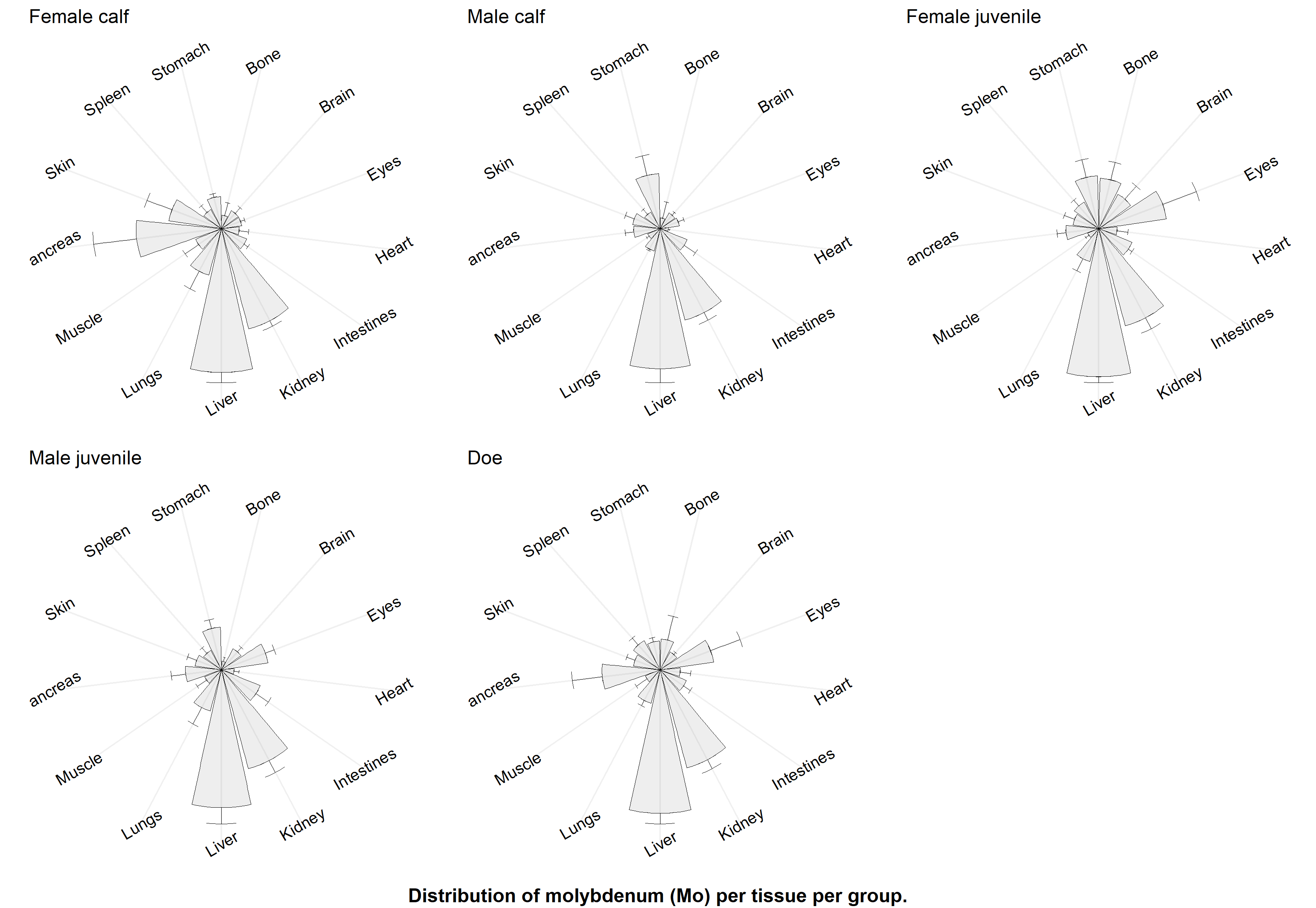


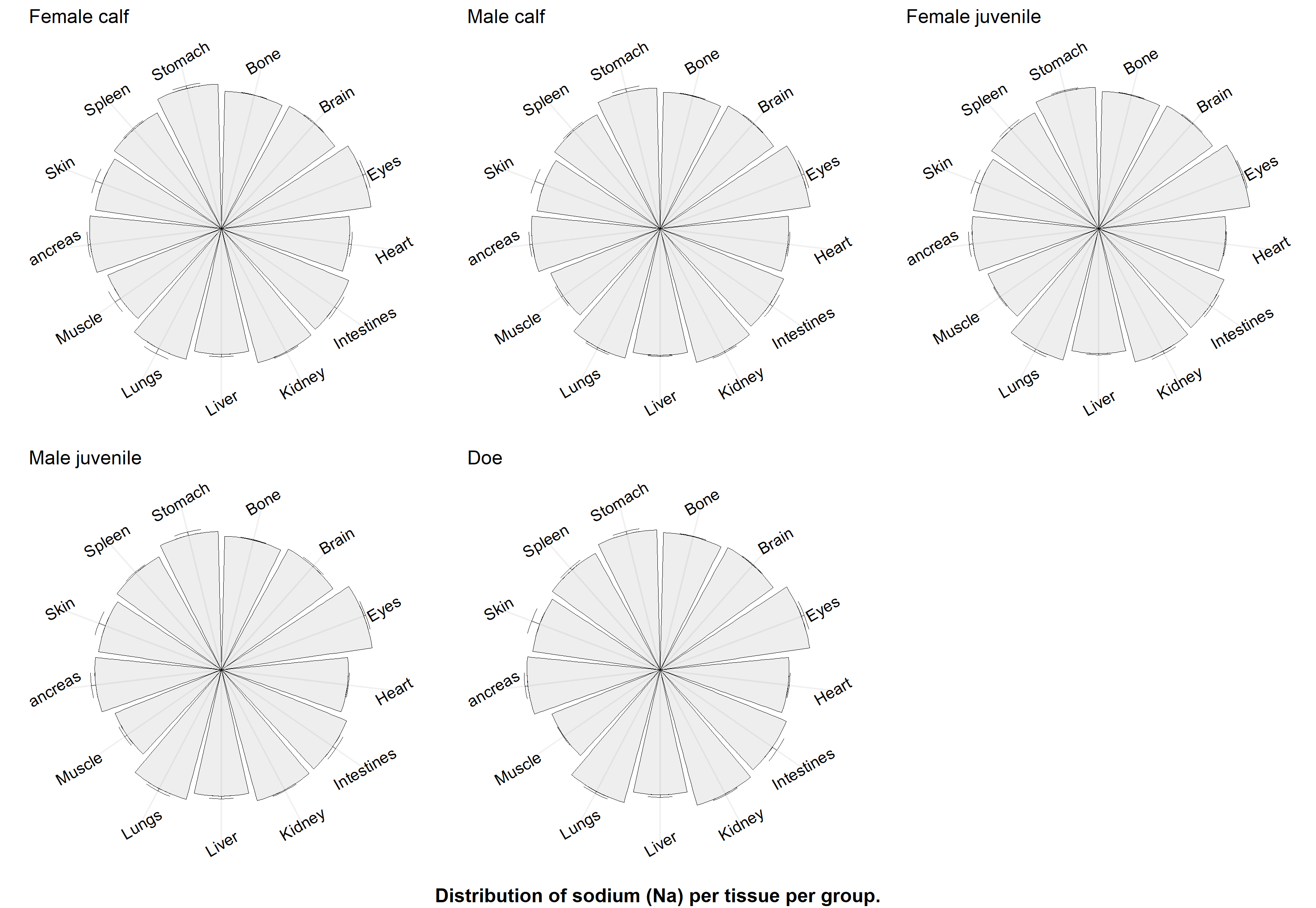


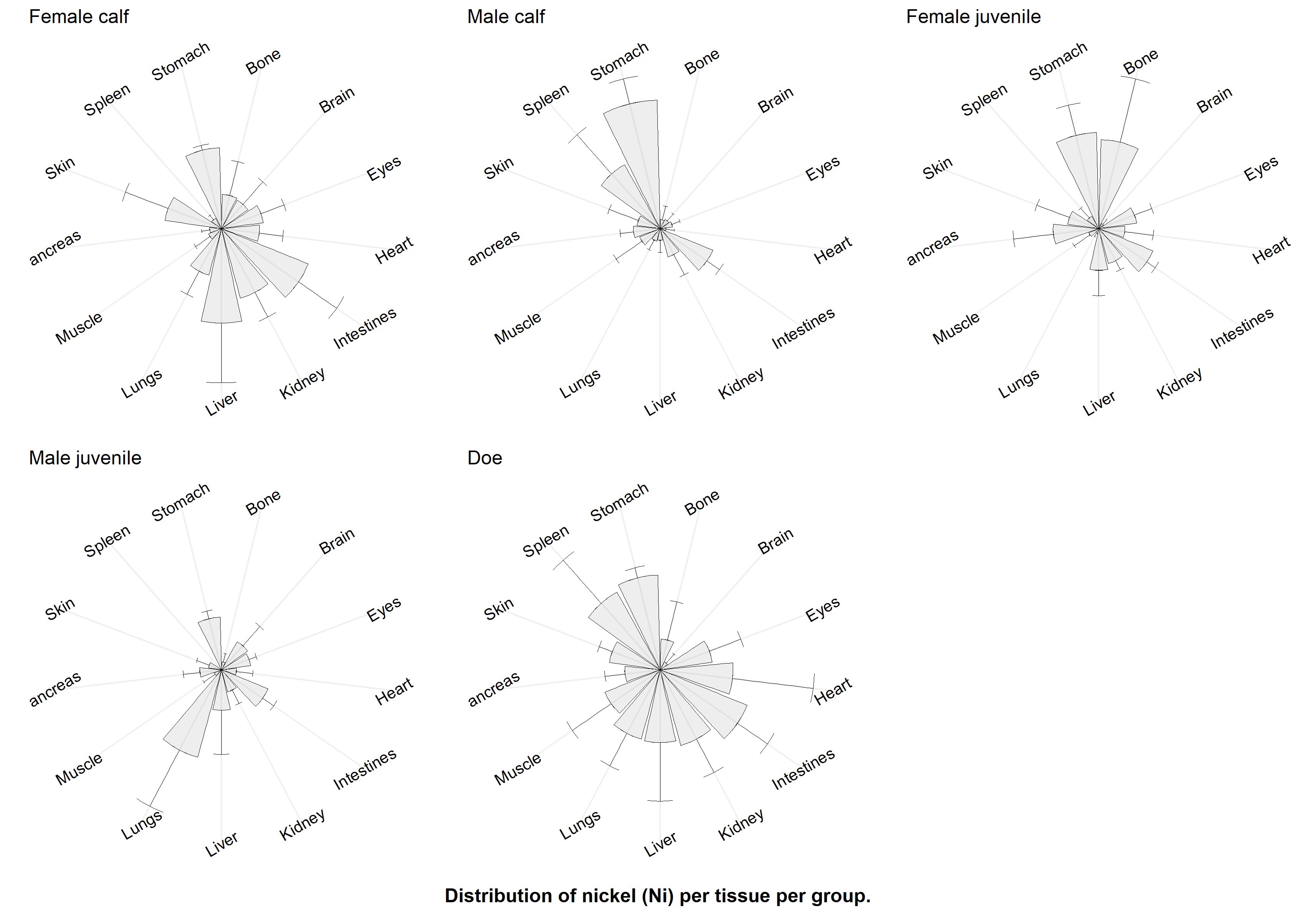

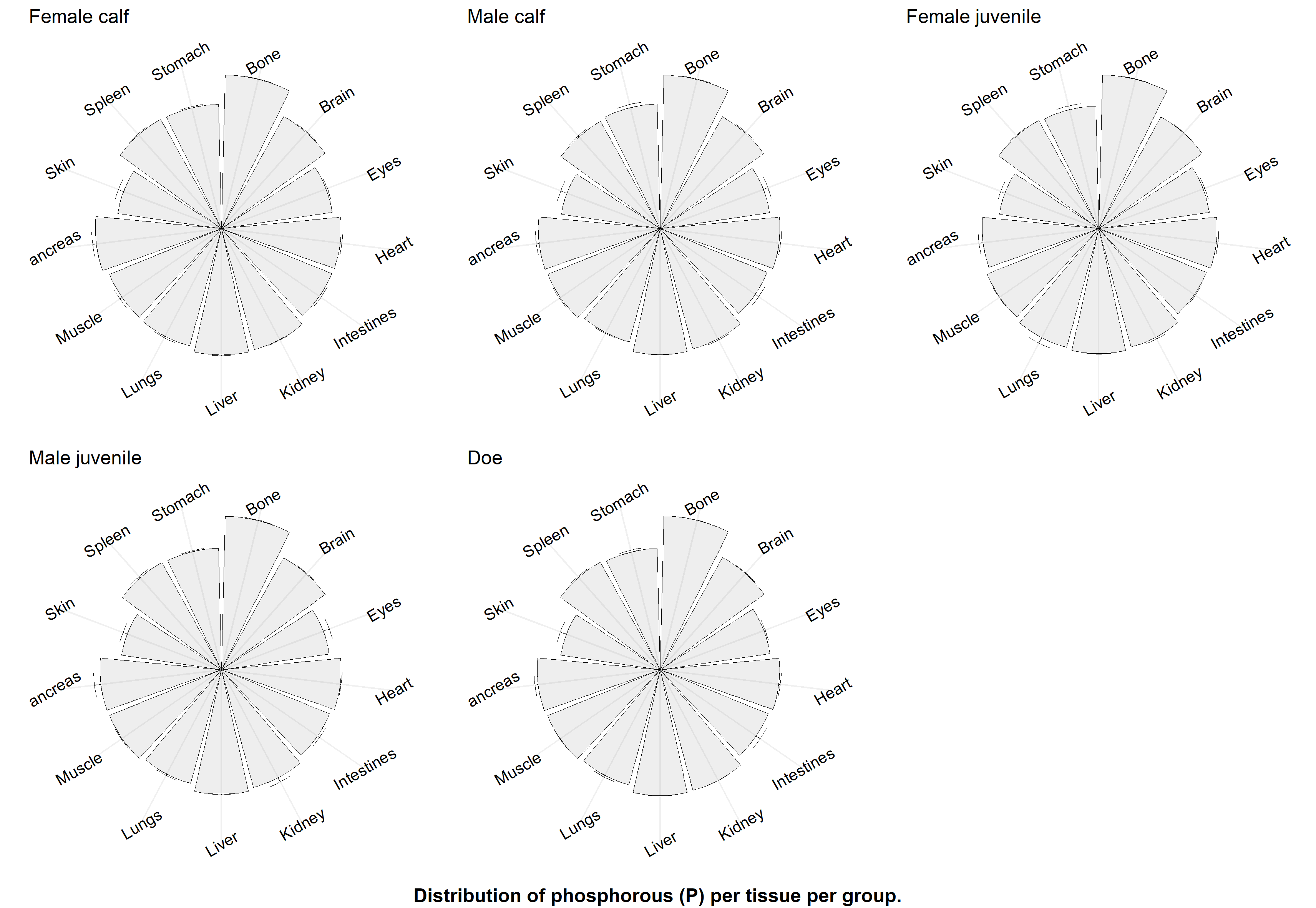

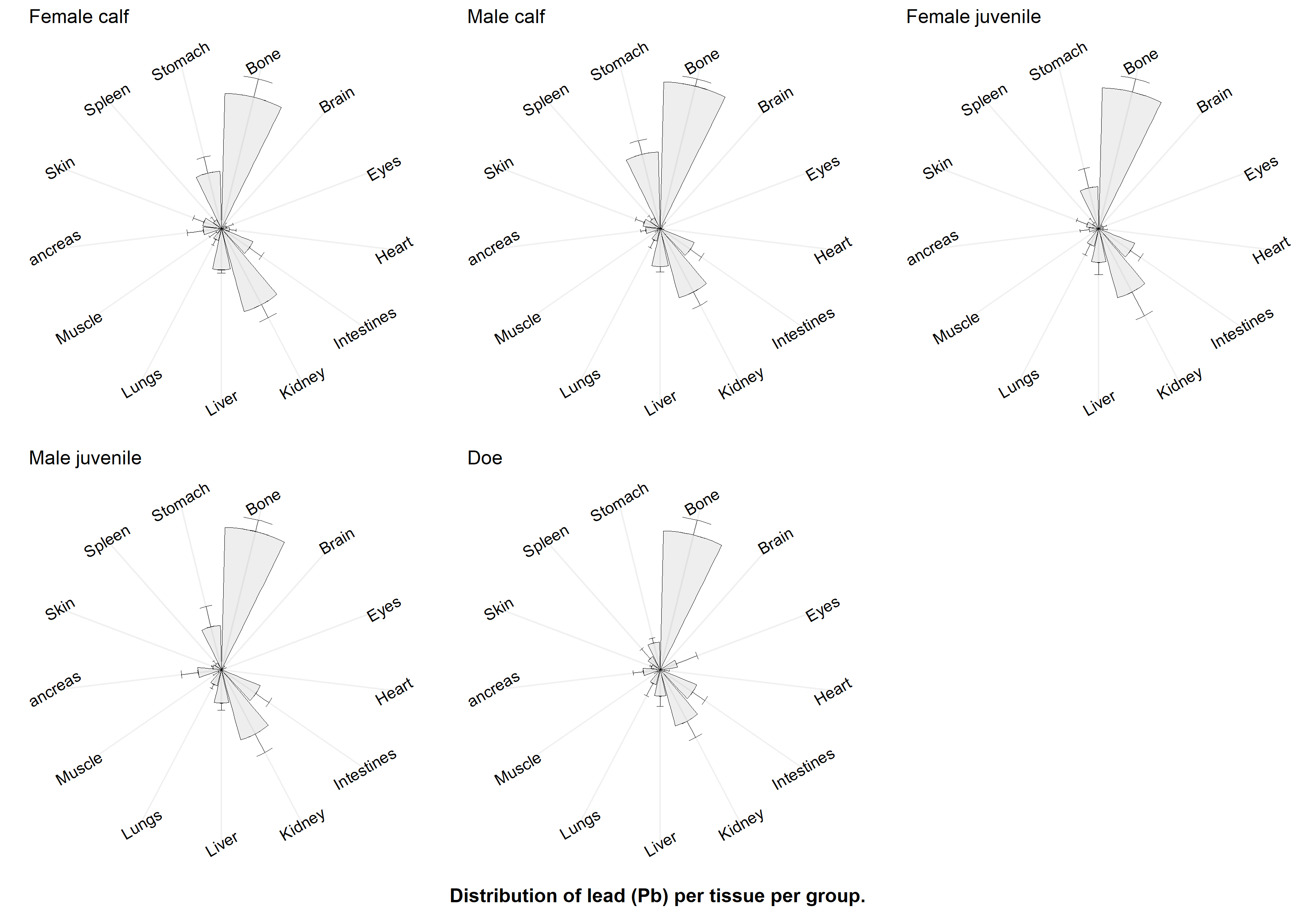

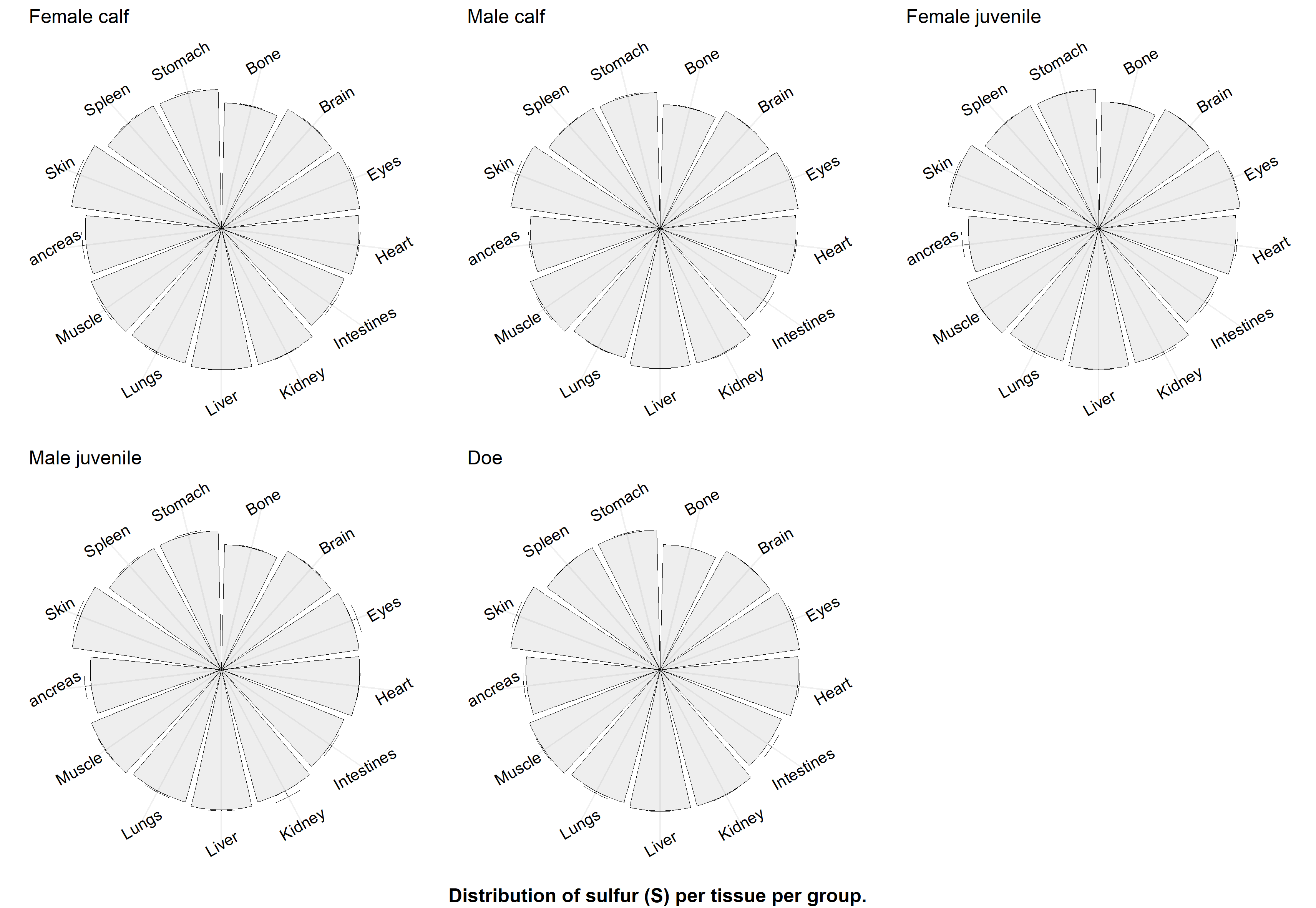

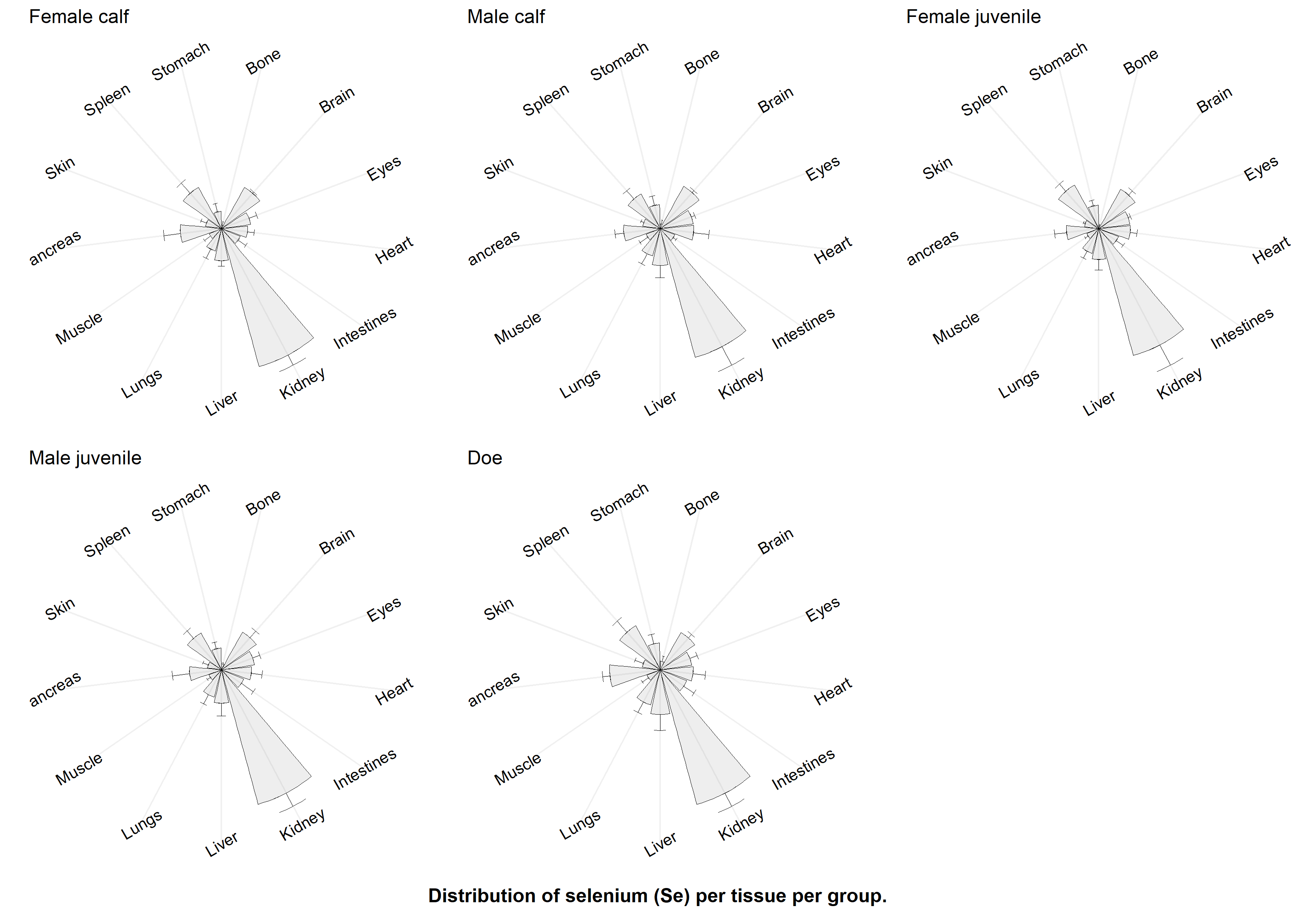

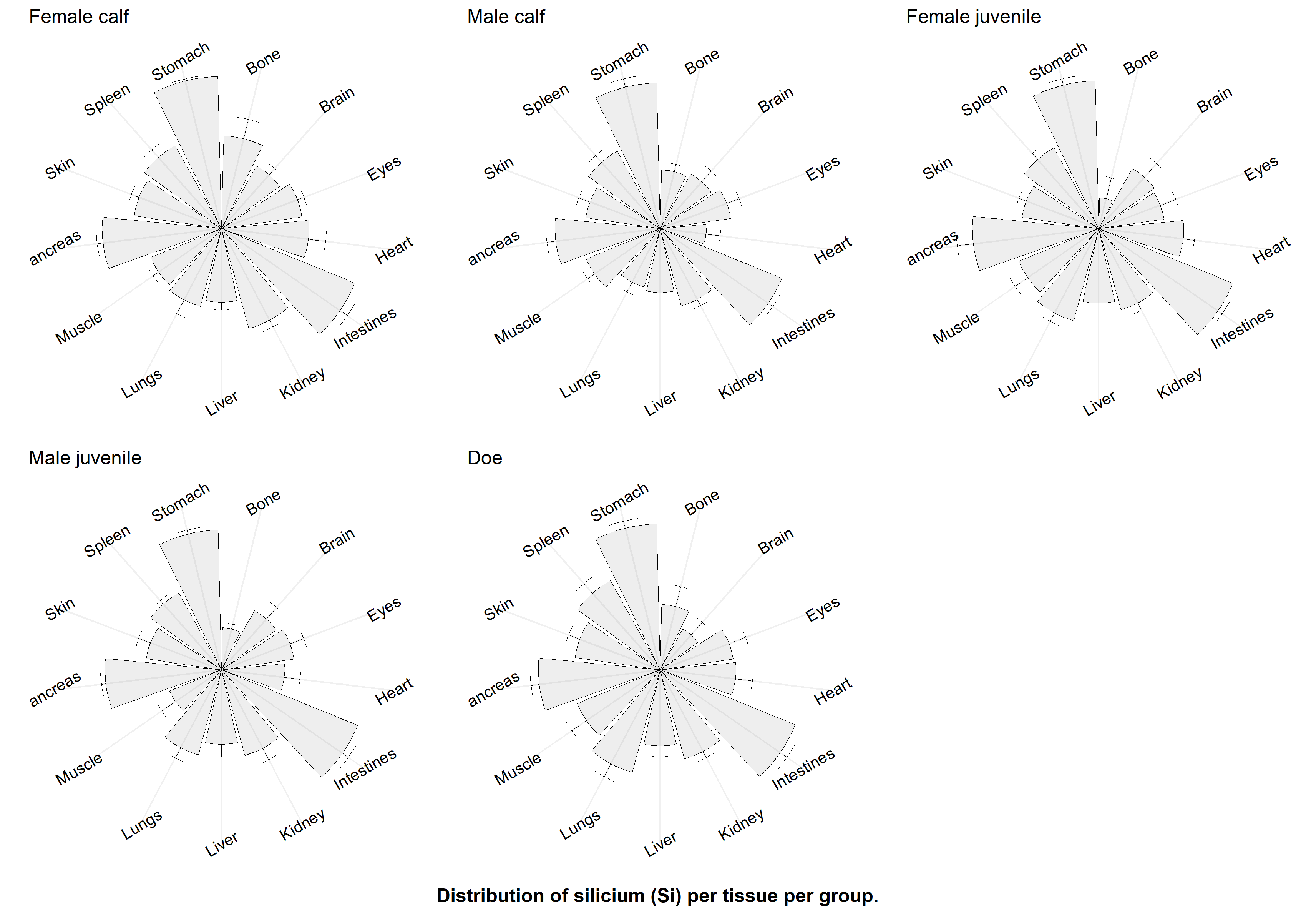

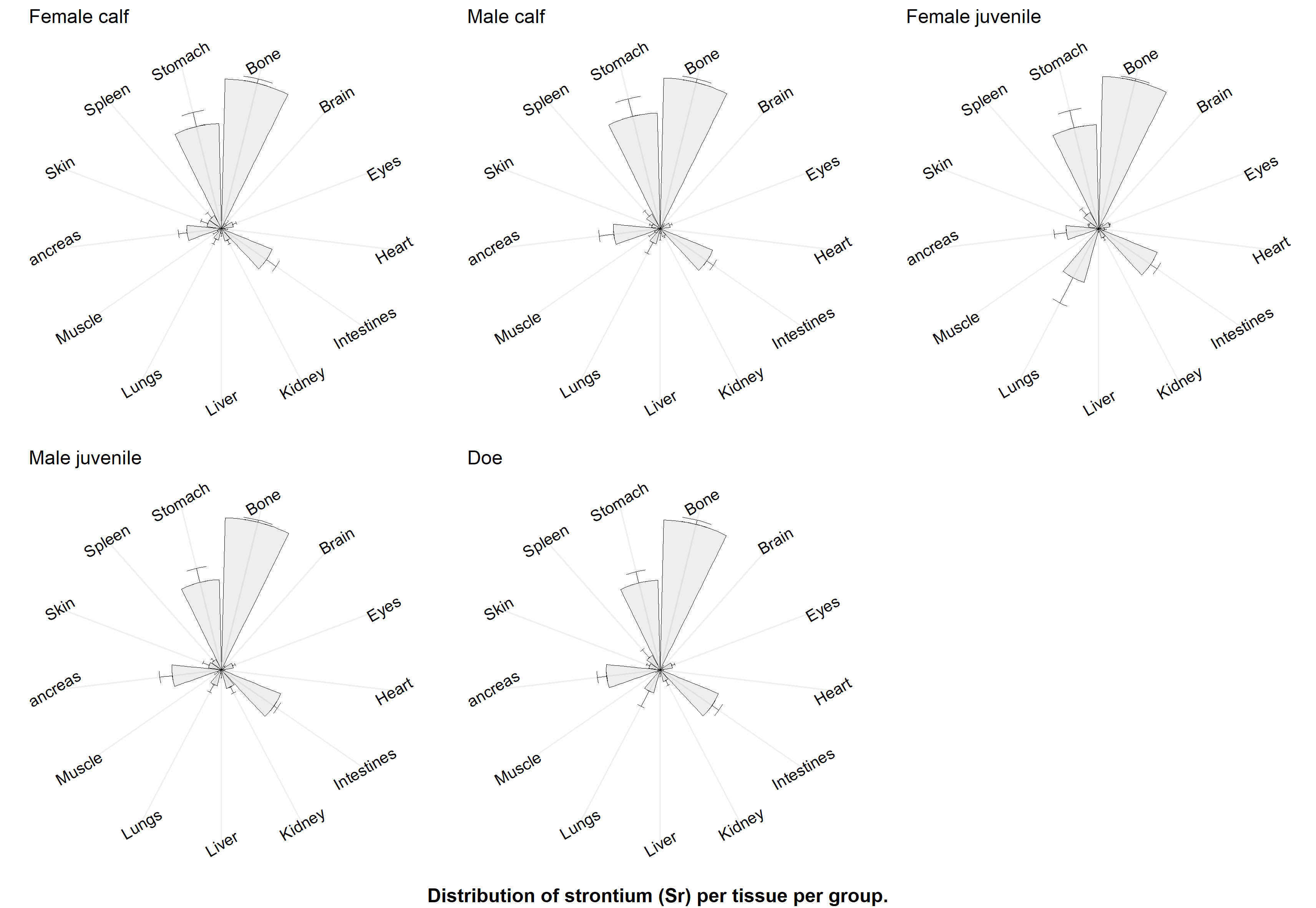

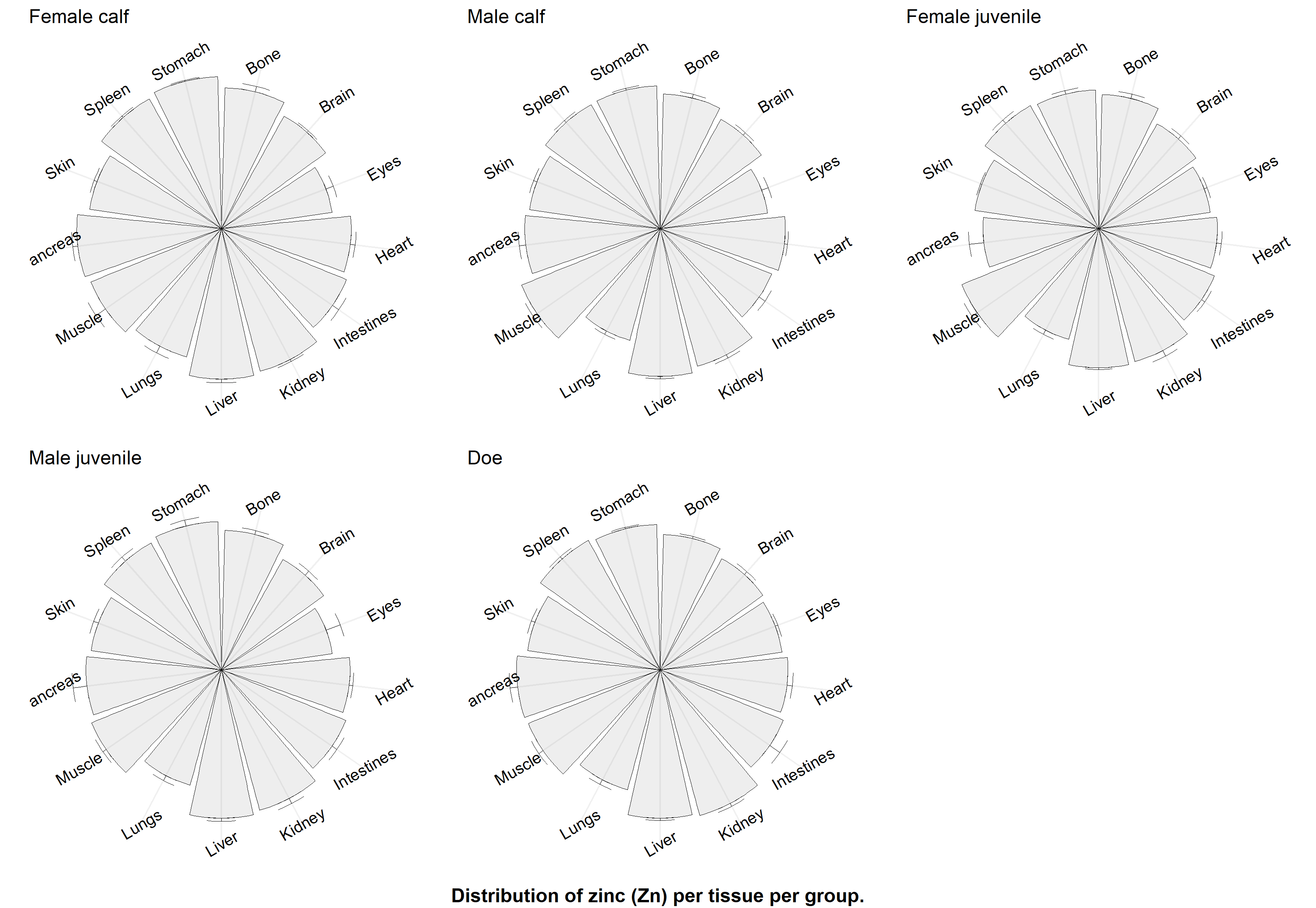


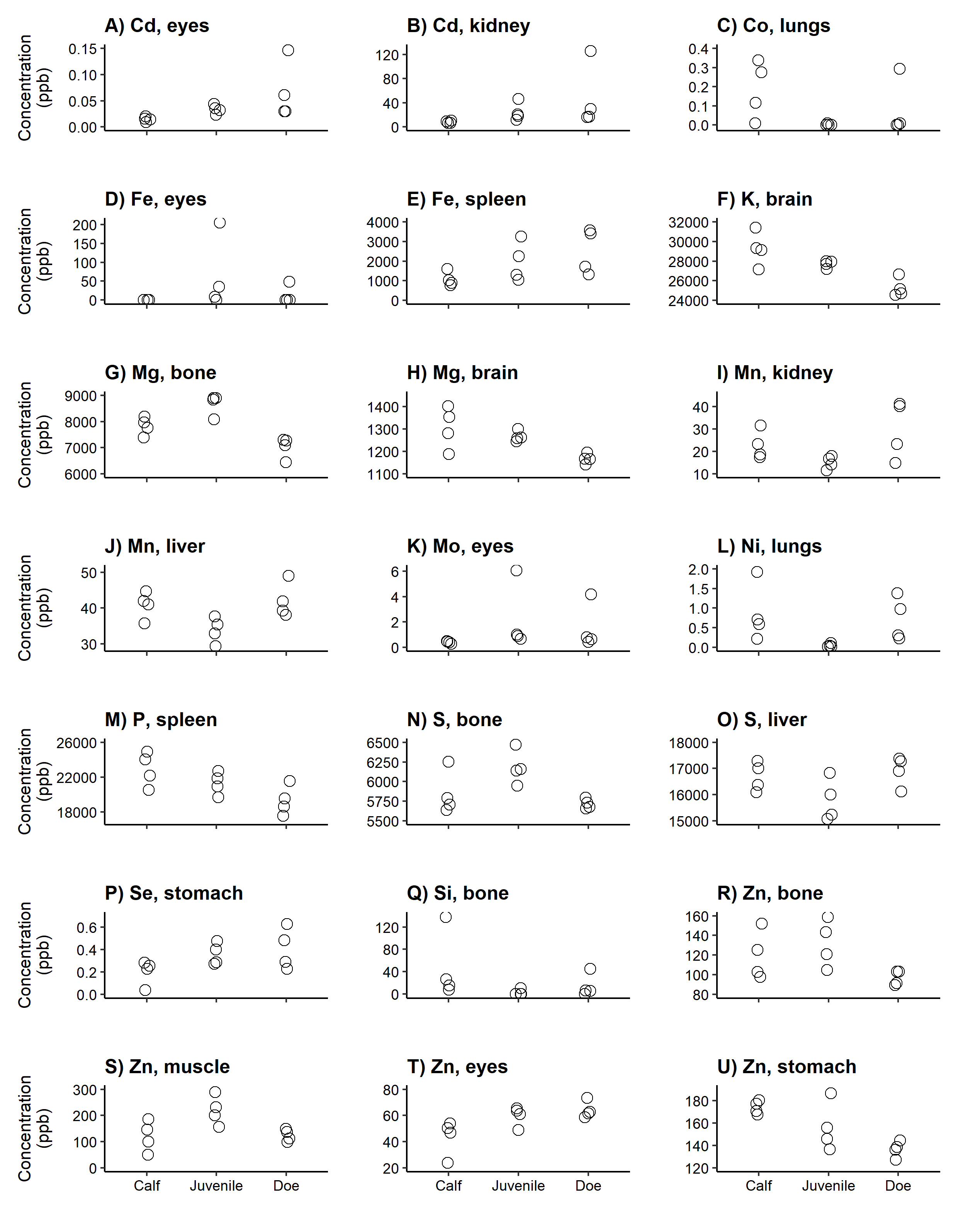


**Differences in females per age per tissue (after “DBH” correction).**


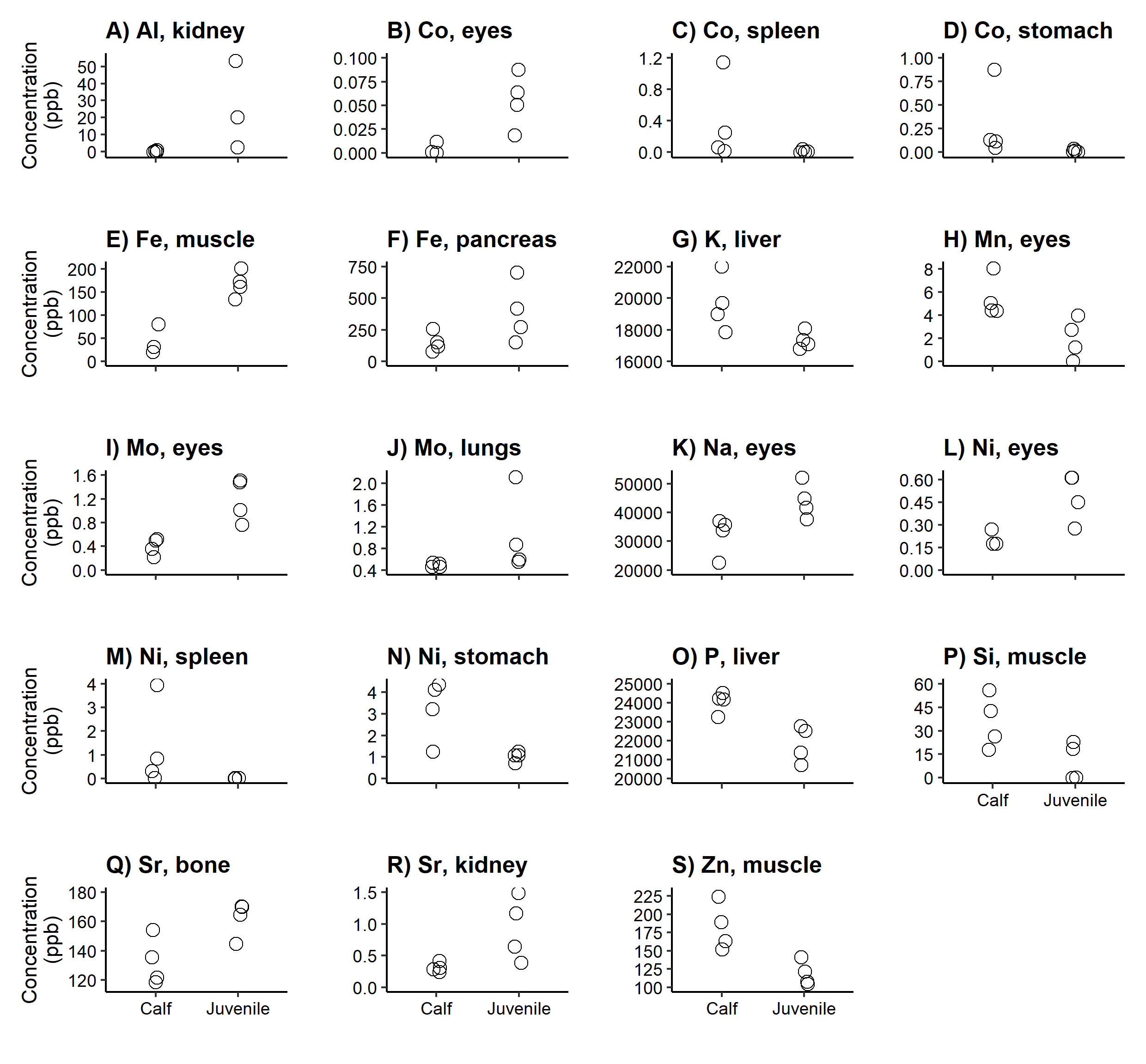


**Differences in males per age per tissue (after “DBH” correction).**


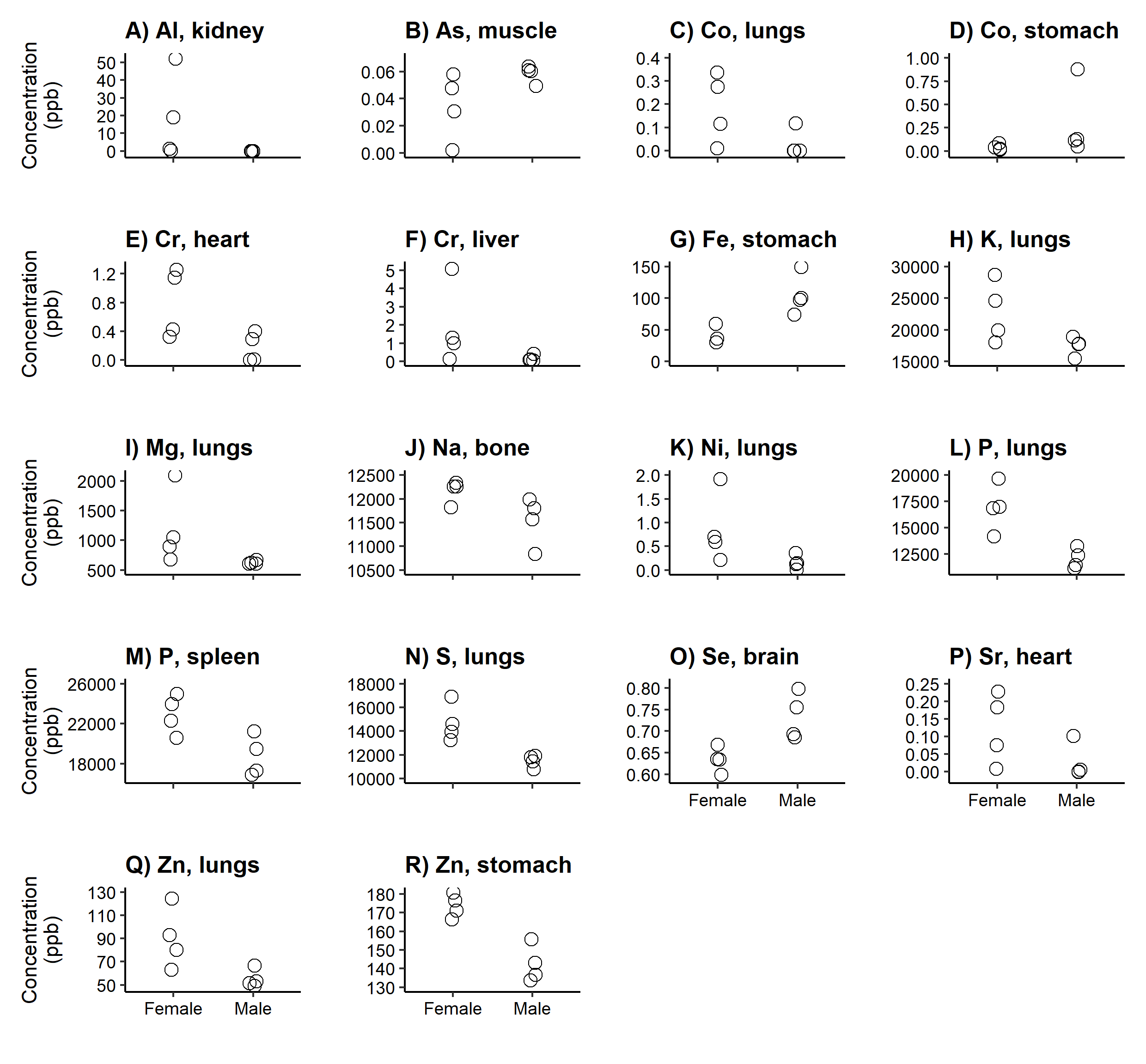


**Differences between females and males in calves per tissue (after “DBH” correction).**


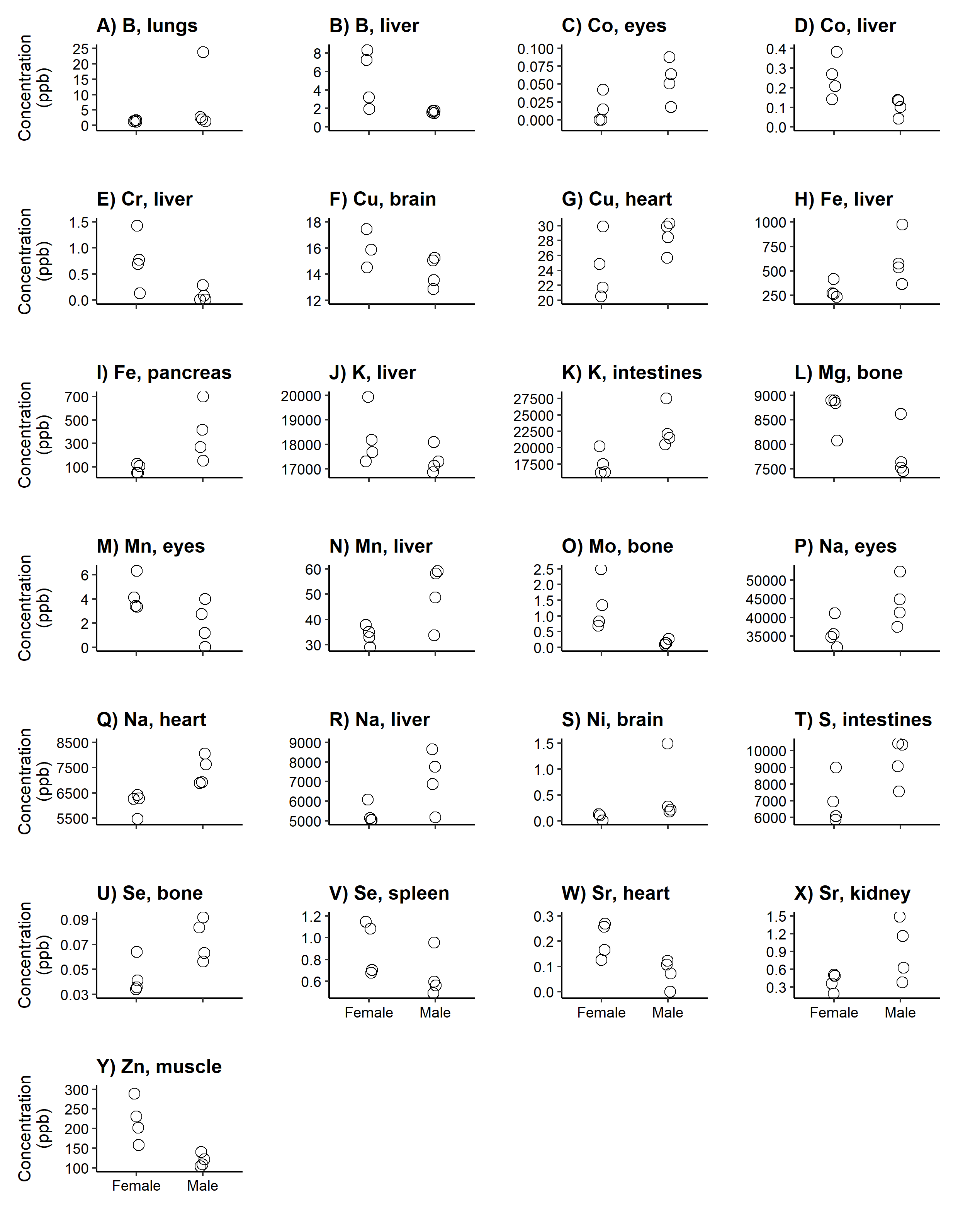


**Differences between females and males in juveniles per tissue (after “DBH” correction).**
